# Concise Guidelines of the European Cardiac Arrhythmias Society (ECAS) on “Catheter Ablation of Atrial Fibrillation”

**DOI:** 10.1111/jce.16561

**Published:** 2025-03-04

**Authors:** Riccardo Cappato, Samuel Levy, Rui Providencia, Hussam Ali, Andrey Ardashev, Sergio Barra, Antonio Creta, Michal Farkowski, Christian‐Hendrik Heeger, Prapa Kanagaratnam, Thorsten Lewalter, Silvia Magnani, Richard Schilling, Internal reviewers, Douglas Packer, Stefan Willems, Eli Ovsyshcher, Leonardo Calò, Feifan Ouyang, Ali Oto, Mark Estes

**Affiliations:** ^1^ Arrhythmia and Electrophysiology Department IRCCS MultiMedica Milan Italy; ^2^ Department of Cardiology, Marseille School of Medicine Aix‐Marseille University Marseille France; ^3^ Department of Cardiology, Institute of Health Informatics Research University College, and Barts Heart Centre, Barts Health NHS Trust London UK; ^4^ Department of Medicine, Feinberg School of Medicine Northwestern University Chicago Illinois USA; ^5^ Department of Cardiology Hospital da Luz Arrábida Gaia Portugal; ^6^ Department of Cardiology Ministry of Interior and Administration National Medical Institute Warsaw Poland; ^7^ Department of Rhythmology University Heart Center Lübeck, University Hospital Schleswig‐Holstein, and Asklepios Klinik Hamburg Altona, Clinical for Cardiology Hamburg Germany; ^8^ Department of Cardiology Imperial College Healthcare NHS Trust London UK; ^9^ Department of Cardiology, Hospital Munich South Munich Germany and Univ. of Bonn Bonn Germany; ^10^ Department of Cardiology San Paolo Hospital Milan Italy; ^11^ Department of Cardiology Barts Health NHS Trust and Welbeck Heart Health London UK; ^12^ Department of Cardiology Mayo Clinic‐St. Mary's Hospital Rochester Minnesota USA; ^13^ Kardiologie Askaelepios Krankenhaus Hamburg Germany; ^14^ Ben‐Gurion University of the Negev Beer Sheva Israel; ^15^ Department of Cardiology Casilino Hospital Rome Italy; ^16^ Department of Cardiology, State Key Laboratory of Cardiovascular Disease Fuwai Hospital Beijing China; ^17^ Department of Cardiology Memorial Hospital Ankara Turkey; ^18^ Department of Cardiology, The Heart and Vascular Institute, University of Pittsburgh Medical Center University of Pittsburgh School of Medicine Pittsburgh Pennsylvania USA

**Abbreviations**
AAD: anti‐arrhythmic drugs
ACT: activated clotting time
AF: atrial fibrillation
CB: cryo‐balloon
CFAE: complex fragmented atrial electrograms
CHF: congestive heart failure
CT: computerized tomography
CV: cardioversion
DC: direct current
ECAS: European Cardiac Arrhythmia Society
GPA: ganglionated plexi ablation
HFmrEF: heart failure with mildly reduced ejection fraction
HFpEF: heart failure with preserved ejection fraction
HFrEF: heart failure with reduced ejection fraction
ICD‐9‐CM: International Classification of Diseases‐Ninth Revision‐Clinical Modification
ICE: intracardiac echocardiography
LA: left atrium
LAA: left atrial appendage
LPW: left posterior wall
LSVC: left superior vena cava
LVEF: left ventricular ejection fraction
NIS: Nationwide In‐hospital Sample
NOAC: novel oral anticoagulants
PF: pulsed field
PV: pulmonary vein
RCT: randomized clinical trial
RF: radiofrequency
SVC: superior vena cava
TIA: transient ischemic attack
USP: United States Pharmacopeia
VKA: vitamin K antagonist
WACA: wide area circumferential ablation

## 1 Introduction

Guidelines have been introduced to provide “systematically developed statements to assist practitioner and patient decisions about appropriate health care for specific clinical circumstances” [1]. Since their introduction, they have been a valuable tool for a large population of stakeholders, including physicians, patients, hospitals, educational bodies, manufacturers, health providers and insurers in medicine.

While guidelines are meant to help professionals in taking routine clinical decisions, there are very few studies possessing the quality required to generate solid recommendations. Despite limited high‐quality evidence, guidelines typically consist of full‐bodied texts and tables reporting extensive lists of recommendation [2, 3, 4].

To compensate for lack of evidence‐based documentation, studies of incomplete quality and condensed judgment among authors are used to generate recommendations of intermediate class enriched with sub‐categorization based on multiple arbitrarily generated “levels of evidence”.

Frequent updating produced by different scientific bodies adds to the model complexity. In addition, guidelines are increasingly perceived as creating unjustified legal liability for practitioners despite the limited evidence on which they are based. To address this, processes have been proposed to impose rigor and transparency with which guideline documents are developed [5, 6] to assess the quality of guidelines, provide a methodological strategy for their development, and clarify what information and how this information should be reported in guideline documents [7, 8]. As a result, guidelines should contract in volume and re‐direct focus on recommendations based on clinical indications supported by more solid evidence. Criteria adopted to substantiate the need for high‐quality in our guidelines are described in paragraphs “Criteria for Class I, Class II and Class III recommendations” of **Chapter 5**.

Catheter ablation of AF represents a situation in which high‐quality studies are rather limited. Yet, guidelines and consensus documents have resulted in the production of copious documents [2, 3, 4, 9].

With the intent of producing a guideline that serves its intended purpose and reconciling with the art of medicine, the European Cardiac Arrhythmia Society (ECAS) proposes a concise and coordinate document to assist practitioner and patient decisions in this field. ECAS is an independent society founded in 2004 in Paris with the mission to promote the discipline of better care in diagnosing and treating cardiac arrhythmias. The criteria used in this document to define recommendation classes are summarized in the paragraph “criteria for recommendations” below.

### 1.1 Notes About Manuscript Production

This Guideline document was registered on PREPARE (Practice guideline Registration for transparency; registration number: PREPARE‐2024CN005) in February 2024.

Multiple searches for randomized controlled trials to inform this document were performed. These were done as part of purpose‐built systematic reviews which have meanwhile been published as separate manuscripts and present information on the detailed search strategy, PICO approach and questions addressed by the Guideline and each of the Reviews. Randomized controlled trials meeting the inclusion criteria for our systematic reviews were included. Additionally, randomized controlled trials not addressing other aspects of the guideline not covered by the systematic reviews, but that were identified through the search process were also considered eligible [10].

An update of the guidelines is planned, with the definition of guideline questions and search starting, up to 5 years following the publication date of the present document. An earlier update may occur, if justified, driven by novel technology or publication of impactful evidence on a field not covered by this document. In such circumstances, the decision may be for updating only that section of the guideline, rather than the whole document.

## 2 Truth in Medicine and Revisitation of the Term “Recommendation”

“The logic of science is hypothetical. To be powerful, science required to turn its back to the incontrovertible thruth. No matter how strong their degree of confirmation, scientific hypotheses can be ‘falsifiable’ at any time” [11].

*Emanuele Severino*, philosopher *‐ Truth in medicine*

Definition of truth in science has been subjected to considerable modifications over time [12]. In the 2nd century A.C., Ptolemy proposed a cosmologic model according to which the earth would stand motionless at the center of universe. It took 13 centuries before Copernicus proposed a new, revolutionary model supporting the concept that the earth would rotate around the sun and one more century before Copernicus theory became widely accepted [13]. In the present era, technological development accelerates progress in science and communication. As a result, even when created using high‐quality science, guidelines become out of date quickly. Therefore, it is inevitable that current methods for producing guidelines will be reviewed and changed in the future.

The universal adoption of evidence‐based medicine has recast medical knowledge in such a way that experimental studies designed to validate single isolated interventions take on the highest status and, by so doing, undermine clinical judgment and lock in place a reductive model of health and disease [10, 13].

In the present document, evidence‐based medicine will be used solely as a reference point for management of AF. This is consistent with the methods used by other societies in guidelines preparation and is taken under the assumption that new paradigms may surface in the future that offer different base theories, methods of investigation and validation that may subvert the current guideline's structure.

### 2.1 Revisitation of the Term Recommendation

First introduced in old French speaking as a derivation of the Medieval Latin *recommendationem* (nominative *recommendatio*), the word recommendation is intended as the “act of representing (something) in a favorable manner, the act of recommending (someone or something) as worthy” [14]. While consistent with this definition in the early days, guideline recommendations have lost their original meaning as they have escalated to a level close to imperative statements.

At present, practitioners tend to refer to recommendations in guidelines as persuasive obligations in response to which they feel bound to behave irrespective of the robustness of the source data. Accordingly, legal controversies over practitioners' behavior tend to be resolved by resorting to guidelines as to the ultimate form of judgment.

We argue that recommendations in medicine should continue to reflect the “act of representing selected indications in a favorable manner” rather than persuasive obligations. We also argue that favorable representation should apply only to conditions that are substantiated by evidence‐based documentation rather than arbitrary criteria reflected in further sub‐categorization classes of recommendation or consensus among experts. In keeping with these aims, we propose a linear concise scheme of guideline creation. The criteria which guided this approach scheme have been described previously and are also reported in **Chapter 5** of the present document [10]. We believe that the present model best suits the purpose of serving the medical community while preserving the original significance of the term recommendations.

### 2.2 Use of Guidelines in Legal Trials to Resolve Medical Disputes

Guidelines are currently used as standard reference to resolve medical disputes in legal trials. We advocate for the elimination of this attitude based on the following statements:

‐ guidelines are meant to assist, not to impose practitioner decisions about appropriate health care;
‐ the proportion of high‐quality studies possessing the consistency required to generate solid guideline recommendations is rather limited in most medical disciplines;
‐ documentation from studies of incomplete quality represents the dominant source of information in guideline documents;
‐ most guideline recommendations are based on expert consensus at multiple unstudied, sub‐categorization levels, introducing arbitrariness to final decisions;
‐ limitations above are further amplified by incomplete information about methods and criteria with which recommendations are obtained in gray areas of knowledge;
‐ to compensate for such limitations, dedicated instruments are being proposed to favor more rigor and transparency in guidelines production.

## 3 Electrophysiological Rationale of Current AF Ablation Techniques

### 3.1 PV Electrical Isolation

The technique of PVs electrical isolation, first introduced by Haissaguerre et al. [15, 16], serves four fundamental purposes: (1) it does not require ongoing AF to be successfully accomplished [16]; (2) it segregates all arrhythmogenic foci and local circuits within the PV muscular layers possibly precipitating sustained AF episodes [16] (3) it reduces the overall mass of electrically active atrial tissue [16]; (4) it provides a highly reproducible and consistently verifiable technique in the setting of an EP procedure [15]. Since its introduction in 2000, this technique has been adopted on global scale and represents a qualifying mandatory strategy for catheter ablation of AF of any type (i.e., paroxysmal, persistent and long‐standing persistent). PV electrical isolation can be achieved with different energy forms (RF [16, 17], CB [18, 19], Laser [20, 21, 22], PF [23, 24]) and catheter designs (single tip [17], balloon [20, 21], basket‐flower [24]).

While segregation of arrhythmogenic foci within the PV muscular layers is effective in variable proportions of patients with AF, outcomes cannot be predicted in the individual patient. Two factors likely contribute to this observation: (1) recurrent PV‐to‐left atrium electrical conduction across the PV isolating line in the days/months after the nominal procedure [25] (2) location of the arrhythmogenic foci precipitating AF outside the segregated area [25].

In selected cases, stability of isolating lesions over time can be accomplished by means of multiple procedures [26]. The efficacy in establishing stable PV isolation does not appear to be dependent on energy and catheter design forms [24, 27, 28].

### 3.2 Ablation of Extra‐PV Triggers

Evidence in favor of recurrent arrhythmia in patients with stable PV electrical isolation over time indicates that triggers for AF precipitation must originate from non‐segregated areas of atrial tissue [29]. Complimentary designs have been introduced to ablate these areas, including ablation of ganglionated plexi [30] and ablation of spontaneous or catecholamine‐elicited foci [29, 31, 32], ethanol infusion within the ligament of Marshall [33] and LAA isolation (LAA) [34, 35]. In the case of catecholamine‐elicited foci, ablation of atrial foci also represents an opportunity when patients present with reproducible spontaneous atrial ectopic beats in the course of the EP procedure [32]. The efficacy of these techniques awaits evidence‐based documentation, especially in light of: (1) the inability to accurately identify and effectively ablate all ganglionated plexi in the individual patient [30]; (2) the sporadic prevalence of spontaneously occurring atrial ectopic beats or AF precipitation in the course of the EP procedure [31]; (3) the non‐clinical nature of drug‐induced arrhythmias [32]; and, 4) the lack of a standardized technique enabling accurate evaluation of the acute efficacy of spontaneous or catecholamine‐elicited extra‐PV foci ablation [31].

### 3.3 Linear Lesion Ablation

The rationale of linear lesion ablation is based on the intention to replicate the compartmentalization technique (MAZE procedure) successfully introduced in cardiac surgery in the late 90 s [36]. This technique consists in point‐by‐point deployment of ablation pulses until a continuous (transmural) ablation lesion is constructed between two separate anatomical boundaries [36]. This technique offers three potential advantages: (1) it may be deployed in sinus rhythm as well as during AF; (2) it enables compartmentalization without the need of open‐heart surgery; (3) it provides the option of “customizing” the intended design to the special need of the individual patient. The ability to deploy linear lesions during the ongoing arrhythmia makes this technique appealing in patients with persistent and long‐standing persistent AF. In spite of its potential benefit, linear lesion ablation has failed to prove effective in several randomized studies [37]. Conversely, linear lesion ablation is associated with an increased propensity to favor macro‐reentrant atrial tachycardias during follow‐up [38]. This circumstance represents an undesired effect of the ablation technique and is commonly related to conduction recurrence possibly occurring during follow‐up across one or multiple gaps along the ablation line deployed at time of the acute procedure [38].

### 3.4 CFAE Ablation

The rationale of CFAE ablation stems on the hypothesis that localized areas within the atrial tissue are critical to enable maintenance of paroxysmal or persistent AF by means of a “functional” re‐entry‐dependent mechanism [39]. EP recording at those same sites during sinus rhythm typically fails to show CFAE or other abnormal potentials. The functional character of the re‐entrant circuit is also outlined by the fluctuations, albeit limited, in recording position and morphology of CFAE [40]. Ablation is delivered in areas where CFAEs show a reproducible time and electrogram reproducibility beyond 5–8 s during continuous recording [41]. According to this model, one or more circuits may be responsible for maintenance of clinical AF [39]. Ablation of these areas would at some point lead not only to termination of ongoing AF episodes, but would also preclude maintenance of new sustained AF episodes [39].

CFAE ablation is severely limited by several factors. Among them are: (1) variable definitions of CFAE among investigators [40, 41]; (2) variable durations of analysis time (i.e., the time during which CFAE is tested to show re‐iteration and reproducibility of the qualifying signal) depending on the manual or computer‐assisted algorithm used; and, (3) variable automatic detection algorithms incorporated in the various computer‐assisting tools available in the catheter lab [40, 41] More substantially, the hypothesis of a geographically limited re‐entrant circuit that enables maintenance of AF episodes conflicts with the solid model of the multi‐wavelet and multi‐layer activity shown during continuous electrogram monitoring in human beating hearts.

Clinical experience with CFAE ablation has been rather inconsistent. The promising data reported in the first study [39] have not been replicated elsewhere [40, 41] and the use of this technique awaits more solid evidence before it can be proposed on large scale.

### 3.5 Rotor Ablation

Rotors were introduced in 1990 [42] as phase singularities occurring in anatomically identifiable areas in the atria and responsible for maintenance of AF episodes. According to the proposed theory, these are regions of extreme wave curvature as the center of functional stable reentry where conduction velocity approximates zero and can be detected by phase mapping [43]. Multiple rotors may be present simultaneously leading to distal wavefront collision, thus contributing to the appearance of global disorganization. Phase singularities are sites about which all phases of the depolarization/repolarization cycle exist simultaneously and are important because they identify tissue capable of supporting rotors steadily. With the introduction of computational modeling [44], 3D mapping allowed identification of the linear structure of phase singularities, termed filament, spanning endocardium to epicardium with various configurations (I, U and O).

Clinically, the most tangible feature of a rotor is repetitive, cyclic activation around a core [45, 46]. While this is the simplest visual criterion for identifying rotors in phase maps (FIRM) or isochronal images, it does not capture the essence of detecting or defining a rotor. Ablation of sites where rotors are identified may result in termination AF episodes.

## 4 Classification of AF Type Related to Catheter Ablation

Given the impact that the clinical presentation of AF plays on the outcomes and, therefore, the techniques used for catheter ablation, it is important to have a valid classification of AF type. Of the many models proposed, the most appropriate for the purposes of catheter ablation appears to be the one based on temporal duration of single AF episodes [47]. While classification into paroxysmal (i.e., AF that terminates spontaneously, with or without AADs, within 7 days from onset), persistent (i.e., continuous AF that is sustained beyond 7 days and for no longer than 1 year in spite of AADs, or that is susceptible to successful cardioversion) and long‐standing persistent AF (i.e., continuous AF that is sustained beyond 1 year in spite of AADs) is simple and intuitive, it should be noted that accurate categorization within these groups is not always easy, as episode duration may vary in the individual patient and are not always symptomatic. In the same individual, AF may fall into one category from a clinical point of view but may fall into another category when based on continuous monitoring [48]. Similarly, dissociation between AF‐type and pathophysiologic background (i.e., substrate fibrosis) has been documented [49].

While we recognize the value of categorization into paroxysmal, persistent and long‐standing persistent AF, ideally guideline recommendations should use the episode duration characteristics by the referenced studies. Because definitions differ even within the same sub‐category of AF (paroxysmal, persistent or long‐standing persistent), the present document will provide a detailed list of references indicating those studies that contributed to generate our recommendation scheme. In these studies, readers will find the AF type definition provided by the authors. It will be the reader's decision whether to apply these recommendations based strictly on the AF definitions reported in the original sources, or whether they are applicable to patients with similar if not identical clinical presentations.

## 5 Recommendations on Efficacy

**Figure 1:**
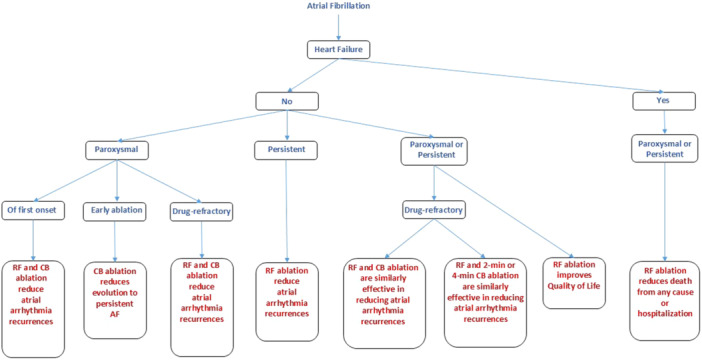
Flowchart highlighting clinical conditions and catheter techniques for which catheter ablation of AF is a Class I recommendation. AF, atrial fibrillation; CB, cryoballoon ablation; RF, radiofrequency ablation.

**Figure 2:**
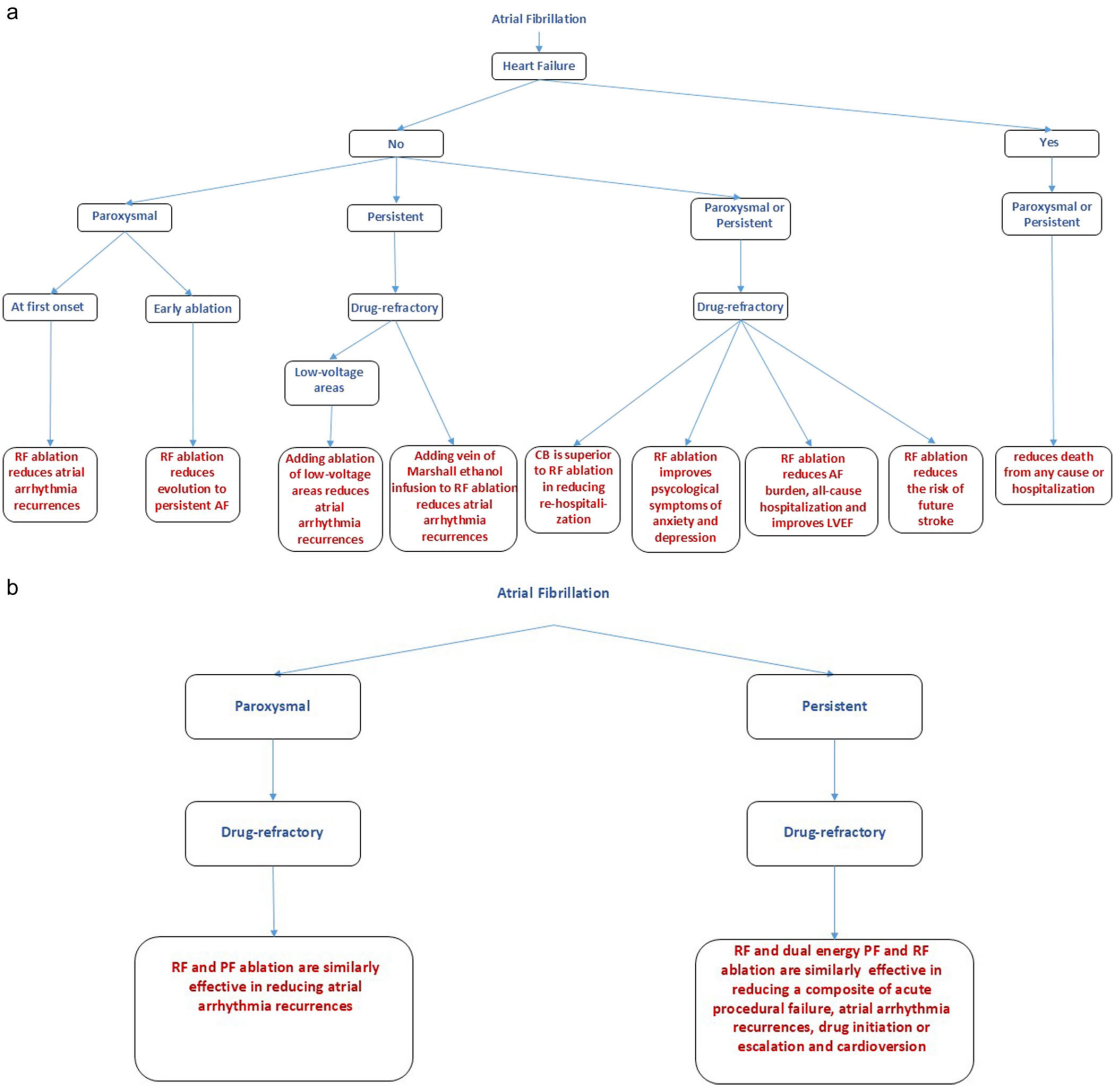
(a) Flowchart highlighting clinical conditions and catheter techniques for which catheter ablation of AF is a Class II recommendation (evidence for superiority of catheter ablation versus control therapy). (b) Flowchart highlighting clinical conditions and catheter techniques for which catheter ablation of AF is a Class II recommendation (evidence for similarity of action between comparative technologies or techniques). AF, atrial fibrillation; CB, cryoballoon ablation; LVEF, left ventricle ejection fraction; PF, pulse field ablation; RF, radiofrequency ablation.

**Figure 3:**
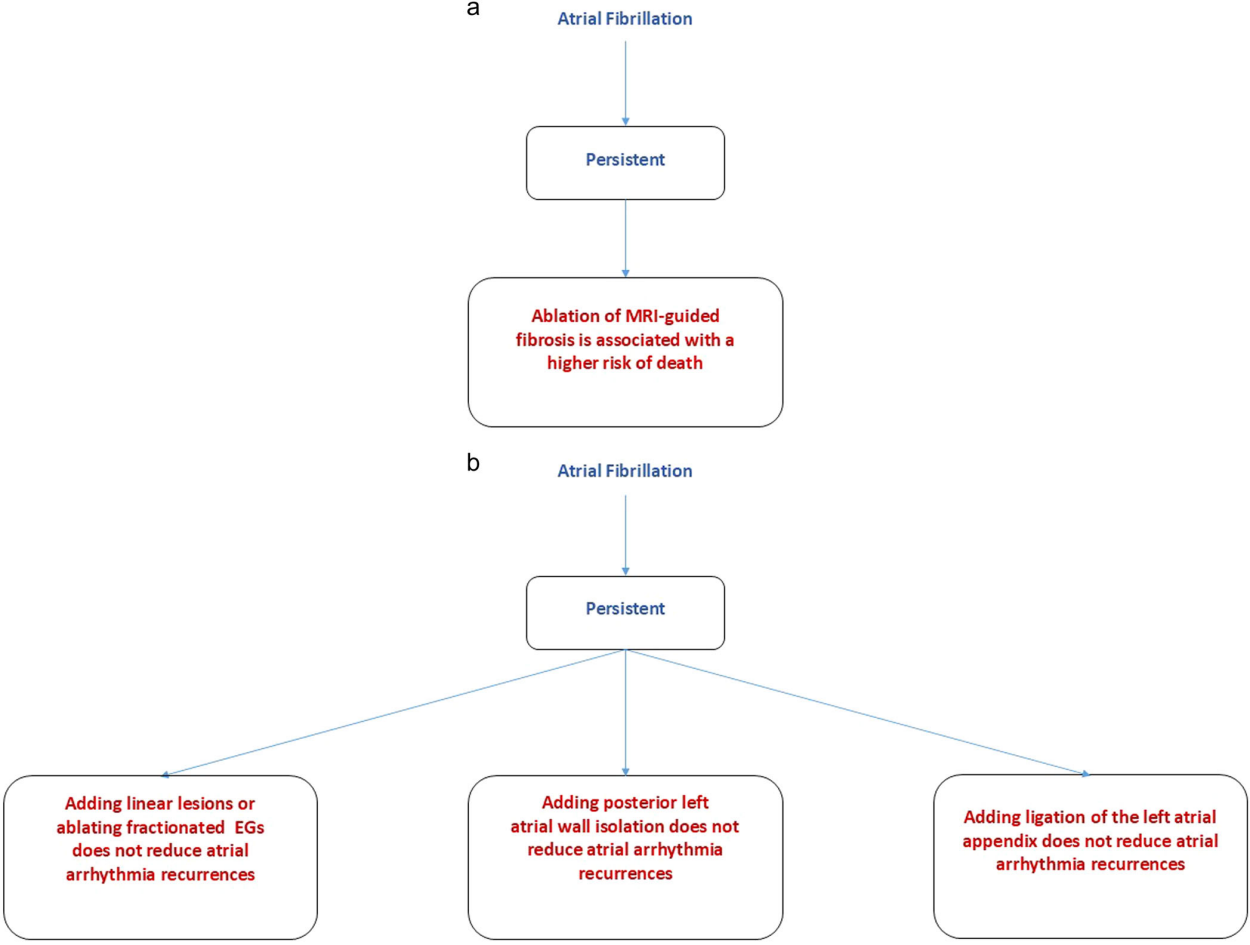
(a) Flowchart highlighting clinical conditions and catheter techniques for which catheter ablation of AF is a Class III recommendation based on evidence‐based documentation of harm. (b) Flowchart highlighting clinical conditions and catheter techniques in which catheter ablation of AF is a Class III recommendation based on evidence‐based documentation of lack of benefit with relevant numbers from reference studies. AF, atrial fibrillation; EGs, electrograms; MRI, magnetic resonance imaging.

Classes I–III recommendations are reported in Tables 1, 2, 3, respectively. Flowcharts showing clinical conditions for which catheter ablation of AF is indicated based on the proposed recommendation scheme are reported below in Figures 1, 2, 3, and 3b. Supporting Information: Figures S1–S3 provide complimentary information on clinical data including number of patients enrolled, randomization ratio and follow‐up duration in reference studies. Supporting Information: Figures S4, S5a, and S5b provide an alternative scheme of Supporting Information: Figures S1, S2a, S2b, S3a, and S3b, focusing primarily on techniques and technologies used to obtain designated outcomes. Supporting Information material provides the list of literature contributions representing the basis of our research (pages 24–54). Criteria for selection of recommendation classes in the present document are reported below.

Compilation of the present recommendation scheme is made under the assumption that future studies showing evidence against the current indications or new evidence from previously unaddressed indications will lead to appropriate changes in the new programmed edition of ECAS guidelines. This will apply especially for studies reporting outcome results from single high‐quality studies and for follow‐up extended beyond 12‐month duration, where applicable.

### 5.1 Criteria for Class I Recommendations

The criteria guiding selection of recommendation classes in the present document have been discussed elsewhere [10]. In brief, Class I indicates a recommendation in favor of the practice of catheter ablation or of any specific ablation strategy or technique, where:

a. evidence of greater benefit than risk or
b. similar efficacy and safety profiles between comparative technologies or techniques

is provided by high‐quality randomized multicenter controlled trials of sufficient sample sizes. High‐quality controlled trials guiding selection were defined as having:

a. low Risk of Bias for all crucial domains [71]
b. high precision of effect expressed by a narrow 95% confidence interval [72]
c. absence of other contradicting similar high‐quality evidence [72]
d. compliance with the principle of directness, comparability between the population investigated in the reference trial and the population to which the recommendation is directed [72].

Finally, multi‐center trials were required to ensure representativeness of proposed recommendations on large scale.

Failure to comply with these criteria resulted in a down‐grading of the ablation practice, strategy or technique to a recommendation Class II level (Table 1).

**Table 1:** Class I recommendations for catheter ablation.

Evidence for superiority of catheter ablation versus control therapy		
Recommendation	Class	Ref
‐ In **drug‐refractory paroxysmal AF**, *RF and CB PV isolation are superior to AADs* in reducing or suppressing atrial arrhythmia recurrences	I	[50, 51, 52]
‐ In **drug‐refractory persistent AF**, *RF PV isolation is superior to AADs* in reducing or suppressing atrial arrhythmia recurrences	I	[53]
‐ In **paroxysmal AF of first onset**, *CB PV isolation is superior to AADs* a in reducing or suppressing sustained atrial arrhythmia recurrences	I	[54, 55]
‐ In **paroxysmal AF**, *early (i.e. within 24 months from first documentation) CB PV isolation is superior to AADs* a in preventing progression to persistent AF	I	[56]
‐ In **paroxysmal and persistent AF and CHF with reduced EF**, *RF PV isolation is superior to AADs* a in reducing long‐term mortality and re‐hospitalization	I	[57]
‐ In **paroxysmal and persistent AF**, *PV isolation is superior to AADs* a in improving Quality of Life	I	[58]

**Evidence for similarity of efficacy and safety profiles between comparative technologies or techniques**		
**Recommendation**	**Class**	**Ref**
‐ In **drug‐refractory paroxysmal AF**, *RF and CB PV isolation* are similarly effective in preventing atrial arrhythmia recurrences	I	[27]
‐ In **drug‐refractory paroxysmal AF**, *RF and 2‐min or 4‐min Cryo‐ballon PV isolation* are similarly effective in reducing atrial arrhythmia recurrences and AF burden	I	[59]

a AADs include Classes I and III Vaughan‐Williams channel blockers.

### 5.2 Criteria for Class II Recommendations

Class II indicates a recommendation in favor of the practice of catheter ablation or of any specific ablation strategy or technique, where

a. evidence of greater benefit than risk or
b. similar efficacy and safety profiles between comparative technologies or techniques

is provided by

1. randomized clinical trials with one or more of:

a. no pre‐determined working hypothesis;
b. insufficient sample size;
c. well‐designed randomized clinical trials that were prematurely terminated because of futility or failure to enroll the anticipated population;
d. targeted meta‐analyses on qualifying endpoints for which high‐quality information is still not available.

Supporting Information: Table S2 provides the justifications adopted to qualify selected clinical or technical/technological conditions as Class II recommendations for efficacy in the present document.

Supporting Information: Tables S3–S5 indicate the characteristics and outcomes of clinical trials selected for custom‐made meta‐analyses relative to the specific indication addressed in these recommendations (Table 2).

**Table 2:** Class II recommendations for catheter ablation.

Evidence for superiority of catheter ablation versus control therapies or technologies/techniques		
Recommendation	Class	Ref
‐ In **paroxysmal AF**, *early (i.e., within 24 months from onset) RF PV isolation is superior to AADs* a in preventing progression to persistent AF	**II**	[60]
‐ In **paroxysmal AF of first onset**, *RF PV isolation is superior to AADs* a in reducing or suppressing atrial arrhythmia recurrences	**II**	[61]
‐ In **RF ablation of drug‐refractory persistent AF**, *adding vein of Marshall ethanol infusion is superior to PV isolation only* in reducing atrial arrhythmia recurrences	**II**	[62]
‐ In **symptomatic paroxysmal and persistent AF and end‐stage heart failure with reduced EF**, *RF PV isolation is superior to guideline‐directed medical therapy* in reducing all‐cause death, implantation of a left ventricular assist device or urgent heart transplantation	**II**	[63]
‐ In **drug‐refractory paroxysmal and persistent AF**, *PV isolation is superior to AADs* a in improving psychological symptoms of anxiety and depression	**II**	[64]
‐ In **drug‐refractory persistent AF with documented low‐voltage atrial areas**, *adding ablation of low voltage areas is superior to PV isolation only* in reducing or suppressing atrial arrhythmia recurrences	**II**	[65]
‐ In **drug‐refractory paroxysmal and persistent AF**, *PV isolation is superior to AADs* a in reducing the risk of future stroke§	**II**	[66]
‐ In **paroxysmal and persistent AF with or without previously failed AADs**, *PV isolation is superior to AADs* a to	**II**	[67]
o reduce AF burden		
o reduce all‐cause hospitalization		
o improve LVEF^b^		
‐ In **drug‐refractory paroxysmal AF**, *CB ablation is superior to RF ablation* in reducing re‐hospitalization	**II**	[68]

**Evidence for similarity of efficacy and safety profiles between comparative technologies or techniques**		
**Recommendation**	**Class**	**Ref**
‐ In **drug‐refractory paroxysmal AF**, *RF, CB and PF PV isolation* are similarly effective in preventing atrial arrhythmia recurrences	**II**	[69]
‐ In **drug‐refractory persistent AF**, *RF and dual‐energy PF and RF PV isolation* are similarly effective in reducing acute procedural failure, atrial arrhythmia recurrences, drug initiation or escalation or cardioversion	**II**	[70]

^a^ AADs include Classes I and III Vaughan‐Williams channel blockers.

^b^ Use of LVEF may be misleading in patients with AF, as the fast rate and the irregularity of the heart beat may lead to an arrhythmia‐dependent decompensation (“secondary CHF”); a better way to assess the benefit of AF ablation in this condition would include the following: (1) assessment of cardiac index; (2) assessment of LVEF after CV and a sufficient time interval in sinus rhythm allowing restoration of normal LVEF in patients with secondary CHF; these measures would allow to separate out patients with primary from patients with secondary CHF; randomized comparisons between AF ablation and group treatment in these two groups would be greatly welcome.

### 5.3 Criteria for Class III Recommendations

Class III indicates recommendations for which the practice of catheter ablation or of any specific ablation strategy or technique has shown evidence of harm overweighting benefit or has failed to show benefit (Table 3), as derived from high‐quality multicenter randomized clinical trials adopting pre‐determined working hypotheses and sufficient sample sizes (Figures 3a,b).

**Table 3:** Class III recommendations for catheter ablation.

Evidence for harm		
Recommendation	Class	Ref
‐ In **persistent AF**, *ablation of MRI‐guided fibrosis* is associated with a higher risk of death	**III**	[73]

Evidence against benefit from complimentary ablation strategies		
Recommendation	Class	Ref
‐ In **RF ablation of persistent AF**, *adding linear lesions or ablating fractionated EGs to PV isolation* does not reduce sustained atrial arrhythmia recurrences^a^	**III**	[74]
‐ In **RF ablation of persistent AF**, *adding posterior left atrial wall isolationto PV isolation* does not reduce sustained atrial arrhythmia recurrences^a^	**III**	[75]
‐ In **RF ablation of persistent AF**, *adding ligation of the left atrial appendix to PV isolation* by means of an epicardial clip system does not reduce sustained atrial arrhythmia recurrences	**III**	[76]

^a^ Empiric deployment at various sites in the left (and right heart) (see Chapter 3 for rationale and outcomes) and isolation of LAA are common; based on the lack of evidence at present, such practices are strongly discouraged as they may cause harm; the setting of randomized studies investigating a reasonable new working hypothesis is encouraged (see paragraph “Linear lesion, CFAE and ganglionated plexi, extra‐PV trigger and LAA isolation in ongoing clinical trials” below).

A comparative scheme of recently published recommendation schemes on catheter ablation of AF from the ESC, and the ACC/AHA/ACCP/HRS guidelines, and from the EHRA/HRS/APHRS/LAHRS consensus document with the present ECAS guidelines is reported in Supporting Information: Table S6.

## 6 Linear Lesion, CFAE and Ganglionated Plexi, Extra‐PV Trigger, Rotors and LAA Isolation in Ongoing Clinical Trials

There is a large body of studies investigating the role of supplementary linear lesion, CFAE or ganglionated plexi ablation. In patients with persistent AF, adding linear lesions [74], ablating fractionated atrial electrograms [39], adding posterior left atrial wall isolation [75] and ligation of the left atrial appendix [76] do not reduce atrial arrhythmia recurrences. Thus, at present, the available evidence does not fulfill our current Classes I and II recommendation requirements for using these supplementary ablation strategies in a generic, one size fits all approaches.

The therapeutic rationale behind these strategies and the possible reasons for their inability to improve clinical outcome in addition to PV isolation have been discussed in **Chapter 3**.

### 6.1 Linear Lesion Ablation

Overall, 10 RCTs published between 2005 and 2017 were selected (Supporting Information: Table S3) [74, 77, 78, 79, 80, 81, 82, 83, 84, 85]. Five of them [77, 78, 79, 80, 81] enrolling an aggregate of 643 patients with paroxysmal and non‐paroxysmal AF in the years between 2005 and 2008, compared supplementary left atrial linear ablation, including roof line and/or mitral isthmus line ablation, with PV isolation using ostial or segmental ablation (early technique) only. In these trials, supplementary linear lesion ablation showed superior clinical benefit as compared to PV ostial or segmental ablation.

Conversely, in the remaining 5 trials [74, 82, 83, 84, 85] conducted between 2011 and 2017 and enrolling an aggregate of more than 1000 patients, of whom the majority with non‐paroxysmal AF, supplementary linear lesion ablation failed to show superior clinical benefit as compared to PV ablation only. Of note, PV ablation in these latter trials was performed using WACA. All studies failed to report accurate data on linear lesion‐induced compartmentalization or integrity. Procedure duration and radiation exposure were significantly longer in patients receiving supplementary lesion ablation, whereas complication rates did not differ between the two groups. A custom‐made meta‐analysis performed by a selected group of authors to assure the quality of the guidelines has further confirmed the lack of clinical benefit by supplementary (but generic or non‐selective) linear lesion ablation as compared to PV isolation strategy only (Supporting Information: Figure S6).

### 6.2 CFAE Ablation

Overall, 8 RCTs published between 2004 and 2015 and enrolling 1312 patients with non‐paroxysmal AF in aggregate were selected [74, 86, 87, 88, 89, 90, 91, 92] (Supporting Information: Table S8). Of them, 3 trials reported a favorable outcome in patients receiving supplementary CFAE ablation versus patients receiving PV isolation only, whereas the other trials did not. In another trial [91], a higher incidence of recurrent atrial tachycardia or flutter was reported in the patient group receiving supplementary CFAE ablation whereas no differences between the two groups were found with respect to other complications. Most trials reported the use of automated software‐guided identification of target site for ablation (i.e., dv/dt based intrinsic deflection mean cycle length criterion of < 120 ms over 10 s), to replace the original manual process. These approaches fail to integrate other less well defined electrogram attributes such as visual averaging over time and relationships with surrounding electrograms. Other poorly defined parameters included EG source, type of catheter, electrode orientation and contact force. A meta‐analysis in 1415 patients with paroxysmal and persistent AF confirms the absence of benefit from supplemental (generic or non‐selective and variably defined) CFAE ablation as compared to PV isolation strategy only [93].

### 6.3 GP Ablation

Overall, 4 studies enrolling almost 1000 pts published between 2009 and 2022 have been selected out of a large body of literature in the field [94, 95, 96, 97] (Supporting Information: Table S5.

In a seminal RCT including 242 patients with paroxysmal AF, freedom from atrial arrhythmia recurrences in the PVI group was found to be significantly lower than the PVI + GPA group [94]. The same authors conducted a RCT in 264 patients with persistent/long‐lasting AF undergoing PVI who were randomized to additional linear LA ablation or GPA, all implanted with ILR. During the 3‐year follow‐up, freedom from atrial arrhythmias was lower in the linear ablation group with more atrial flutter recurrences [95].

However, the role of additional GPA has not been replicated by other centers, though some observational retrospective studies suggested beneficial effects in paroxysmal AF patients [98].

In another study selective ablation of the triggering‐GP was not superior to PVI alone, but the occurrence of pericarditis symptoms requiring hospitalization was higher following GPA than PV isolation [96].

Surgical GPA showed no benefit of additional GPA while increasing the associated risks of major bleeding and pacemaker implantation due to sinus node dysfunction [97, 99].

Several limitations of GPA and the interpretation of its results should be addressed. There are no uniform criteria for localizing ganglionated plexi (GPs), and their anatomical variability can lead to less effective ablation targets when using high‐frequency stimulation. The intraprocedural endpoint for GPA is less robust and standardized compared to PVI. GPs within epicardial fat pads are less susceptible to RF ablation, and some GPs located in the transverse sinus and between the superior vena cava and aortic root are inaccessible for the endocardium.

Endocardial GPA often overlaps with other AF targets like CFAE, rotors, and PV connections, confounding the assessment of its “pure” effects on clinical outcomes [42, 74, 94, 100]. Re‐innervation over time may reduce long‐term efficacy [100], and there are concerns about the safety of modulating autonomic cardiac function. Additionally, evidence comparing endocardial GPA (alone or with PV isolation) to standard AF ablation is limited and outdated, with most RCTs conducted by the same researchers about a decade ago, raising questions about the reproducibility and relevance of these findings [42, 74, 94, 100].

### 6.4 Extra‐PV Triggers Ablation

At present, there are no data from randomized controlled studies supporting the possible benefit of supplementary extra‐PV trigger ablation for the treatment of AF [29, 31]. The rationale for this strategy and its potential for improving clinical outcome when added to PV isolation have been discussed in **Chapter 3**.

### 6.5 Rotor Ablation

In 2011, the CONFIRM trial showed that rotors and focal sources were present in nearly all patients with paroxysmal and persistent AF, and that ablation of these areas nearly doubled the single‐ablation freedom from AF at 1 year [101]. More recently, it was shown that such benefit persisted at 3‐year follow‐up [102]. While some studies have confirmed these outcomes [103], others have not [104, 105]. Conclusive evidence supporting the ultimate benefit of this ablation strategy strongly awaits RCTs.

### 6.6 Implications for Guideline Recommendations

Based on evidence for no benefit, we elected to provide Class III recommendations for any of the strategies above in our current scheme. This consideration also applies to left posterior wall ablation and LAA isolation. Ongoing clinical trials (Supporting Information: S2) conducted with new technologies and innovative, personalized ablation strategies will help to refine our knowledge and possibly integrate these ablation techniques in more conclusive recommendation schemes in the next guidelines.

## 7 Efficacy Outcomes of Catheter Ablation of AF

Despite the large number of studies conducted, the true efficacy of AF ablation is difficult to estimate. An important limitation in this respect is represented by the definition used to assess post‐procedural recurrences, as several confounders may considerably affect reliable assessment of outcome measures. They include the definition of recurrent AF, duration of single episodes, presence of asymptomatic episodes, AF burden and methods used for documenting recurrent episodes. The variable combination of criteria used to assess efficacy is well reflected in literature and precludes rigorous comparability of outcomes among studies. A further confounder in efficacy assessment is represented by the presence of new arrhythmias whose substrate is determined by the scar lesions generated during catheter ablation. Finally, post‐procedural efficacy can be obtained with no need for AADs in some patients while others require chronic administration of previously ineffective AADs. Consistent with these limitations, efficacy rates of AF ablation have been reported to range between 52% and 83% in patient with paroxysmal AF, and between 37% and 77% in patients with persistent AF (Supporting Information: Table S10).

Growing awareness about the limitations of assessing the efficacy of catheter ablation of AF has prompted scientific societies to introduce specific recommendations on the methods and tools that best recognize and quantify post‐procedural atrial arrhythmia recurrences. Adoption of these recommendations has contributed to improving accuracy of outcome measures in recent years.

A method to obviate the current limitations is providing comparative assessment of post‐ablation outcomes between different strategies, such as in the case of catheter ablation versus AADs or control [50, 51] or one catheter ablation energy form versus another one [68, 69]. In such cases, adoption of prospective models combined with pre‐determined definitions of outcome obtained with rigorous diagnostic tools provides reliable estimate of comparative outcomes which, in turn, can be effectively transferred to clinical practice.

Given the limitations above, we present a tabulation on outcome efficacy data on paroxysmal AF (Supporting Information: Table S10a), persistent AF (Supporting Information: Table S10b and combined paroxysmal and persistent AF (Supporting Information: Table S10c) published in prospective studies enrolling at least 100 patients together with type of AF, role of AADs and methods used to assess atrial arrhythmia recurrences. Similarly, outcome safety data reported in these same studies on paroxysmal AF, persistent AF and combined paroxysmal and persistent AF are presented in Supporting Information: Tables S11a, S11b, and S11c, respectively. Data in these tables offer a comprehensive view on the range of efficacy and safety of catheter ablation of AF in different series and gives information on the conditions and limitations that might be expected when patients are referred for this procedure. They will also help investigators when introducing or assessing ablation programs in their institutions.

## 8 Periprocedural Anticoagulation

Periprocedural anticoagulation is meant to limit the risk of periprocedural thromboembolism [106] and comprises three management phases: (1) antithrombotic treatment before the ablation session; (2) intraprocedural anticoagulation; and (3) post‐procedural anticoagulation. Anticoagulation strategies and regimens need to be selected considering that even within therapeutic range, they may cause or worsen periprocedural bleeding. Bleeding is mostly observed at the site of vascular access, within the cardiovascular system (because of catheter‐induced cardiac or vessel perforation) and at peripheral sites, including intracranial, ocular, retroperitoneal [107].

### 8.1 Pre‐Ablation Anticoagulation

Most data relative to peri‐procedural anticoagulation is derived from studies of intermediate‐ to low‐quality. This is reflected in the recommendation scheme of the present guidelines, where the available evidence does not support anything more than a Class II indication.

Aside from patients with a low CHADS2, CHA2DS2‐VASc or CHA2DS2VA score and no pre‐existing antithrombotic treatment, most patients undergo catheter ablation under chronic anticoagulation. In comparison with an elevated risk of bleeding while bridging interrupted warfarin with subcutaneous heparin, use of uninterrupted warfarin was shown to provide a better safety profile [109]. Subsequent studies showed that uninterrupted or minimally interrupted (i.e., dose reduction or discontinuation on the procedure day for once‐daily prescriptions, or pre‐procedural discontinuation on the day of ablation) NOACs were as effective as VKAs, and safer with respect to major bleeding [108, 109, 110]. Therefore, uninterrupted or minimally interrupted NOAC treatment represents the currently preferred option. Table 5 shows details and a class of recommendation for peri‐procedural hemostasis and vascular access.

### 8.2 Intraprocedural Anticoagulation

Irrespective of the strategy used, intravenous heparin should be administered before or immediately following transseptal puncture [120]. Heparin dosing should be adjusted to achieve and maintain an ACT of at least 300 s throughout the ablation procedure duration (Table 4).

### 8.3 Post‐Procedural Anticoagulation

#### 8.3.1 Immediate Post‐Ablation Period

Patients under minimally interrupted NOACs should receive their next drug dose after procedure termination while uninterrupted dosing should continue as per prescription. Conversely, patients under uninterrupted VKAs should continue therapy under INR‐guided drug dose administration.

#### 8.3.2 Mid‐Long Term Post‐Ablation Period

Patients with low CHADS2, CHA2DS2‐VASc or CHA2D2VA score and with no mandatory indication for long‐term oral anticoagulation should discontinue anticoagulation therapy 2–3 months after ablation irrespective of clinical outcome as the risk of major bleeding due to therapy continuation in these patients outweighs the risk reduction of thromboembolism [121].

In patients with a CHADS2, CHA2DS2‐VASc or CHA2D2VA score of 2–3 and no documented atrial arrhythmia recurrences during 6‐month follow‐up, permanent discontinuation of oral anticoagulants post‐ablation may be safe [122].

**Figure 4:**
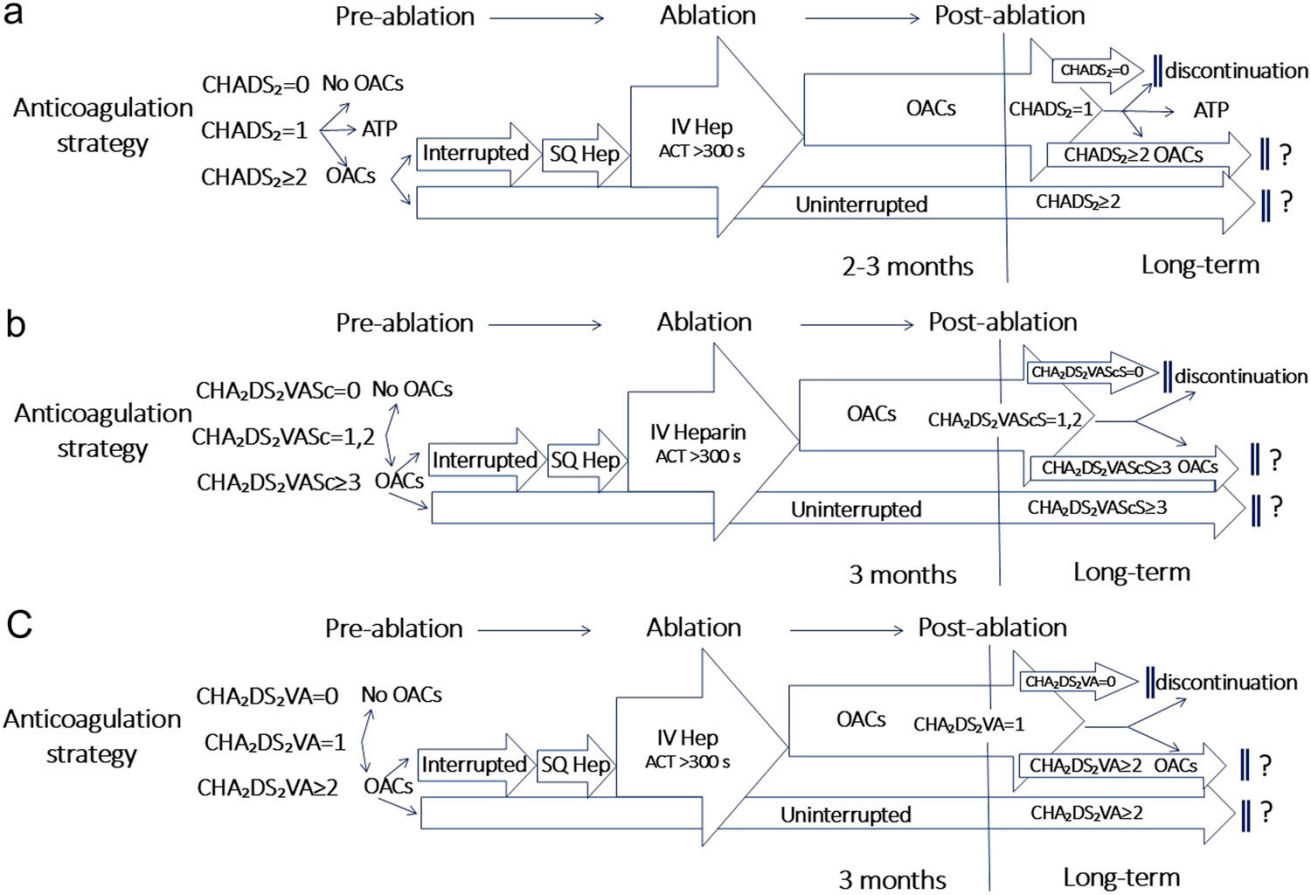
(a) Flowchart highlighting peri‐procedural anticoagulation based on CHADS2 score in patients undergoing AF ablation. (b) Flowchart highlighting peri‐procedural anticoagulation based on CH2ADS2‐VASc score in patients undergoing AF ablation. (c) Flowchart highlighting peri‐procedural anticoagulation based on CHA2DS2VA score in patients undergoing AF ablation. ACT, activated clotting time; Hep, heparin; IV, intravenous; OAC, oral anticoagulants; SQ, subcutaneous. Question marks indicate unsolved issues with respect to the possibility of discontinuing anticoagulation therapy post‐ablation in patients with no documented recurrences of sustained atrial arrhythmias.

To summarize the clinically most relevant situations of patients undergoing AF catheter ablation with a NOAC pre‐treatment or no pre‐treatment but initiation of a post‐ablation oral anticoagulation Figure 4 depicts a clinical pathway for anticoagulation handling depending on adoption of CHADS2 (a), CHA2DS2VASc (b), and CHA2DS2VA (c) individual risk score (Table 4 and 5).

**Table 4:** Recommendations on peri‐procedural anticoagulation.

Evidence for superiority of one anticoagulation versus another anticoagulation strategy of technique		
Recommendation	Class	Ref
− **Uninterrupted NOACs** are associated with *a lower risk of major bleeding* when compared with **uninterrupted VKAs** while leading to a comparably low rate of clinical stroke	**II**	[111, 112, 113, 114, 115, 116, 117]
− **Minimally interrupted** (suspension of 1 or 2 administration doses) **dabigatran** is associated with *lower rate of bleeding* compared to **uninterrupted VKAs**, while leading to a comparably low rate of clinical stroke	**II**	[118]
− **Uninterrupted VKAs** may be associated with a *lower rate of stroke and bleeding* compared to **interrupted VKA with heparin bridging**	**II**	[107]
− **Intraprocedural target ACT levels ≥ 300 sec** *are associated with very low rates of bleeding and thromboembolicc events*	**II**	[107, 111, 112, 113, 114, 115, 116, 117, 118]
− **Minimal interruption of NOACs (i.e, skipping on dose of drug)** may be associated with an *increased rate of silent stroke post‐ablation*	**II**	[119]
**Evidence for similarity of action between comparative anticoagulation therapies or strategies**		
**Recommendation**	**Class**	**Ref**
− **Minimally interrupted** (suspension of 1 or 2 administration doses) **NOACs** and **uninterrupted NOACs** are associated with *comparable rates of bleeding and clinical stroke*	**II**	[123, 124, 125, 126, 127, 128]
− **Interruption of NOAC for ≥ 24h with heparin bridging** is associated with *similar risks of bleeding and clinical stroke* as **uninterrupted VKAs** or **uninterrupted NOACs**	**II**	[118, 126]
− **Permanent discontinuation of oral anticoagulants in patients with a CHA2DS2VASc score of 2 to 3and no documented atrial arrhythmia recurrences during 6‐month post ablation follow‐up** is associated *whith comparable risk of cerebral embolic events* as compared **with continued OAC**	**II**	[122]
− **Figure‐of‐eight** and **vascular closure systems** are associated with *comparable rates of major bleeding*	**II**	[129, 130]

**Table 5:** . Recommendations for peri‐procedural hemostasis and vascular access.

Evidence for superiority of one versus another strategy of technique for hemostasis or vascular access in ablation procedures		
Recommendation	Class	Ref
− **Figure‐of‐eight or vascular closure systems** *are superior* **to manual pressure** *for achieving haemostasis* at femoral access site	**II**	[129, 130]
− Using manual pressure vascular haemostasis, intravenous **protamine administration** *reduces time to haemostasis and deambulation*	**II**	[131]
− **Ultrasound‐guided venous access** is associated with *faster access time, higher rate of first pass success and less inadvertent arterial puncture* compared to an **anatomical approach**	**II**	[132]

## 9 Complications of AF Ablation

The acceptability of an interventional or surgical procedure depends on the balance between efficacy and safety and catheter ablation of AF is no exception to this rule.

The overall complication rate of catheter ablation of AF is estimated to be around between 5.1% and 7.5% [133, 134, 135, 136, 137] with the commonest complications being those related to vascular access, followed by manifestations of volume overload, the occurrence of pericardial effusion and tamponade, and cerebrovascular accidents or TIAs.

Other less frequent complications include lesions of the vagus or phrenic nerve and pulmonary vein stenosis, inflammation or infection.

Esophageal fistulas, including atrio‐esophageal fistulas are by far the most lethal complication but fortunately the rarest as well in current practice.

Recent retrospective data on large collective indicate that complications with PF ablation may be less frequent than with former energy ablation techniques [138]. This observation contrasts with the data obtained in the first randomized study prospectively reporting comparative safety outcomes in patients assigned to PF ablation versus RF or cryo‐ablation [69] and awaits confirmative evidence from more objective data as those generated for RF and cryo‐ablation [133, 134, 135, 136, 137] as well as from further studies comparing PF ablation with these former techniques.

Incidences for each complication in the following list refer to NIS data sets [133] and literature searches from RCTs on AF ablation [137].

The following description provides information that may be helpful to prevent, recognize and efficiently treat peri‐procedural complications of AF ablation.

### 9.1 Complications of Vascular Access

The multiple and/or large bore venous access(es) necessary for this ablation procedure combined with anatomic variability of the femoral arterial tree and venous trunks and strong anticoagulation all favor the occurrence of complications such as local hematomas, arterio‐venous fistulas, and even retroperitoneal bleeding/hematoma.

#### *Incidence*: 0.7%–1.3%

##### Mitigation

Ultrasound guided venipuncture, micro puncture technique. Uninterrupted OACs may be associated with lower risk of bleeding at site of vascular access.

### 9.2 Volume Overload

Routine use of open‐tip irrigated RF catheters combined with prolonged procedures under general anesthesia results in large quantities of perfused IV fluids. Underlying heart failure or ventricular dysfunction, recurrent or resistant or new arrhythmias as well as the effects of large area atrial ablation (edema, reduced natriuretic peptide secretion) lead to manifestations of volume overload in the first 48–72 h after the ablation procedure.

#### *Incidence*: Not available

##### Mitigation

New generation irrigated tip catheters, short duration higher RF power irrigated tip ablation; monitoring cardiac filling pressures and body weight with appropriate diuretic use.

##### Management

use diuretics and other anti‐heart failure agents in case volume overload leads to intra‐ or post‐procedural decompensation.

### 9.3 Pericardial Effusion or Tamponade

Intra‐procedural or same day detection of pericardial effusion likely represents a hemorrhagic rather than inflammatory etiology although secondary conversion of inflammatory to hemorrhagic effusion is also possible. Hemorrhagic effusion is likely the result of traumatic transmural atrial wall damage due to mechanical puncture from transseptal needles or in locations weakened by RF lesions, steam pops or underlying disease. Tamponade is a hemodynamic sequel of an effusion large enough to compromise ventricular filling and forward cardiac output.

Hemorrhagic effusion is likely the result of traumatic transmural atrial wall damage—due to mechanical force particularly locations weakened by RF lesions, steam pops or underlying disease. Extensive manipulation, particularly with rigid catheters or in association with long sheaths, long left atrial dwell times, long ablation times, high contact forces and high RF powers with or without audible pops are all thought to increase the risk of this complication. Stronger anticoagulation in the form of higher ACTs (350–450 s) and uninterrupted OAC do not appear to increase the risk nor make management more difficult.

#### *Incidence*: 0.6%–2.2%

##### Mitigation

Optical monitoring of left heart contour and contraction as delineated in the left anterior oblique fluoroscopy projection may serve as baseline reference. Significant increase of left heart contour and/or reduced contraction during repeat (every 10–15 min) fluoroscopy control using the same fluoroscopy projection may raise the suspicion of ongoing pericardial effusion. Confirmation of pericardial effusion by means of transthoracic echo provides the opportunity to start pericardiocentesis under stable hemodynamic conditions before deterioration secondary to cardia tamponade occurs. In centers using ICE, continuous monitoring allows real‐time identification of pericardial effusion well before evolution to pericardial tamponade with higher sensitivity than using fluoroscopy.

Guiding transeptal puncture with ultrasound and using RF needles and micro guide wires may be of value. Minimizing/optimizing contact force and RF power, avoiding pops, manipulating cautiously and where possible while monitoring contact force may also be of value to minimize the risk of tamponade.

##### Management

Immediate neutralization of heparin‐induced anticoagulation by intravenous protamine sulfate infusion at recommended dose (i.e., 1.0–1.5 mg per 100 USP units of heparin). Pericardiocentesis can be rapidly effective once tamponade is recognized. Early detection by routine post‐procedural echo evaluation or monitoring with intracardiac echo during the procedure is, therefore, critical for optimal management with favorable outcomes. While rarely required, on‐site heart surgery or arrangements for expedite patient transfer to an institute able to treat large cardiac perforation or vascular tears that are not rapidly resolved by pericardiocentesis is mandatory.

### 9.4 Cerebrovascular or Other Systemic Embolic Events

Systemic embolic complications are likely the result of foreign material (catheters, guide wires, long sheaths etc.) resulting in activation of the coagulation cascade, stasis related clot formation and tissue overheating related coagulum and even char formation. Air and gas emboli may also play a role. Gaseous emboli manifest typically only transient effects.

#### *Incidence*: 0.5%–1.8%

##### Mitigation

Open tip irrigation with optimal low contact force and RF powers to avoid overheating, optimal intraprocedural anticoagulation (ACT > 300 s) as well as periprocedural oral anticoagulation, routine transesophageal pre‐procedural screening, careful de‐airing and procedural hygiene with long sheaths and catheter exchanges. Pre‐flushing all sheaths with high concentration heparin [139] and running heparin infusions to maintain the ACT through the sheaths spending time in the left atrium may further help to reduce the peri‐procedural thrombo‐embolic risk.

##### Management

Mechanical thrombectomy represents an effective catheter‐based therapy to rapidly remove a clot from a brain artery [140]. Following CT scan or X‐ray identification of the blood‐clot location and intravenous alteplase administration, advancement of the catheter to the culprit site allows clot capture and removal via the stent retriever. When a facility for thrombectomy is not available, intravenous alteplase should be administered as the only therapy to dissolve the clot [141].

### 9.5 Phrenic Nerve Injury

The right phrenic nerve, coursing down to the right hemi‐diaphragm on the parietal pericardium is vulnerable to thermal (cryo‐energy or rarely RF) damage by a proximity effect during RSPV (or right sided SVC) ablation [142]. Balloon based single shot devices because of their distending effects are the commonest offenders but other single shot devices such as loop or basket catheters are also known to be implicated. Point RF ablation catheters have also rarely been implicated during RSPV isolation and perhaps more commonly during SVC isolation or ablation. Ablation in the right atrium may also damage the right phrenic nerve lower down in its trajectory towards the right hemi‐diaphragm.

The left phrenic nerve is usually well removed from the left superior PV and closer to the left atrial appendage, and therefore, not known to be vulnerable during PV isolation although LAA ablation as well as ablation within a persistent LSVC may endanger this nerve.

Most phrenic nerve injuries are transient and resolve with time, which may take up to several months in selected cases.

#### *Incidence*: 4.2%

##### Mitigation

Avoiding sites in proximity to the phrenic nerve as indicated by pace capture or ablating with lower energy intensities while monitoring right hemi‐diaphragmatic capture by stimulating the nerve upstream in the high SVC. Weakening contraction or loss of capture should prompt cessation of energy delivery. Pacing from a different catheter during RF energy delivery may help to monitor phrenic nerve reaction. If the phrenic nerve is stimulated, the diaphragm will be observed to contract and RF can be stopped immediately.

##### Management

No therapy is currently available to resolve chronic phrenic nerve injury.

### 9.6 ‘Vagal’ Nerve Lesions

This complication refers to lesions damaging nerve segments or components of a vagal plexus (rather than the vagal nerve trunk) responsible for innervating the stomach and the pyloric sphincter in the posterior mediastinum between the esophagus and the posterior left atrium. As a result of this lesion, typically gastric hypomotility and pyloric spasm are known to occur with both cryo‐balloon as well as RF ablation, and typically in proximity to the right inferior pulmonary vein ostium.

**Mitigation.** Because of the highly variable anatomy and lack of functional monitoring, no currently known specific mitigation or therapeutic measures apart from minimizing posterior LA wall ablations in terms of both extent and energy delivery.

### 9.7 Atrio‐Esophageal Fistula

Atrio‐esophageal fistula occurs a s a result of extra‐cardiac thermal injury to the esophagus and neighbouring mediastinum and represents one rarest, albeit most lethal, complications of catheter ablation of atrial fibrillation. An additional and subsequent inflammatory and/or ischemic phenomenon appears necessary for the typically delayed development of full‐fledged fistulous communication with the left atrium. The lethality of this complication is due to repeat air embolism to the brain occurring once the fistula has been formed. It is known to occur more frequently with RF ablation compared to cryo‐ablation but has not been reported yet with PF ablation which has a significantly higher sensitivity for myocardial versus esophageal tissue. A very high mortality rate of 60%–80% has been reported, attributable to both late diagnosis as well as the difficulty of repairing the breach, thus emphasizing the importance of prevention and early diagnosis [143, 144, 145].

#### *Incidence*: 0.01%–0.04%

##### Mitigation

Avoiding extra‐cardiac thermal injury to the posterior mediastinum in relation to the esophagus by reducing delivered RF energy and contact force. Of note, there is no reliable evidence of the benefit of esophageal temperature monitoring, cooling or retraction nor of administration of proton‐pump inhibitors. The rarity of this complication and our lack of understanding of the evolution of extra‐cardiac thermally mediated esophageal injury to fistula formation are both significant barriers to developing effective mitigation or avoidance measures. Awareness of this complication should orient when sign of its presence such as fever, neurological or gastrointestinal symptoms occur. Early diagnosis may be critical to provide early therapy.

##### Management

Early surgery is thought to be the only intervention that reduces mortality although the evidence is necessarily only anecdotal.

### 9.8 Peri‐Procedural Death

Peri‐procedural death occurs in 1 every 1000, and in 1 every 200 patients undergoing AF ablation depending on whether its incidence is assessed retrospectively [146] or prospectively [147, 148]. These figures reflect estimates on large scale and there is consistent evidence that death rates are dependent on volume of performed procedures and operator experience, with lower rates being reported in centers with higher volume and by operators with greater experience [133].

Death may occur during hospitalization (early death) or after discharge as a consequence related to the ablation procedure (late death). Death cases related to the ablation procedure have been reported well beyond 30 days from the procedure.

Among causes precipitating peri‐procedural death, the commonest is represented by cardiac tamponade (about 25%), with one in eight events being reported later than 30 days after procedure. Peri‐procedural stroke and atrio‐esophageal fistulae account for about 15% of fatal events each. Half of stroke‐related deaths occur after 30 days from procedure because of the serious consequences associated with the primary neurologic damage. Atrio‐esophageal fistulae typically manifest weeks after procedure and are fatal in more than 60% of cases. Other less common causes of peri‐procedural death include massive pneumonia, myocardial infarction, intractable torsade de pointes, septicemia, sudden respiratory arrest, extra pericardial PV perforation, occlusion of both PVs from lateral or septal region and anaphylaxis. Knowledge of possible precipitating causes is key to operators and needs to be considered during decision making with patients.

Supporting Information: Tables S11a, S11b, and S11c are providing a tabulation of safety data published in prospective studies enrolling at least 100 patients undergoing catheter ablation of paroxysmal and/or persistent AF. Data in this table offer a comprehensive view on the range of complications of catheter ablation of AF in different series. Similar to what addressed in Supporting Information: Tables S10a, S10b, and S10c for efficacy outcomes, review of safety data as proposed in this table may help to anticipate the challenges to be expected when referring patients for AF ablation and mitigate or treat their occurrence. This will also help investigators when introducing or reviewing ablation programs in their institutions.

## 10 Ongoing Trials

While the present document is published, dozens of RCTs are being conducted addressing clinically and technologically relevant items in the field of AF ablation. The results of these trials will contribute to improve our knowledge and guide future clinical activities.

With the aim of providing readers with detailed information about the ongoing research, we have incorporated a dedicated table reporting the list of ongoing RCTs currently registered on the clinicaltrials.gov (date of access) platform in Supporting Information S2. Overall, 233 RCTs are presently underway of which 21 will investigate the impact of catheter ablation on clinically relevant outcomes, 55 will investigate the impact of novel ablation catheters/technologies, 69 will investigate the efficacy of new catheter approaches or targets on clinical outcomes, 14 will investigate the benefit of complimentary drugs or other interventions to improve catheter ablation outcomes, 12 will investigate the role of novel mapping strategies on catheter ablation outcomes, 10 will investigate the benefit of novel anticoagulation strategies on peri‐procedural protection from thromboembolism and bleeding, 6 will investigate interventions aimed at reducing peri‐procedural complications, 4 will compare the benefit of catheter ablation versus sham control, and 2 will investigate prediction models for favorable outcome. There will be 16 more RCTs investigating the benefit of surgical ablation versus various comparative treatments, and 21 investigating other aspects of peri‐procedural care (i.e., sedation, etc).

Supporting Information: Tables S7–S9 is meant to provide readers with a comprehensive picture of current research and which pending clinical and technique/technology questions will likely be answered in the months and years to come.

## 11 Emerging Role of PF Energy for AF Ablation

As we write the present document, a large bulk of studies are being published or underway to investigate the role of pulsed field energy delivery, a new emerging technology for lesion deployment in the heart, for catheter ablation of AF. This energy form consists in the transmission of pulsed energy to the heart that determines electroporation of the cell membrane leading to irreversible tissue damage.

The claimed selectivity for cardiac tissue associated with the rapid effect (within second) after onset of energy release, has boosted great enthusiasm about the efficacy and safety potential of this technology for treating AF [149]. While we recognize the potential, the available data should still be considered preliminary and certainly not comparable in size with the multi‐decade experience of RF and Cryo‐ablation. In the first RCT of RF versus PF ablation, the two techniques showed similar efficacy in patients with paroxysmal AF and one fatality case was observed in the PF study group [69]. More recently, unexpected complications such as coronary artery spasm, renal insufficiency, hemolysis and cerebral thromboembolism have been documented during and after PF ablation of AF patients [150, 151, 152, 153]. Meanwhile, experimental studies have shown that early disappearance of electrical activity is transient unless obtained with high contact pressure at target ablation sites, a factor possibly affecting long‐term efficacy and peri‐procedural safety [154]. Most studies using this technique, conducted prospectively in patients with persistent AF are observational [155] and require rigorous comparison with control techniques before superiority of PF ablation can be established.

For these reasons, we have elected to adopt a prudent approach when including PF ablation in our recommendation scheme. Ongoing trials will contribute to refining the present scheme based on study results.

## 12 Heart Failure and AF Ablation

The clinical findings of heart failure or imaging evidence of impaired cardiac function occurring with AF can be a management dilemma. If the AF is assumed to be secondary to the heart failure, the initial practice would be the commencement of heart failure drugs and screening for secondary causes of heart failure. This will be followed by a determination of the need for device prophylaxis with an implantable defibrillator. However, AF‐induced cardiomyopathy (AFICM) is now well‐described, and the restoration of sinus rhythm may result in complete resolution of cardiac impairment. This form of cardiomyopathy may arise secondary to the tachycardia but is also seen in the presence of rate‐controlled AF. Functional mitral regurgitation of varying severity may also be part of a vicious cycle that exacerbates this type of cardiomyopathy. Failing to identify those patients with AFICM can compromise the optimal management of this important group of patients with heart failure.

The diagnostic and therapeutic challenge for the clinician is determining whether to focus treatment on the heart failure or the AF. If the clinical history is suspicious for AFICM, then restoration of sinus rhythm using a combination of AADs, DC‐cardioversion or AF ablation may help the need for heart failure treatment. In some patients, the restoration of sinus rhythm will not fully resolve the impairment of cardiac function but will markedly improve the symptoms and cardiac function. These patients will not be diagnosed as having AFICM, but cardiomyopathy that has been exacerbated by AF.

Several trials may help determine the optimal approach to managing this group patients. Early restoration of sinus rhythm also resulted in improved cardiovascular outcomes in the EAST‐AFNET study, but only a minority of these patients had concomitant heart failure [156]. Some of them have directly addressed the question of whether AF ablation to restore sinus rhythm in patients with heart failure is beneficial compared to optimal medical therapy alone. The CAMTAF trial [157] and the CAMERA‐MRI [158] trial both showed catheter ablation was superior to rate control for improving LV ejection fraction. The AATAC trial was able to show reduced mortality and hospitalization in the ablation group as a secondary outcome [159]. Further, confirmation of this finding came from the CASTLE‐AF trial which showed superiority of catheter ablation to medical therapy as the primary objective [57]. The more recent RAFT‐AF trial used the same composite endpoint of death and heart failure hospitalization and showed a trend favoring catheter ablation, but this did not reach significance [160]. The CASTLE‐AF trial patients had a median ejection fraction of 32% and all had a defibrillator in‐situ, whereas only about 25% of patients had a defibrillator in RAFT‐AF and 40% of patients had an ejection fraction > 45%. The left atrial diameters and proportion of patients with persistent AF were similar in both studies, implying that restoration of sinus rhythm is even more important in those with more severe LV dysfunction. This suggests that there are subgroups, within the AF with heart failure population, who may have greater benefit and the current trial data may not help identify these patients. For example, it is not known if a trial of DC cardioversion to identify patients whose LV function improves is a beneficial strategy or whether the mortality and hospitalization benefits are independent of such findings. Aggressive rate control with AV node ablation should also be considered as the APAF‐CRT trial suggested a resynchronization pacemaker followed by rate control by AV‐node ablation was more effective at reducing mortality than medical therapy alone [161]. It is not known if such a strategy is comparable to achieving sinus rhythm or should only be applied in those patients in whom sinus rhythm cannot be maintained.

While consistent evidence has been reported about the role of AF ablation in patients with CHF, we acknowledge missing evidence about the benefit that AF ablation may provide depending on CHF sub‐categories, such as for example primary CHF, CHF secondary to AF and intermediate groups, or HFrEF, HFmrEF and HFpEF [162]. With the aim of fulfilling this gap, we encourage research that will accurately distinguish sub‐categories of CHF and AF at the time of screening. To this purpose, indicators of heart performance such as cardiac index or LVEF assessment after pharmacological or electrical restoration of sinus rhythm would help to distinguish between CHF sub‐categories. Randomized comparison between AF ablation and drug treatment within each sub‐category would help filling a relevant knowledge gap in this discipline and identify sub‐groups of CHF patients obtaining better prognostic benefit from ablation.

## 13 Peri‐Procedural Mortality

Among procedure‐related complications, early mortality accounts for up of 0.5% of patients [133]. Accurate estimates of the true incidence of peri‐procedural mortality are difficult to obtain. The earliest documentation of its occurrence was reported about one decade after the introduction of this technique in clinical practice [163], and was based on a rather approximate, voluntary‐based contribution by centers contributing to a worldwide survey. At that time, the reported incidence of this complication was 0.5%. Since then, various studies have addressed this issue giving the perception that the incidence of peri‐procedural mortality was decreasing as investigator experience was growing. This is of great importance because of the increasing volume of procedures treating increasingly sicker patients with more complex substrates. However, the question remained whether the accuracy of data reported from single studies or multi‐center registries and surveys are representative of the true incidence of this complication in the real world.

A robust method for accurate assessment of peri‐procedural mortality was first introduced in 2013, when Deshmukh et al. [133] reported on a large survey of in‐hospital complications associated with 93 801 AF ablation procedures in the United States between 2000 and 2010. Data was obtained from NIS data set representing a nation‐based survey conducted by the Healthcare Cost and Utilization Project in collaboration with the participating states. ICD‐9‐CM codes were used to identify each of the study diagnoses investigated. Trends in complications showed that in‐hospitalization death occurred at a rate of 0.42% and that this figure tended to be stable throughout the investigated period [133]. Mortality rates were found to be higher in centers with lower patient volumes and less operator experience. Using a similar method (i.e., the United States Agency for Healthcare Research and Quality—AHRQ), Cheng et al. [147] reported an early mortality rate of 0.46% in 60 203 patients during the years 2000–2015 with 54% of deaths occurring during 30‐day readmission.

These figures reliably indicate the true incidence of peri‐procedural mortality of AF ablation and indicate that death may occur in 1:200 patients undergoing this procedure.

We, therefore, strongly advocate that:

− clear, honest and comprehensive information is given to patients about mortality risks at the time of therapy prescription;
− regular education and simulations/rehearsal (i.e., every 2 or 3 months) programs for all staff‐members enabling them to recognize early and treat acute clinical deterioration resulting from life‐threatening procedure‐related complications;
− when not available, arrangements should be in place for patient transfer to an institute able to treat rare complications like cardiac perforation or vascular tears.

## 14 Mortality Benefit of Catheter Ablation

Historical studies examining the mortality benefit for rhythm control of AF (vs. rate control) have primarily focused on the use of antiarrhythmic drugs [164, 165]. These studies have been relatively small and have either been neutral or have suggested that rhythm control is associated with greater mortality. The obvious ease of prescribing drug therapy is countered by the limited success, lack of precision of antiarrhythmic drugs, and their potential side effects, pro‐arrhythmia being the most concerning. Catheter ablation by contrast has been shown to be superior to drugs in achieving rhythm control but has a front‐loaded risk [166]. Several case cohort and population studies have sought to examine the impact of catheter ablation on mortality and have shown benefit. These studies however are limited by their size or design. To date only two large randomized controlled trials have been performed. CABANA compared catheter ablation to standard medical therapy in a large patient cohort [51]. The results were neutral if one examined the data as the trial was designed, intention to treat. The study outcomes were significantly limited by the one‐third of patients who crossed from the medical arm and received catheter ablation and only when performing a per treatment analysis was a statistically significant mortality benefit seen. The EAST trial examined the impact of early rhythm control (within 1 year of AF diagnosis) in patients with concomitant cardiovascular risk factors [156]. The study compared usual care (which limited rhythm control only for AF related symptoms) to early rhythm control. Rhythm control was initially predominantly antiarrhythmic drug therapy with catheter ablation performed in about one‐fifth of patients at the 2‐year follow up point. In this study early rhythm control did have mortality benefit but the study did not have sufficient data to examine the impact of catheter ablation which contributed to only 20% of adopted strategies to achieve rhythm control.

The impact of catheter ablation of AF associated with heart failure has been examined and several studies have shown that AF ablation is associated with a mortality benefit. CASTLE‐ AF is the largest randomized trial and showed a significant mortality benefit [57]. Although the results of this trial have been challenged because of the very high success rates for the AF ablation, the methodology was sound, and the results have been echoed in other studies and reviews [157, 158, 159, 166].

It is reasonable to conclude that while there are signals in the literature that catheter ablation may be associated with a reduced mortality, there are insufficient data to conclude that this should be recommended in the absence of AF symptoms other than in patients who have associated heart failure, in which case one might argue they do have symptoms, the symptoms from the heart failure if not directly from the AF.

One challenge in this area is that the patients who may be most likely to gain from successful catheter ablation, namely young patients with lone paroxysmal AF who are likely to have a good outcome from ablation and be exposed to many years of AF or antiarrhythmic drugs in its absence. These patients very unlikely to be included in mortality trials because of the very low event rate and long follow up period in large numbers of patients that will be required to have a chance of showing a significant difference. This problem was, until this decade, somewhat academic because patients with symptoms would have ablation for this reason and patients without symptoms would be unlikely to be diagnosed. However, the increasing use of wearable technologies that diagnose AF [167], mean that there is a growing population of patients who are faced with the dilemma of having highly treatable AF without symptoms and have to make a decision whether to have catheter ablation in the hope that this will have a prognostic impact without the support of robust data.

We would, therefore, encourage that:

− AF ablation is offered to patients with heart failure, particularly when there are no other causes for the heart failure and/or there is a temporal associated between the heart failure and the AF.
− AF ablation should be offered to patients with symptoms associated with AF, particularly if they are at high risk of other cardiovascular disease.
− Asymptomatic patients should be offered information about the possible mortality benefit of AF ablation but must be told about the absence any robust data supporting this conclusively.

If patients do decide to undergo an ablation, a full discussion should be given allowing them to understand the risks and success rates of the procedure in their specific case. Asymptomatic AF patients with a low probability of successful ablation, for example patients with persistent AF with severely dilated atria and no evidence of sinus rhythm in the last 1–3 years should probably be dissuaded from ablation given the lack of evidence supporting this approach.

## 15 Main Differences Between ECAS GLs and Equivalent Recently Published Documents on AF Ablation

The main differences between ECAS GLs and the most recently published documents in the field, including the 2024 ESC GLs [3], the 2023 ACC/AHA/HRS GLs [2] and the EHRA/HRS/APHRS/LAHRS consensus document [4], are reported in Supporting Information: Table S6. In brief, the classification scheme in the present GLs appears simpler than the one adopted by the other documents. This is justified by complete elimination of level or type of evidence for each recommendation class. With respect to specific indications, the more rigorous inclusion criteria adopted in the present GLs is reflected in the lower number of recommendations addressed as compared with the number of recommendations in the other documents. The difference within specific indications across the four GL and consensus documents is reflected by the statement “Cannot be classified” in the ECAS pertinent row of Supporting Information: Table S6. Adoption of more rigorous inclusion criteria and rejection of level or type of evidence sub‐classification within single classes in the ECAS GLs is also reflected in Supporting Information: Table S6 by the less populated justifications for most indications.

## 16 Summary and Future Directions

Adopting the growing request for rigor and transparency, the present document provides a concise scheme on Classes I–III recommendations relative to beneficial effects of catheter ablation of AF and of specific ablation strategies or techniques for which high‐quality evidence of greater benefit than risk is demonstrated. Consistent with the rigorous methods adopted, subclassifications and levels of evidence have been deleted with the aim of mitigating arbitrariness in document production. The copious ongoing research in the field, of which we have provided a custom‐built list for readers reference, will enrich our recommendation list in future guidelines, with the awareness that new paradigms may surface offering different base theories, methods of investigation and validation may subvert current guidelines' structure. The model used here is meant to preserve the original mission of guidelines to assist, not to impose practitioner decisions about appropriate health care and reconcile them with the true art of medicine. The rigor, simplicity and transparency of the present document may serve other societies in preparation guideline documents.

## Supporting information

Supporting information.

Supporting information.

## Data Availability

The data that supports the findings of this study are available in the supplementary material of this article.

## References

[1] Institute of Medicine ., “(US) Committee to Advise the Public Health Service on Clinical Practice Guidelines.” in Clinical Practice Guidelines: Directions for a New Program, eds. M. J. Field and K. N. Lohr ((US): National Academies Press, 1990. Accessed: August 23, 2024, http://www.ncbi.nlm.nih.gov/books/NBK235751/. 25144032

[2] J. A. Joglar , M. K. Chung , A. L. Armbruster , et al., “2023 ACC/AHA/ACCP/HRS Guideline for the Diagnosis and Management of Atrial Fibrillation: A Report of the American College of Cardiology/American Heart Association Joint Committee on Clinical Practice Guidelines,” Circulation 149, no. 1 (2024): e1–e156, 10.1161/CIR.0000000000001193. 38033089 PMC11095842

[3] I. C. Van Gelder , M. Rienstra , K. V. Bunting , et al., “2024 ESC Guidelines for the Management of Atrial Fibrillation Developed in Collaboration With the European Association for Cardio‐Thoracic Surgery (EACTS),” European Heart Journal 45, no. 36 (2024): 3314–3414, 10.1093/eurheartj/ehae176. 39210723

[4] S. Tzeis , E. P. Gerstenfeld , J. Kalman , et al., “2024 European Heart Rhythm Association/Heart Rhythm Society/Asia Pacific Heart Rhythm Society/Latin American Heart Rhythm Society Expert Consensus Statement on Catheter and Surgical Ablation of Atrial Fibrillation,” Europace: European Pacing, Arrhythmias, and Cardiac Electrophysiology: Journal of the Working Groups on Cardiac Pacing, Arrhythmias, and Cardiac Cellular Electrophysiology of the European Society of Cardiology 26, no. 4 (2024): euae043, 10.1093/europace/euae043. 38587017 PMC11000153

[5] J. S. Burgers , B. Fervers , M. Haugh , et al., “International Assessment of the Quality of Clinical Practice Guidelines in Oncology Using the Appraisal of Guidelines and Research and Evaluation Instrument,” Journal of Clinical Oncology 22, no. 10 (2004): 2000–2007, 10.1200/JCO.2004.06.157. 15143093

[6] M. C. Brouwers , M. E. Kho , G. P. Browman , et al., “Agree II: Advancing Guideline Development, Reporting and Evaluation in Health Care,” Canadian Medical Association Journal 182, no. 18 (2010): E839–E842, 10.1503/cmaj.090449. 20603348 PMC3001530

[7] T. M. Shaneyfelt , M. F. Mayo‐Smith , and J. Rothwangl , “Are Guidelines Following Guidelines? The Methodological Quality of Clinical Practice Guidelines in the Peer‐Reviewed Medical Literature,” Journal of the American Medical Association 281, no. 20 (1999): 1900–1905, 10.1001/jama.281.20.1900. 10349893

[8] R. Grilli , N. Magrini , A. Penna , G. Mura , and A. Liberati , “Practice Guidelines Developed by Specialty Societies: The Need for a Critical Appraisal,” The Lancet 355, no. 9198 (2000): 103–106, 10.1016/S0140-6736(99)02171-6. 10675167

[9] H. Calkins , G. Hindricks , R. Cappato , et al., “2017 HRS/EHRA/ECAS/APHRS/SOLAECE Expert Consensus Statement on Catheter and Surgical Ablation of Atrial Fibrillation,” Heart Rhythm: The Official Journal of the Heart Rhythm Society 14, no. 10 (2017): e275–e444, 10.1016/j.hrthm.2017.05.012. PMC6019327 28506916

[10] R. Cappato , S. Levy , R. Providencia , et al., “Concise Guidelines of the European Cardiac Arrhythmias Society (ECAS) on “Catheter Ablation of Atrial Fibrillation”: A Prepublication of the Methods in Preparation of the Final Guidelines Document,” Journal of Cardiovascular Electrophysiology 35, no. 7 (2024): 1490–1494, 10.1111/jce.16254. 38736156

[11] E. Severino , Nothingness and Poetry. *At the End of Technological Age: Leopardi* (Rizzoli, 1990).

[12] O. Besomi and M. Helbing *Galileo Galilei. Dialogo Sopra i Due Massimi Sistemi Del Mondo Tolemaico e Copernicano, (Medioevo e Umanesimo102/103)*. Antenore; 1998.

[13] G. Gillett , “Medical Science, Culture, and Truth,” Philosophy, Ethics, and Humanities in Medicine 1 (2006): 13, 10.1186/1747-5341-1-13. PMC1769504 17178003

[14] Online Etymology Dictionary .

[15] M. Haïssaguerre , D. C. Shah , P. Jaïs , et al., “Mapping‐Guided Ablation of Pulmonary Veins to Cure Atrial Fibrillation,” American Journal of Cardiology 86, no. 9A (2000): 9K–19K, 10.1016/s0002-9149(00)01186-3. 11084094

[16] M. Haïssaguerre , P. Jaïs , D. C. Shah , et al., “Spontaneous Initiation of Atrial Fibrillation By Ectopic Beats Originating in the Pulmonary Veins,” New England Journal of Medicine 339, no. 10 (1998): 659–666, 10.1056/NEJM199809033391003. 9725923

[17] F. Ouyang , R. Tilz , J. Chun , et al., “Long‐Term Results of Catheter Ablation in Paroxysmal Atrial Fibrillation: Lessons From a 5‐year Follow‐Up,” Circulation 122, no. 23 (2010): 2368–2377, 10.1161/CIRCULATIONAHA.110.946806. 21098450

[18] B. P. Knight , P. G. Novak , R. Sangrigoli , et al., “Long‐Term Outcomes After Ablation for Paroxysmal Atrial Fibrillation Using the Second‐Generation Cryoballoon,” JACC: Clinical Electrophysiology 5, no. 3 (2019): 306–314, 10.1016/j.jacep.2018.11.006. 30898232

[19] C. H. Heeger , S. S. Popescu , T. Inderhees , et al., “Novel or Established Cryoballoon Ablation System for Pulmonary Vein Isolation: The Prospective ICE‐AGE‐1 Study,” Europace: European Pacing, Arrhythmias, and Cardiac Electrophysiology: Journal of the Working Groups on Cardiac Pacing, Arrhythmias, and Cardiac Cellular Electrophysiology of the European Society of Cardiology 25, no. 9 (2023): euad248, 10.1093/europace/euad248. 37589146 PMC10468200

[20] C. H. Heeger , C. M. Tiemeyer , H. L. Phan , et al., “Rapid Pulmonary Vein Isolation Utilizing the Third‐Generation Laserballoon ‐ The Phoenix Registry,” International Journal of Cardiology. Heart & Vasculature 29 (2020): 100576, 10.1016/j.ijcha.2020.100576. 32642555 PMC7334810

[21] S. R. Dukkipati , K. H. Kuck , P. Neuzil , et al., “Pulmonary Vein Isolation Using a Visually Guided Laser Balloon Catheter: the First 200‐patient Multicenter Clinical Experience,” Circulation: Arrhythmia and Electrophysiology 6, no. 3 (2013): 467–472, 10.1161/CIRCEP.113.000431. 23559674

[22] Y. Wakamatsu , S. Nakahara , K. Nagashima , et al., “Hot Balloon Versus Cryoballoon Ablation for Persistent Atrial Fibrillation: Lesion Area, Efficacy, and Safety,” Journal of Cardiovascular Electrophysiology 31, no. 9 (2020): 2310–2318, 10.1111/jce.14646. 32613693

[23] A. Verma , D. E. Haines , L. V. Boersma , et al., “Pulsed Field Ablation for the Treatment of Atrial Fibrillation: PULSED AF Pivotal Trial,” Circulation 147, no. 19 (2023): 1422–1432, 10.1161/CIRCULATIONAHA.123.063988. 36877118 PMC10158608

[24] L. Urbanek , S. Bordignon , D. Schaack , et al., “Pulsed Field Versus Cryoballoon Pulmonary Vein Isolation for Atrial Fibrillation: Efficacy, Safety, and Long‐Term Follow‐Up in a 400‐Patient Cohort,” Circulation: Arrhythmia and Electrophysiology 16, no. 7 (2023): 389–398, 10.1161/CIRCEP.123.011920. 37254781

[25] C. Jilek and W. Ullah , “Pulmonary Vein Reconnections or Substrate in the Left Atrium: What Is the Reason for Atrial Fibrillation Recurrences? A Dialogue on a Pressing Clinical Situation,” EP Europace 21, no. Suppl_1 (2019): i12–i20, 10.1093/europace/euy289. 30801129

[26] M. S. Brahier , D. J. Friedman , T. D. Bahnson , and J. P. Piccini , “Repeat Catheter Ablation for Atrial Fibrillation,” Heart Rhythm: The Official Journal of the Heart Rhythm Society S1547–5271, no. 23 (2023): 02999‐5, 10.1016/j.hrthm.2023.12.003. 38101500

[27] K. H. Kuck , J. Brugada , A. Fürnkranz , et al., “Cryoballoon or Radiofrequency Ablation for Paroxysmal Atrial Fibrillation,” New England Journal of Medicine 374, no. 23 (2016): 2235–2245, 10.1056/NEJMoa1602014. 27042964

[28] T. Serban , D. Mannhart , Q. Abid , et al., “Durability of Pulmonary Vein Isolation for Atrial Fibrillation: A Meta‐Analysis and Systematic Review,” Europace: European Pacing, Arrhythmias, and Cardiac Electrophysiology: Journal of the Working Groups on Cardiac Pacing, Arrhythmias, and Cardiac Cellular Electrophysiology of the European Society of Cardiology 25, no. 11 (2023): euad335, 10.1093/europace/euad335. 37944133 PMC10664405

[29] M. Thind , A. Oraii , C. Chaumont , et al., “Predictors of Nonpulmonary Vein Triggers for Atrial Fibrillation: A Clinical Risk Score,” Heart Rhythm: The Official Journal of the Heart Rhythm Society 21, no. 6 (2024): 806–811, 10.1016/j.hrthm.2024.01.048. 38296010

[30] H. Nakagawa , B. J. Scherlag , E. Patterson , A. Ikeda , D. Lockwood , and W. M. Jackman , “Pathophysiologic Basis of Autonomic Ganglionated Plexus Ablation in Patients With Atrial Fibrillation,” Heart Rhythm: The Official Journal of the Heart Rhythm Society 6, no. 12 Suppl (2009): S26–S34, 10.1016/j.hrthm.2009.07.029. 19959140

[31] P. Santangeli and F. E. Marchlinski , “Techniques for the Provocation, Localization, and Ablation of Non‐Pulmonary Vein Triggers for Atrial Fibrillation,” Heart Rhythm: The Official Journal of the Heart Rhythm Society 14, no. 7 (2017): 1087–1096, 10.1016/j.hrthm.2017.02.030. 28259694

[32] Y. Sakamoto , H. Osanai , S. Hiramatsu , et al., “Efficacy of Isoproterenol in the Evaluation of Dormant Conduction and Arrhythmogenic Foci Identification in Atrial Fibrillation Ablation,” BMC Cardiovascular Disorders 20 (2020): 397, 10.1186/s12872-020-01685-w. 32867695 PMC7461289

[33] M. Valderrábano , H. R. Chen , J. Sidhu , L. Rao , Y. Ling , and D. S. Khoury , “Retrograde Ethanol Infusion in the Vein of Marshall: Regional Left Atrial Ablation, Vagal Denervation and Feasibility in Humans,” Circulation: Arrhythmia and Electrophysiology 2, no. 1 (2009): 50–56, 10.1161/CIRCEP.108.818427. 19756206 PMC2743322

[34] L. Di Biase , J. D. Burkhardt , P. Mohanty , et al., “Left Atrial Appendage: An Underrecognized Trigger Site of Atrial Fibrillation,” Circulation 122, no. 2 (2010): 109–118, 10.1161/CIRCULATIONAHA.109.928903. 20606120

[35] C. H. Heeger , A. Rillig , D. Geisler , et al., “Left Atrial Appendage Isolation in Patients Not Responding to Pulmonary Vein Isolation,” Circulation 139, no. 5 (2019): 712–715, 10.1161/CIRCULATIONAHA.118.037451. 30689416

[36] J. L. Cox , “The Surgical Treatment of Atrial Fibrillation,” The Journal of Thoracic and Cardiovascular Surgery 101, no. 4 (1991): 584–592. 2008096

[37] X. Hu , J. Jiang , Y. Ma , and A. Tang , “Is There Still a Role for Additional Linear Ablation in Addition to Pulmonary Vein Isolation in Patients With Paroxysmal Atrial Fibrillation? An Updated Meta‐Analysis of Randomized Controlled Trials,” International Journal of Cardiology 209 (2016): 266–274, 10.1016/j.ijcard.2016.02.076. 26897081

[38] T. Takagi , N. Derval , J. Duchateau , et al., “Gaps After Linear Ablation of Persistent Atrial Fibrillation (Marshall‐Plan): Clinical Implication,” Heart Rhythm: The Official Journal of the Heart Rhythm Society 20, no. 1 (2023): 14–21, 10.1016/j.hrthm.2022.09.009. 36115541

[39] A. Verma , P. Novak , L. Macle , et al., “A Prospective, Multicenter Evaluation of Ablating Complex Fractionated Electrograms (CFEs) During Atrial Fibrillation (AF) Identified by an Automated Mapping Algorithm: Acute Effects on AF and Efficacy as an Adjuvant Strategy,” Heart Rhythm: The Official Journal of the Heart Rhythm Society 5, no. 2 (2008): 198–205, 10.1016/j.hrthm.2007.09.027. 18242539

[40] P. A. Scott , J. Silberbauer , and F. D. Murgatroyd , “The Impact of Adjunctive Complex Fractionated Atrial Electrogram Ablation and Linear Lesions on Outcomes in Persistent Atrial Fibrillation: A Meta‐Analysis,” Europace: European Pacing, Arrhythmias, and Cardiac Electrophysiology : Journal of the Working Groups on Cardiac Pacing, Arrhythmias, and Cardiac Cellular Electrophysiology of the European Society of Cardiology 18, no. 3 (2016): 359–367, 10.1093/europace/euv351. 26559915

[41] R. M. Hayward , G. A. Upadhyay , T. Mela , et al., “Pulmonary Vein Isolation With Complex Fractionated Atrial Electrogram Ablation for Paroxysmal and Nonparoxysmal Atrial Fibrillation: A Meta‐Analysis,” Heart Rhythm: The Official Journal of the Heart Rhythm Society 8, no. 7 (2011): 994–1000, 10.1016/j.hrthm.2011.02.033. PMC4224290 21397045

[42] J. M. Davidenko , P. F. Kent , D. R. Chialvo , D. C. Michaels , and J. Jalife , “Sustained Vortex‐Like Waves in Normal Isolated Ventricular Muscle,” Proceedings of the National Academy of Sciences 87, no. 22 (1990): 8785–8789, 10.1073/pnas.87.22.8785. PMC55044 2247448

[43] M. P. Nash , A. Mourad , R. H. Clayton , et al., “Evidence for Multiple Mechanisms in Human Ventricular Fibrillation,” Circulation 114, no. 6 (2006): 536–542, 10.1161/CIRCULATIONAHA.105.602870. 16880326

[44] O. Berenfeld and A. M. Pertsov , “Dynamics of Intramural Scroll Waves in Three‐Dimensional Continuous Myocardium With Rotational Anisotropy,” Journal of Theoretical Biology 199, no. 4 (1999): 383–394, 10.1006/jtbi.1999.0965. 10441456

[45] R. A. Gray , A. M. Pertsov , and J. Jalife , “Spatial and Temporal Organization During Cardiac Fibrillation,” Nature 392, no. 6671 (1998): 75–78, 10.1038/32164. 9510249

[46] J. M. Davidenko , A. V. Pertsov , R. Salomonsz , W. Baxter , and J. Jalife , “Stationary and Drifting Spiral Waves of Excitation in Isolated Cardiac Muscle,” Nature 355, no. 6358 (1992): 349–351, 10.1038/355349a0. 1731248

[47] S. Lévy , M. Maarek , P. Coumel , et al., “Characterization of Different Subsets of Atrial Fibrillation in General Practice in France: The ALFA Study,” Circulation 99, no. 23 (1999): 3028–3035, 10.1161/01.cir.99.23.3028. 10368121

[48] E. I. Charitos , H. Pürerfellner , T. V. Glotzer , and P. D. Ziegler , “Clinical Classifications of Atrial Fibrillation Poorly Reflect Its Temporal Persistence,” Journal of the American College of Cardiology 63, no. 25 Pt A (2014): 2840–2848, 10.1016/j.jacc.2014.04.019. 24814497

[49] B. Burstein and S. Nattel , “Atrial Fibrosis: Mechanisms and Clinical Relevance in Atrial Fibrillation,” Journal of the American College of Cardiology 51, no. 8 (2008): 802–809, 10.1016/j.jacc.2007.09.064. 18294563

[50] D. J. Wilber , C. Pappone , P. Neuzil , et al., “Comparison of Antiarrhythmic Drug Therapy and Radiofrequency Catheter Ablation in Patients With Paroxysmal Atrial Fibrillation: A Randomized Controlled Trial,” Journal of the American Medical Association 303, no. 4 (2010): 333–340, 10.1001/jama.2009.2029. 20103757

[51] D. L. Packer , D. B. Mark , R. A. Robb , et al., “Effect of Catheter Ablation vs Antiarrhythmic Drug Therapy on Mortality, Stroke, Bleeding, and Cardiac Arrest Among Patients With Atrial Fibrillation: The CABANA Randomized Clinical Trial,” Journal of the American Medical Association 321, no. 13 (2019): 1261–1274, 10.1001/jama.2019.0693. 30874766 PMC6450284

[52] D. L. Packer , R. C. Kowal , K. R. Wheelan , et al., “Cryoballoon Ablation of Pulmonary Veins for Paroxysmal Atrial Fibrillation,” Journal of the American College of Cardiology 61, no. 16 (2013): 1713–1723, 10.1016/j.jacc.2012.11.064. 23500312

[53] L. Mont , F. Bisbal , A. Hernández‐Madrid , et al., “Catheter Ablation vs. Antiarrhythmic Drug Treatment of Persistent Atrial Fibrillation: A Multicentre, Randomized, Controlled Trial (SARA Study),” European Heart Journal 35, no. 8 (2014): 501–507, 10.1093/eurheartj/eht457. 24135832 PMC3930872

[54] J. G. Andrade , O. M. Wazni , M. Kuniss , et al., “Cryoballoon Ablation as Initial Treatment for Atrial Fibrillation,” Journal of the American College of Cardiology 78, no. 9 (2021): 914–930, 10.1016/j.jacc.2021.06.038. 34446164

[55] O. M. Wazni , G. Dandamudi , N. Sood , et al., “Cryoballoon Ablation as Initial Therapy for Atrial Fibrillation,” New England Journal of Medicine 384, no. 4 (2021): 316–324, 10.1056/NEJMoa2029554. 33197158

[56] J. G. Andrade , M. W. Deyell , L. Macle , et al., “Progression of Atrial Fibrillation after Cryoablation or Drug Therapy,” New England Journal of Medicine 388, no. 2 (2023): 105–116, 10.1056/NEJMoa2212540. 36342178

[57] N. F. Marrouche , J. Brachmann , D. Andresen , et al., “Catheter Ablation for Atrial Fibrillation With Heart Failure,” New England Journal of Medicine 378, no. 5 (2018): 417–427, 10.1056/NEJMoa1707855. 29385358

[58] R. Dulai , N. Sulke , N. Freemantle , et al., “Pulmonary Vein Isolation vs Sham Intervention in Symptomatic Atrial Fibrillation: The SHAM‐PVI Randomized Clinical Trial,” Journal of the American Medical Association 332 (2024): 1165, 10.1001/jama.2024.17921. 39221629 PMC11369784

[59] J. G. Andrade , J. Champagne , M. Dubuc , et al., “Cryoballoon or Radiofrequency Ablation for Atrial Fibrillation Assessed by Continuous Monitoring: A Randomized Clinical Trial,” Circulation 140, no. 22 (2019): 1779–1788, 10.1161/CIRCULATIONAHA.119.042622. 31630538

[60] K. H. Kuck , D. S. Lebedev , E. N. Mikhaylov , et al., “Catheter Ablation or Medical Therapy to Delay Progression of Atrial Fibrillation: The Randomized Controlled Atrial Fibrillation Progression Trial (ATTEST),” EP Europace 23, no. 3 (2021): 362–369a, 10.1093/europace/euaa298. PMC7947582 33330909

[61] C. A. Morillo , A. Verma , S. J. Connolly , et al., “Radiofrequency Ablation vs Antiarrhythmic Drugs as First‐Line Treatment of Paroxysmal Atrial Fibrillation (RAAFT‐2): A Randomized Trial,” Journal of the American Medical Association 311, no. 7 (2014): 692–700, 10.1001/jama.2014.467. 24549549

[62] M. Valderrábano , L. E. Peterson , V. Swarup , et al., “Effect of Catheter Ablation With Vein of Marshall Ethanol Infusion vs Catheter Ablation Alone on Persistent Atrial Fibrillation: The VENUS Randomized Clinical Trial,” Journal of the American Medical Association 324, no. 16 (2020): 1620–1628, 10.1001/jama.2020.16195. 33107945 PMC7592031

[63] C. Sohns , H. Fox , N. F. Marrouche , et al., “Catheter Ablation in End‐Stage Heart Failure With Atrial Fibrillation,” New England Journal of Medicine 389, no. 15 (2023): 1380–1389, 10.1056/NEJMoa2306037. 37634135

[64] A. M. Al‐Kaisey , R. Parameswaran , C. Bryant , et al., “Atrial Fibrillation Catheter Ablation vs Medical Therapy and Psychological Distress: A Randomized Clinical Trial,” Journal of the American Medical Association 330, no. 10 (2023): 925–933, 10.1001/jama.2023.14685. 37698564 PMC10498333

[65] Y. Huo , T. Gaspar , R. Schönbauer , et al., “Low‐Voltage Myocardium‐Guided Ablation Trial of Persistent Atrial Fibrillation,” NEJM Evidence 1, no. 11 (2022): EVIDoa2200141, 10.1056/EVIDoa2200141. 38319851

[66] R. Providencia , H. Ali , S. Barra , A. Creta , et al., “Ablation of Atrial Fibrillation and Risk of Stroke: A Meta‐Analysis,” *Heart Rhythm* (2025): 22. 10.1016/j.hrthm.2025.05.021 40398548

[67] R. Providencia , H. Ali , A. Creta , et al., “Catheter Ablation for Atrial Fibrillation and Impact on Clinical Outcomes,” European Heart Journal Open 4, no. 4 (2024): oeae058, 10.1093/ehjopen/oeae058. 39143978 PMC11322836

[68] K. H. Kuck , A. Fürnkranz , K. R. J. Chun , et al., “Cryoballoon or Radiofrequency Ablation for Symptomatic Paroxysmal Atrial Fibrillation: Reintervention, Rehospitalization, and Quality‐Of‐Life Outcomes in the FIRE AND ICE Trial,” European Heart Journal 37, no. 38 (2016): 2858–2865, 10.1093/eurheartj/ehw285. 27381589 PMC5070448

[69] V. Y. Reddy , E. P. Gerstenfeld , A. Natale , et al., “Pulsed Field or Conventional Thermal Ablation for Paroxysmal Atrial Fibrillation,” New England Journal of Medicine 389, no. 18 (2023): 1660–1671, 10.1056/NEJMoa2307291. 37634148

[70] E. Anter , M. Mansour , D. G. Nair , et al., “Dual‐Energy Lattice‐Tip Ablation System for Persistent Atrial Fibrillation: A Randomized Trial,” Nature Medicine 30, no. 8 (2024): 2303–2310, 10.1038/s41591-024-03022-6. PMC11333282 38760584

[71] J. P. T. Higgins , D. G. Altman , P. C. Gotzsche , et al., “The Cochrane Collaboration's Tool for Assessing Risk of Bias in Randomised Trials,” BMJ 343 (2011): d5928, 10.1136/bmj.d5928. 22008217 PMC3196245

[72] G. H. Guyatt , A. D. Oxman , G. E. Vist , et al., “Grade: An Emerging Consensus on Rating Quality of Evidence and Strength of Recommendations,” BMJ 336, no. 7650 (2008): 924–926, 10.1136/bmj.39489.470347.AD. 18436948 PMC2335261

[73] N. F. Marrouche , O. Wazni , C. McGann , et al., “Effect of MRI‐Guided Fibrosis Ablation vs Conventional Catheter Ablation on Atrial Arrhythmia Recurrence in Patients With Persistent Atrial Fibrillation: The DECAAF II Randomized Clinical Trial,” Journal of the American Medical Association 327, no. 23 (2022): 2296–2305, 10.1001/jama.2022.8831. 35727277 PMC9214588

[74] A. Verma , C. Jiang , T. R. Betts , et al., “Approaches to Catheter Ablation for Persistent Atrial Fibrillation,” New England Journal of Medicine 372, no. 19 (2015): 1812–1822, 10.1056/NEJMoa1408288. 25946280

[75] P. M. Kistler , D. Chieng , H. Sugumar , et al., “Effect of Catheter Ablation Using Pulmonary Vein Isolation With vs Without Posterior Left Atrial Wall Isolation on Atrial Arrhythmia Recurrence in Patients With Persistent Atrial Fibrillation: The CAPLA Randomized Clinical Trial,” Journal of the American Medical Association 329, no. 2 (2023): 127–135, 10.1001/jama.2022.23722. 36625809 PMC9856612

[76] D. R. Lakkireddy , D. J. Wilber , S. Mittal , et al., “Pulmonary Vein Isolation With or Without Left Atrial Appendage Ligation in Atrial Fibrillation: The aMAZE Randomized Clinical Trial,” Journal of the American Medical Association 331, no. 13 (2024): 1099–1108, 10.1001/jama.2024.3026. 38563835 PMC10988350

[77] F. Gaita , D. Caponi , M. Scaglione , et al., “Long‐Term Clinical Results of 2 Different Ablation Strategies in Patients With Paroxysmal and Persistent Atrial Fibrillation,” Circulation: Arrhythmia and Electrophysiology 1, no. 4 (2008): 269–275, 10.1161/CIRCEP.108.774885. 19808418

[78] G. Fassini , S. Riva , R. Chiodelli , et al., “Left Mitral Isthmus Ablation Associated With PV Isolation: Long‐Term Results of a Prospective Randomized Study,” Journal of Cardiovascular Electrophysiology 16, no. 11 (2005): 1150–1156, 10.1111/j.1540-8167.2005.50192.x. 16302895

[79] M. Hocini , P. Jaïs , P. Sanders , et al., “Techniques, Evaluation, and Consequences of Linear Block at the Left Atrial Roof in Paroxysmal Atrial Fibrillation: A Prospective Randomized Study,” Circulation 112, no. 24 (2005): 3688–3696, 10.1161/CIRCULATIONAHA.105.541052. 16344401

[80] S. Willems , H. Klemm , T. Rostock , et al., “Substrate Modification Combined With Pulmonary Vein Isolation Improves Outcome of Catheter Ablation in Patients With Persistent Atrial Fibrillation: A Prospective Randomized Comparison,” European Heart Journal 27, no. 23 (2006): 2871–2878, 10.1093/eurheartj/ehl093. 16782716

[81] I. Sheikh , D. Krum , R. Cooley , et al., “Pulmonary Vein Isolation and Linear Lesions in Atrial Fibrillation Ablation,” Journal of Interventional Cardiac Electrophysiology 17, no. 2 (2007): 103–109, 10.1007/s10840-006-9066-9. 17318445

[82] H. S. Mun , B. Joung , J. Shim , et al., “Does Additional Linear Ablation After Circumferential Pulmonary Vein Isolation Improve Clinical Outcome in Patients With Paroxysmal Atrial Fibrillation? Prospective Randomised Study,” Heart 98, no. 6 (2012): 480–484, 10.1136/heartjnl-2011-301107. 22285969 PMC3285139

[83] E. Arbelo , E. Guiu , P. Ramos , et al., “Benefit of Left Atrial Roof Linear Ablation in Paroxysmal Atrial Fibrillation: A Prospective, Randomized Study,” Journal of the American Heart Association 3, no. 5 (2014): e000877, 10.1161/JAHA.114.000877. 25193295 PMC4323787

[84] G. J. Wynn , S. Panikker , M. Morgan , et al., “Biatrial Linear Ablation in Sustained Nonpermanent Af: Results of the Substrate Modification With Ablation and Antiarrhythmic Drugs in Nonpermanent Atrial Fibrillation (SMAN‐PAF) Trial,” Heart Rhythm: The Official Journal of the Heart Rhythm Society 13, no. 2 (2016): 399–406, 10.1016/j.hrthm.2015.10.006. 26455343

[85] H. T. Yu , J. Shim , J. Park , et al., “Pulmonary Vein Isolation Alone Versus Additional Linear Ablation in Patients With Persistent Atrial Fibrillation Converted to Paroxysmal Type With Antiarrhythmic Drug Therapy: A Multicenter, Prospective, Randomized Study,” Circulation: Arrhythmia and Electrophysiology 10, no. 6 (2017): e004915, 10.1161/CIRCEP.116.004915. 28611206

[86] H. Oral , A. Chugh , K. Yoshida , et al., “A Randomized Assessment of the Incremental Role of Ablation of Complex Fractionated Atrial Electrograms after Antral Pulmonary Vein Isolation for Long‐Lasting Persistent Atrial Fibrillation,” Journal of the American College of Cardiology 53, no. 9 (2009): 782–789, 10.1016/j.jacc.2008.10.054. 19245970

[87] I. Deisenhofer , H. Estner , T. Reents , et al., “Does Electrogram Guided Substrate Ablation Add to the Success of Pulmonary Vein Isolation in Patients With Paroxysmal Atrial Fibrillation? A Prospective, Randomized Study,” Journal of Cardiovascular Electrophysiology 20, no. 5 (2009): 514–521, 10.1111/j.1540-8167.2008.01379.x. 19207759

[88] C. S. Elayi , A. Verma , L. Di Biase , et al., “Ablation for Longstanding Permanent Atrial Fibrillation: Results From a Randomized Study Comparing Three Different Strategies,” Heart Rhythm: The Official Journal of the Heart Rhythm Society 5, no. 12 (2008): 1658–1664, 10.1016/j.hrthm.2008.09.016. 19084800

[89] L. Di Biase , C. S. Elayi , T. S. Fahmy , et al., “Atrial Fibrillation Ablation Strategies for Paroxysmal Patients: Randomized Comparison Between Different Techniques,” Circulation: Arrhythmia and Electrophysiology 2, no. 2 (2009): 113–119, 10.1161/CIRCEP.108.798447. 19808455

[90] A. Verma , R. Mantovan , L. Macle , et al., “Substrate and Trigger Ablation for Reduction of Atrial Fibrillation (STAR AF): A Randomized, Multicentre, International Trial,” European Heart Journal 31, no. 11 (2010): 1344–1356, 10.1093/eurheartj/ehq041. 20215126 PMC2878965

[91] M. Chen , B. Yang , H. Chen , et al., “Randomized Comparison Between Pulmonary Vein Antral Isolation Versus Complex Fractionated Electrogram Ablation for Paroxysmal Atrial Fibrillation,” Journal of Cardiovascular Electrophysiology 22, no. 9 (2011): 973–981, 10.1111/j.1540-8167.2011.02051.x. 21539635

[92] H. Oral , A. Chugh , K. Lemola , et al., “Noninducibility of Atrial Fibrillation as an End Point of Left Atrial Circumferential Ablation for Paroxysmal Atrial Fibrillation: A Randomized Study,” Circulation 110, no. 18 (2004): 2797–2801, 10.1161/01.CIR.0000146786.87037.26. 15505091

[93] R. Providência , P. D. Lambiase , N. Srinivasan , et al., “Is There Still a Role for Complex Fractionated Atrial Electrogram Ablation in Addition to Pulmonary Vein Isolation in Patients With Paroxysmal and Persistent Atrial Fibrillation? Meta‐Analysis of 1415 Patients,” Circulation: Arrhythmia and Electrophysiology 8, no. 5 (2015): 1017–1029, 10.1161/CIRCEP.115.003019. 26082515

[94] D. G. Katritsis , E. Pokushalov , A. Romanov , et al., “Autonomic Denervation Added to Pulmonary Vein Isolation for Paroxysmal Atrial Fibrillation,” Journal of the American College of Cardiology 62, no. 24 (2013): 2318–2325, 10.1016/j.jacc.2013.06.053. 23973694

[95] E. Pokushalov , A. Romanov , D. G. Katritsis , et al., “Ganglionated Plexus Ablation vs Linear Ablation in Patients Undergoing Pulmonary Vein Isolation for Persistent/Long‐Standing Persistent Atrial Fibrillation: A Randomized Comparison,” Heart Rhythm: The Official Journal of the Heart Rhythm Society 10, no. 9 (2013): 1280–1286, 10.1016/j.hrthm.2013.04.016. 23608592

[96] M. Y. Kim , C. Coyle , D. R. Tomlinson , et al., “Ectopy‐Triggering Ganglionated Plexuses Ablation to Prevent Atrial Fibrillation: Ganglia‐Af Study,” Heart Rhythm: The Official Journal of the Heart Rhythm Society 19, no. 4 (2022): 516–524, 10.1016/j.hrthm.2021.12.010. PMC8976158 34915187

[97] A. H. G. Driessen , W. R. Berger , S. P. J. Krul , et al., “Ganglion Plexus Ablation in Advanced Atrial Fibrillation,” Journal of the American College of Cardiology 68, no. 11 (2016): 1155–1165, 10.1016/j.jacc.2016.06.036. 27609676

[98] N. Morita , T. Iida , T. Nanao , et al., “Effect of Ganglionated Plexi Ablation by High‐Density Mapping on Long‐Term Suppression of Paroxysmal Atrial Fibrillation – The First Clinical Survey on Ablation of the Dorsal Right Plexus,” Heart Rhythm O2 2, no. 5 (2021): 480–488, 10.1016/j.hroo.2021.07.002. 34667963 PMC8505203

[99] W. R. Berger , J. Neefs , N. W. E. van den Berg , et al., “Additional Ganglion Plexus Ablation During Thoracoscopic Surgical Ablation of Advanced Atrial Fibrillation,” JACC: Clinical Electrophysiology 5, no. 3 (2019): 343–353, 10.1016/j.jacep.2018.10.008. 30898238

[100] E. Pokushalov , A. Romanov , P. Shugayev , et al., “Selective Ganglionated Plexi Ablation for Paroxysmal Atrial Fibrillation,” Heart Rhythm: The Official Journal of the Heart Rhythm Society 6, no. 9 (2009): 1257–1264, 10.1016/j.hrthm.2009.05.018. 19656736

[101] S. M. Narayan , D. E. Krummen , K. Shivkumar , P. Clopton , W. J. Rappel , and J. M. Miller , “Treatment of Atrial Fibrillation by the Ablation of Localized Sources,” Journal of the American College of Cardiology 60, no. 7 (2012): 628–636, 10.1016/j.jacc.2012.05.022. 22818076 PMC3416917

[102] S. M. Narayan , T. Baykaner , P. Clopton , et al., “Ablation of Rotor and Focal Sources Reduces Late Recurrence of Atrial Fibrillation Compared With Trigger Ablation Alone: Extended Follow‐Up of the CONFIRM trial (Conventional Ablation for Atrial Fibrillation With or Without Focal Impulse and Rotor Modulation),” Journal of the American College of Cardiology 63, no. 17 (2014): 1761–1768, 10.1016/j.jacc.2014.02.543. 24632280 PMC4008643

[103] G. Tomassoni , S. Duggal , M. Muir , et al., “Long‐Term Follow‐Up of FIRM‐Guided Ablation of Atrial Fibrillation: A Single‐Center Experience,” The Journal of Innovations in Cardiac Rhythm Management 6 (2015): 2145–2151.

[104] E. Buch , M. Share , R. Tung , et al., “Long‐Term Clinical Outcomes of Focal Impulse and Rotor Modulation for Treatment of Atrial Fibrillation: A Multicenter Experience,” Heart Rhythm: The Official Journal of the Heart Rhythm Society 13, no. 3 (2016): 636–641, 10.1016/j.hrthm.2015.10.031. PMC4762742 26498260

[105] C. Gianni , S. Mohanty , L. Di Biase , et al., “Acute and Early Outcomes of Focal Impulse and Rotor Modulation (FIRM)‐Guided Rotors‐Only Ablation in Patients With Nonparoxysmal Atrial Fibrillation,” Heart Rhythm: The Official Journal of the Heart Rhythm Society 13, no. 4 (2016): 830–835, 10.1016/j.hrthm.2015.12.028. 26706193

[106] B. P. Knight , “Anticoagulation for Atrial Fibrillation Ablation,” Journal of the American College of Cardiology 59, no. 13 (2012): 1175–1177, 10.1016/j.jacc.2011.11.044. 22305112

[107] L. Di Biase , J. D. Burkhardt , P. Santangeli , et al., “Periprocedural Stroke and Bleeding Complications in Patients Undergoing Catheter Ablation of Atrial Fibrillation With Different Anticoagulation Management: Results From the Role of Coumadin in Preventing Thromboembolism in Atrial Fibrillation (AF) Patients Undergoing Catheter Ablation (COMPARE) Randomized Trial,” Circulation 129, no. 25 (2014): 2638–2644, 10.1161/CIRCULATIONAHA.113.006426. 24744272

[108] H. L. Armbruster , J. P. Lindsley , M. P. Moranville , et al., “Safety of Novel Oral Anticoagulants Compared With Uninterrupted Warfarin for Catheter Ablation of Atrial Fibrillation,” Annals of Pharmacotherapy 49, no. 3 (2015): 278–284, 10.1177/1060028014563950. 25515868

[109] Y. Zhao , Y. Lu , and Y. Qin , “A Meta‐Analysis of Randomized Controlled Trials of Uninterrupted Periprocedural Anticoagulation Strategy in Patients Undergoing Atrial Fibrillation Catheter Ablation,” International Journal of Cardiology 270 (2018): 167–171, 10.1016/j.ijcard.2018.06.024. 29903520

[110] S. P. G. van Vugt , S. W. Westra , R. H. J. A. Volleberg , et al., “Meta‐Analysis of Controlled Studies on Minimally Interrupted Vs. Continuous Use of Non‐Vitamin K Antagonist Oral Anticoagulants in Catheter Ablation for Atrial Fibrillation,” EP Europace 23, no. 12 (2021): 1961–1969, 10.1093/europace/euab175. PMC8651164 34333631

[111] R. Cappato , F. E. Marchlinski , S. H. Hohnloser , et al., “Uninterrupted Rivaroxaban vs. Uninterrupted Vitamin K Antagonists for Catheter Ablation in Non‐Valvular Atrial Fibrillation,” European Heart Journal 36, no. 28 (2015): 1805–1811, 10.1093/eurheartj/ehv177. 25975659 PMC4508487

[112] H. Calkins , S. Willems , E. P. Gerstenfeld , et al., “Uninterrupted Dabigatran Versus Warfarin for Ablation in Atrial Fibrillation,” New England Journal of Medicine 376, no. 17 (2017): 1627–1636, 10.1056/NEJMoa1701005. 28317415

[113] P. Kirchhof , K. G. Haeusler , B. Blank , et al., “Apixaban in Patients at Risk of Stroke Undergoing Atrial Fibrillation Ablation,” European Heart Journal 39, no. 32 (2018): 2942–2955, 10.1093/eurheartj/ehy176. 29579168 PMC6110196

[114] S. H. Hohnloser , J. Camm , R. Cappato , et al., “Uninterrupted Edoxaban vs. Vitamin K Antagonists for Ablation of Atrial Fibrillation: The ELIMINATE‐AF Trial,” European Heart Journal 40, no. 36 (2019): 3013–3021, 10.1093/eurheartj/ehz190. 30976787 PMC6754569

[115] A. Yoshimura , Y. Iriki , H. Ichiki , et al., “Evaluation of Safety and Efficacy of Periprocedural Use of Rivaroxaban and Apixaban in Catheter Ablation for Atrial Fibrillation,” Journal of Cardiology 69, no. 1 (2017): 228–235, 10.1016/j.jjcc.2016.03.014. 27131792

[116] T. Kuwahara , M. Abe , M. Yamaki , et al., “Apixaban Versus Warfarin for the Prevention of Periprocedural Cerebral Thromboembolism in Atrial Fibrillation Ablation: Multicenter Prospective Randomized Study,” Journal of Cardiovascular Electrophysiology 27, no. 5 (2016): 549–554, 10.1111/jce.12928. 26766541

[117] T. Kimura , S. Kashimura , T. Nishiyama , et al., “Asymptomatic Cerebral Infarction During Catheter Ablation for Atrial Fibrillation,” JACC: Clinical Electrophysiology 4, no. 12 (2018): 1598–1609, 10.1016/j.jacep.2018.08.003. 30573125

[118] A. Nogami , T. Harada , Y. Sekiguchi , et al., “Safety and Efficacy of Minimally Interrupted Dabigatran vs Uninterrupted Warfarin Therapy in Adults Undergoing Atrial Fibrillation Catheter Ablation: A Randomized Clinical Trial,” JAMA Network Open 2, no. 4 (2019): e191994, 10.1001/jamanetworkopen.2019.1994. 31002317 PMC6481436

[119] T. Nagao , H. Suzuki , S. Matsunaga , et al., “Impact of Periprocedural Anticoagulation Therapy on the Incidence of Silent Stroke after Atrial Fibrillation Ablation in Patients Receiving Direct Oral Anticoagulants: Uninterrupted Vs. Interrupted By One Dose Strategy,” EP Europace 21, no. 4 (2019): 590–597, 10.1093/europace/euy224. 30376051

[120] P. Maury , S. Belaid , A. Ribes , et al., “Coagulation and Heparin Requirements During Ablation in Patients Under Oral Anticoagulant Drugs,” Journal of Arrhythmia 36, no. 4 (2020): 644–651, 10.1002/joa3.12357. 32782635 PMC7411209

[121] K. Kanaoka , T. Nishida , Y. Iwanaga , et al., “Oral Anticoagulation After Atrial Fibrillation Catheter Ablation: Benefits and Risks,” European Heart Journal 45, no. 7 (2024): 522–534, 10.1093/eurheartj/ehad798. 38117227 PMC10873714

[122] J. W. Schrickel , T. Beiert , M. Linhart , et al., “Prevention of Cerebral Thromboembolism by Oral Anticoagulation With Dabigatran After Pulmonary Vein Isolation for Atrial Fibrillation: The ODIn‐Af Trial,” Clinical Research in Cardiology 113, no. 8 (2024): 1183–1199, 10.1007/s00392-023-02319-9. 37921923 PMC11269394

[123] M. R. Reynolds , J. S. Allison , A. Natale , et al., “A Prospective Randomized Trial of Apixaban Dosing During Atrial Fibrillation Ablation: The AEIOU Trial,” JACC: Clinical Electrophysiology 4, no. 5 (2018): 580–588, 10.1016/j.jacep.2017.11.005. 29798783

[124] M. Ando , Y. Inden , Y. Yoshida , et al., “Differences in Prothrombotic Response Between the Uninterrupted and Interrupted Apixaban Therapies in Patients Undergoing Cryoballoon Ablation for Paroxysmal Atrial Fibrillation: A Randomized Controlled Study,” Heart and Vessels 34, no. 9 (2019): 1533–1541, 10.1007/s00380-019-01370-9. 30840130

[125] H. T. Yu , J. Shim , J. Park , et al., “When Is It Appropriate to Stop Non‐Vitamin K Antagonist Oral Anticoagulants Before Catheter Ablation of Atrial Fibrillation? A Multicentre Prospective Randomized Study,” European Heart Journal 40, no. 19 (2019): 1531–1537, 10.1093/eurheartj/ehy870. 30590600

[126] K. Nakamura , S. Naito , T. Sasaki , et al., “Uninterrupted vs. Interrupted Periprocedural Direct Oral Anticoagulants for Catheter Ablation of Atrial Fibrillation: A Prospective Randomized Single‐Centre Study on Post‐Ablation Thrombo‐Embolic and Haemorrhagic Events,” EP Europace 21, no. 2 (2019): 259–267, 10.1093/europace/euy148. 29982562

[127] G. A. Bawazeer , H. A. Alkofide , A. A. Alsharafi , et al., “Interrupted Versus Uninterrupted Anticoagulation Therapy for Catheter Ablation in Adults With Arrhythmias,” The Cochrane Database of Systematic Reviews 10, no. 10 (2021): 013504, 10.1002/14651858.CD013504.pub2. PMC8530018 34674223

[128] J. Romero , R. C. Cerrud‐Rodriguez , I. Alviz , et al., “Significant Benefit of Uninterrupted DOACs Versus VKA During Catheter Ablation of Atrial Fibrillation,” JACC: Clinical Electrophysiology 5, no. 12 (2019): 1396–1405, 10.1016/j.jacep.2019.08.010. 31857038

[129] R. R. Tilz , M. Feher , J. Vogler , et al., “Venous Vascular Closure System vs. Figure‐of‐Eight Suture Following Atrial Fibrillation Ablation: The Style‐Af Study,” Europace : European Pacing, Arrhythmias, and Cardiac Electrophysiology : Journal of the Working Groups on Cardiac Pacing, Arrhythmias, and Cardiac Cellular Electrophysiology of the European Society of Cardiology 26, no. 5 (2024): euae105, 10.1093/europace/euae105. 38647070 PMC11210072

[130] H. Lodhi , S. Shaukat , A. Mathews , B. Maini , and H. Khalili , “Comparison of Figure‐of‐Eight Suture and Perclose ProGlide Suture‐Mediated Closure in Large Bore Venous Access Hemostasis: A Randomized Controlled Trial,” The American Journal of Cardiology 209 (2023): 181–183, 10.1016/j.amjcard.2023.09.105. 37863115

[131] M. Ghannam , A. Chugh , P. Dillon , et al., “Protamine to Expedite Vascular Hemostasis after Catheter Ablation of Atrial Fibrillation: A Randomized Controlled Trial,” Heart Rhythm: The Official Journal of the Heart Rhythm Society 15, no. 11 (2018): 1642–1647, 10.1016/j.hrthm.2018.06.045. 30661768

[132] K. Yamagata , D. Wichterle , T. Roubíček , et al., “Ultrasound‐Guided Versus Conventional Femoral Venipuncture for Catheter Ablation of Atrial Fibrillation: A Multicentre Randomized Efficacy and Safety Trial (ULTRA‐FAST Trial),” EP Europace 20, no. 7 (2018): 1107–1114, 10.1093/europace/eux175. 28575490

[133] A. Deshmukh , N. J. Patel , S. Pant , et al., “In‐Hospital Complications Associated With Catheter Ablation of Atrial Fibrillation in the United States between 2000 and 2010: Analysis of 93 801 Procedures,” Circulation 128, no. 19 (2013): 2104–2112, 10.1161/CIRCULATIONAHA.113.003862. 24061087

[134] Z. Loring , D. N. Holmes , R. A. Matsouaka , et al., “Procedural Patterns and Safety of Atrial Fibrillation Ablation: Findings From Get With The Guidelines‐Atrial Fibrillation,” Circulation: Arrhythmia and Electrophysiology 13, no. 9 (2020): e007944, 10.1161/CIRCEP.119.007944. 32703018 PMC7502261

[135] D. Darden , O. Aldaas , C. Du , et al., “In‐Hospital Complications Associated With Pulmonary Vein Isolation With Adjunctive Lesions: the NCDR AFib Ablation Registry,” Europace : European Pacing, Arrhythmias, and Cardiac Electrophysiology : Journal of the Working Groups on Cardiac Pacing, Arrhythmias, and Cardiac Cellular Electrophysiology of the European Society of Cardiology 25, no. 5 (2023): euad124, 10.1093/europace/euad124. 37184436 PMC10228609

[136] L. Ngo , R. Denman , K. Hay , B. Kaambwa , A. Ganesan , and I. Ranasinghe , “Excess Bed Days and Hospitalization Costs Associated With 30‐Day Complications Following Catheter Ablation of Atrial Fibrillation,” Journal of the American Heart Association 12, no. 23 (2023): e030236, 10.1161/JAHA.123.030236. 38038189 PMC10727335

[137] K. Benali , P. Khairy , N. Hammache , et al., “Procedure‐Related Complications of Catheter Ablation for Atrial Fibrillation,” Journal of the American College of Cardiology 81, no. 21 (2023): 2089–2099, 10.1016/j.jacc.2023.03.418. 37225362

[138] E. Ekanem , V. Y. Reddy , B. Schmidt , et al., “Multi‐National Survey on the Methods, Efficacy, and Safety on the Post‐Approval Clinical Use of Pulsed Field Ablation (MANIFEST‐PF),” EP Europace 24, no. 8 (2022): 1256–1266, 10.1093/europace/euac050. 35647644 PMC9435639

[139] K. Maleki , R. Mohammadi , D. Hart , D. Cotiga , N. Farhat , and J. S. Steinberg , “Intracardiac Ultrasound Detection of Thrombus on Transseptal Sheath: Incidence, Treatment, and Prevention,” Journal of Cardiovascular Electrophysiology 16, no. 6 (2005): 561–565, 10.1111/j.1540-8167.2005.40686.x. 15946349

[140] M. Goyal , B. K. Menon , W. H. van Zwam , et al., “Endovascular Thrombectomy After Large‐Vessel Ischaemic Stroke: A Meta‐Analysis of Individual Patient Data From Five Randomised Trials,” The Lancet 387, no. 10029 (2016): 1723–1731, 10.1016/S0140-6736(16)00163-X. 26898852

[141] European Stroke Organisation (ESO) guidelines on intravenous thrombolysis for acute ischaemic stroke – PubMed , Accessed; August 23, 2024, https://pubmed-ncbi-nlm-nih-gov.bibliosan.idm.oclc.org/33817340/. 10.1177/2396987321989865 PMC7995316 33817340

[142] C. H. Heeger , C. Sohns , A. Pott , et al., “Phrenic Nerve Injury During Cryoballoon‐Based Pulmonary Vein Isolation: Results of the Worldwide YETI Registry,” Circulation: Arrhythmia and Electrophysiology 15, no. 1 (2022): e010516, 10.1161/CIRCEP.121.010516. 34962134 PMC8772436

[143] R. R. Tilz , V. Schmidt , H. Pürerfellner , et al., “A Worldwide Survey on Incidence, Management, and Prognosis of Oesophageal Fistula Formation Following Atrial Fibrillation Catheter Ablation: The POTTER‐AF Study,” European Heart Journal 44, no. 27 (2023): 2458–2469, 10.1093/eurheartj/ehad250. 37062040 PMC10344651

[144] J. Sanchez , C. Woods , J. Zagrodzky , et al., “Atrioesophageal Fistula Rates Before and After Adoption of Active Esophageal Cooling During Atrial Fibrillation Ablation,” JACC: Clinical Electrophysiology 9, no. 12 (2023): 2558–2570, 10.1016/j.jacep.2023.08.022. 37737773

[145] C. M. Tschabrunn , S. Attalla , J. Salas , et al., “Active Esophageal Cooling for the Prevention of Thermal Injury During Atrial Fibrillation Ablation: A Randomized Controlled Pilot Study,” Journal of Interventional Cardiac Electrophysiology 63, no. 1 (2022): 197–205, 10.1007/s10840-021-00960-w. 33620619

[146] R. Cappato , H. Calkins , S. A. Chen , et al., “Prevalence and Causes of Fatal Outcome in Catheter Ablation of Atrial Fibrillation,” Journal of the American College of Cardiology 53, no. 19 (2009): 1798–1803, 10.1016/j.jacc.2009.02.022. 19422987

[147] E. P. Cheng , C. F. Liu , I. Yeo , et al., “Risk of Mortality Following Catheter Ablation of Atrial Fibrillation,” Journal of the American College of Cardiology 74, no. 18 (2019): 2254–2264, 10.1016/j.jacc.2019.08.1036. 31672181

[148] L. Ngo , A. Ali , A. Ganesan , R. Woodman , R. Adams , and I. Ranasinghe , “Ten‐Year Trends in Mortality and Complications Following Catheter Ablation of Atrial Fibrillation,” European Heart Journal ‐ Quality of Care and Clinical Outcomes 8, no. 4 (2022): 398–408, 10.1093/ehjqcco/qcab102. 34982824

[149] V. Y. Reddy , P. Neuzil , J. S. Koruth , et al., “Pulsed Field Ablation for Pulmonary Vein Isolation in Atrial Fibrillation,” Journal of the American College of Cardiology 74, no. 3 (2019): 315–326, 10.1016/j.jacc.2019.04.021. 31085321

[150] S. Venier , N. Vaxelaire , P. Jacon , et al., “Severe Acute Kidney Injury Related to Haemolysis After Pulsed Field Ablation for Atrial Fibrillation,” Europace: European Pacing, Arrhythmias, and Cardiac Electrophysiology : Journal of the Working Groups on Cardiac Pacing, Arrhythmias, and Cardiac Cellular Electrophysiology of the European Society of Cardiology 26, no. 1 (2023): euad371, 10.1093/europace/euad371. 38175788 PMC10776308

[151] C. Patel , E. P. Gerstenfeld , S. K. Gupta , et al., “Comparison of Cerebral Safety After Atrial Fibrillation Using Pulsed Field and Thermal Ablation: Results of the Neurological Assessment Subgroup in the ADVENT Trial,” Heart rhythm : the official journal of the Heart Rhythm Society S1547–5271, no. 24 (2024): 02661–02664, 10.1016/j.hrthm.2024.05.048. 38823667

[152] M. A. Gunawardene , B. N. Schaeffer , M. Jularic , et al., “Coronary Spasm During Pulsed Field Ablation of the Mitral Isthmus Line,” JACC: Clinical Electrophysiology 7, no. 12 (2021): 1618–1620, 10.1016/j.jacep.2021.08.016. 34600850

[153] P. Osmancik , B. Bacova , D. Herman , et al., “Periprocedural Intravascular Hemolysis During Atrial Fibrillation Ablation,” JACC: Clinical Electrophysiology 10, no. 7 Pt 2 (2024): 1660–1671, 10.1016/j.jacep.2024.05.001. 38852101

[154] H. Nakagawa , Q. Castellvi , R. Neal , et al., “Effects of Contact Force on Lesion Size During Pulsed Field Catheter Ablation: Histochemical Characterization of Ventricular Lesion Boundaries,” Circulation: Arrhythmia and Electrophysiology 17, no. 1 (2024): e012026, 10.1161/CIRCEP.123.012026. 38152949

[155] E. Ekanem , P. Neuzil , T. Reichlin , et al., “Safety of Pulsed Field Ablation in More Than 17,000 Patients With Atrial Fibrillation in the MANIFEST‐17K Study,” Nature Medicine 30, no. 7 (2024): 2020–2029, 10.1038/s41591-024-03114-3. PMC11271404 38977913

[156] P. Kirchhof , A. J. Camm , A. Goette , et al., “Early Rhythm‐Control Therapy in Patients With Atrial Fibrillation,” New England Journal of Medicine 383, no. 14 (2020): 1305–1316, 10.1056/NEJMoa2019422. 32865375

[157] R. J. Hunter , T. J. Berriman , I. Diab , et al., “A Randomized Controlled Trial of Catheter Ablation Versus Medical Treatment of Atrial Fibrillation in Heart Failure (The CAMTAF Trial),” Circulation: Arrhythmia and Electrophysiology 7, no. 1 (2014): 31–38, 10.1161/CIRCEP.113.000806. 24382410

[158] S. Prabhu , A. J. Taylor , B. T. Costello , et al., “Catheter Ablation Versus Medical Rate Control in Atrial Fibrillation and Systolic Dysfunction,” Journal of the American College of Cardiology 70, no. 16 (2017): 1949–1961, 10.1016/j.jacc.2017.08.041. 28855115

[159] L. Di Biase , P. Mohanty , S. Mohanty , et al., “Ablation Versus Amiodarone for Treatment of Persistent Atrial Fibrillation in Patients With Congestive Heart Failure and an Implanted Device: Results From the AATAC Multicenter Randomized Trial,” Circulation 133, no. 17 (2016): 1637–1644, 10.1161/CIRCULATIONAHA.115.019406. 27029350

[160] R. Parkash , G. A. Wells , J. Rouleau , et al., “Randomized Ablation‐Based Rhythm‐Control Versus Rate‐Control Trial in Patients With Heart Failure and Atrial Fibrillation: Results From the RAFT‐AF Trial,” Circulation 145, no. 23 (2022): 1693–1704, 10.1161/CIRCULATIONAHA.121.057095. 35313733

[161] M. Brignole , F. Pentimalli , P. Palmisano , et al., “AV Junction Ablation and Cardiac Resynchronization for Patients With Permanent Atrial Fibrillation and Narrow QRS: The APAF‐CRT Mortality Trial,” European Heart Journal 42, no. 46 (2021): 4731–4739, 10.1093/eurheartj/ehab569. 34453840

[162] P. Ponikowski , A. A. Voors , S. D. Anker , et al., “2016 ESC Guidelines for the Diagnosis and Treatment of Acute and Chronic Heart Failure: The Task Force for the Diagnosis and Treatment of Acute and Chronic Heart Failure of the European Society of Cardiology (ESC)Developed With the Special Contribution of the Heart Failure Association (HFA) of the ESC,” European Heart Journal 37, no. 27 (2016): 2129–2200, 10.1093/eurheartj/ehw128. 27206819

[163] R. Cappato , H. Calkins , S. A. Chen , et al., “Worldwide Survey on the Methods, Efficacy, and Safety of Catheter Ablation for Human Atrial Fibrillation,” Circulation 111, no. 9 (2005): 1100–1105, 10.1161/01.CIR.0000157153.30978.67. 15723973

[164] D. G. Wyse , A. L. Waldo , J. P. DiMarco , et al., “A Comparison of Rate Control and Rhythm Control in Patients With Atrial Fibrillation,” New England Journal of Medicine 347, no. 23 (2002): 1825–1833, 10.1056/NEJMoa021328. 12466506

[165] I. C. Van Gelder , V. E. Hagens , H. A. Bosker , et al., “A Comparison of Rate Control and Rhythm Control in Patients With Recurrent Persistent Atrial Fibrillation,” New England Journal of Medicine 347, no. 23 (2002): 1834–1840, 10.1056/NEJMoa021375. 12466507

[166] H. Calkins , M. R. Reynolds , P. Spector , et al., “Treatment of Atrial Fibrillation With Antiarrhythmic Drugs or Radiofrequency Ablation: Two Systematic Literature Reviews and Meta‐Analyses,” Circulation: Arrhythmia and Electrophysiology 2, no. 4 (2009): 349–361, 10.1161/CIRCEP.108.824789. 19808490

[167] M. P. Turakhia , M. Desai , H. Hedlin , et al., “Rationale and Design of a Large‐Scale, App‐Based Study to Identify Cardiac Arrhythmias Using a Smartwatch: The Apple Heart Study,” American Heart Journal 207 (2019): 66–75, 10.1016/j.ahj.2018.09.002. 30392584 PMC8099048

