# Supplementary material for: Concise Guidelines of the European Cardiac Arrhythmias Society (ECAS) on “Catheter Ablation of Atrial Fibrillation”
Source: J Cardiovasc Electrophysiol. 2025 Mar 4;36(5):1076–99. doi: 10.1111/jce.16561 (PMC12075922; doi:10.1111/jce.16561)
Supplement: Supplementary file 1 — Supporting information. [file JCE-36-1076-s002.doc]

**Supplementary Material of
Concise Guidelines of the European Cardiac Arrhythmias Society (ECAS) on
“Catheter Ablation of Atrial Fibrillation”**

^1^Riccardo Cappato, MD, (Chair), ^2^Samuel Levy, MD, (Co-chair), ^3^Rui Providencia, MD,
^1^Hussam Ali, MD, ^4^Andrey Ardashev, MD, ^5^Sergio Barra, MD, ^3^Antonio Creta, MD, ^6^Michal Farkowski, MD, ^7^Christian-Hendrik Heeger, MD, ^8^Prapa Kanagaratnam, PhD, FRCP, ^9^Thorsten Lewalter, MD, ^10^Silvia Magnani, MD, ^11^Richard Schilling, MD

^1^Arrhythmia and Electrophysiology Department, IRCCS MultiMedica, Milan, Italy,
^2^Marseille School of Medicine, Aix-Marseille University, Marseille, France
^3^Institute of Health Informatics Research, University College, and Barts Heart Centre, Barts Health NHS Trust, London, UK
^4^Department of Medicine, Feinberg School of Medicine, Northwestern University, Chicago, IL, USA,
^5^Department of Cardiology, Hospital da Luz Arrábida, V. N. Gaia, Portugal.
^6^Department of Cardiology, Ministry of Interior and Administration National Medical Institute, Warsaw, Pol
^7^University Heart Center Lübeck, Department of Rhythmology, University Hospital Schleswig-Holstein, and Asklepios Klinik Hamburg Altona, Clinical for Cardiology, Germany.
^8^Imperial College Healthcare NHS Trust, London, UK,
^9^Dept. of Cardiology, Hospital Munich South, Munich Germany and Univ. of Bonn, Bonn, Germany
^10^Cardiology Dept., San Paolo Hospital, Milan, Italy
^12^Barts Health NHS Trust and Welbeck Heart Health, London, UK

Internal reviewers:

1Douglas Packer, MD, (Chair), 2Stefan Willems, MD, 3Eli Ovsyshcher, MD,

4Leonardo Calò, MD, 5Feifan Ouyang, MD, 6Ali Oto, MD, 7Mark Estes, MD

^1^Mayo Clinic-St. Mary's Hospital, Rochester, Minnesota, USA

^2^Kardiologie Askaelepios Krankenhaus, Hamburg, Germany

^3^ Ben-Gurion University of the Negev, Beer Sheva, Israel

^4^Department of Cardiology, Casilino Hospital, Rome, Italy,

^5^State Key Laboratory of Cardiovascular Disease, Fuwai Hospital, Beijing, China

^6^Department of Cardiology, Memorial Hospital, Ankara, Turkey

^7^University of Pittsburgh Medical Center, Pittsburgh, Pennsylvania, USA.

# Table of supplement contents

1. **List of abbreviations 5**
2. **Narrative of ECAS GL production 6**
3. **Figure S1. Flowchart highlighting clinical conditions and catheter techniques**

**for which catheter ablation of AF is a class I recommendation with relevant**

**numbers from reference studies 12**

1. **Figure S2a. Flowchart highlighting clinical conditions for which catheter ablation**

**of AF is a class II recommendation (evidence for superiority of catheter ablation**

**versus control therapy or technologies/techniques), with relevant numbers from**

**reference studies 13**

1. **Figure S2b. Flowchart highlighting clinical conditions and catheter techniques**

**for which catheter ablation of AF is a class II recommendation with relevant**

**numbers from reference studies (evidence for similarity of efficacy and safety**

**profiles action of efficacy and safety profiles between comparative technologies or**

**techniques), with relevant numbers from reference studies 14**

1. **Figure S3a. Flowchart highlighting clinical conditions and catheter techniques**

**for which catheter ablation of AF is a class III recommendation based on**

**evidence-based documentation of harm from complimentary ablation strategies,**

**with relevant numbers from reference studies 15**

1. **Figure S3b. Flowchart highlighting clinical conditions and catheter techniques**

**in which catheter ablation of AF is a class III recommendation based on**

**evidence-based documentation of lack of benefit from complimentary ablation**

**strategies, with relevant numbers from reference studies 16**

1. **Figure S4. Flowchart highlighting clinical conditions for which catheter ablation**

**of AF is a class I recommendation showing AF type, technique or technology**

**adopted and outcomes on different levels 17**

1. **Figure S5a. Flowchart highlighting clinical conditions and catheter techniques**

**for which catheter ablation of AF is a class II recommendation (evidence for**

**superiority of catheter ablation versus control therapy or technologies/techniques)**

**showing AF type, technique or technology adopted and outcomes on different**

**levels 18**

1. **Figure S5b. Flowchart highlighting clinical conditions and catheter techniques for**

**which catheter ablation of AF is a class II recommendation (evidence for similarity**

**of action between comparative technologies or techniques) showing AF type,**

**technique or technology adopted and outcomes on different levels 19**

1. **Reference list of clinical studies representing the basis of the**

**recommendation scheme highlighted in figures S1, S2a, S2b, S3a and S3b 20**

1. **Figure S6. Forest plot of custom-built meta-analysis showing lack of efficacy**

**of supplementary linear lesion ablation versus PV isolation in catheter ablation**

**of paroxysmal and/or persistent AF 23**

1. **Reference list of literature contributions representing the basis of search for**

**selection of recommendation classes of efficacy in the guideline document 24**

1. **Table S1. Justifications adopted to qualify efficacy criteria for clinical or technical/technological conditions as class II recommendations for catheter**

**ablation of AF 55**

1. **Table S2 RCTs included in the custom-made meta-analysis elaboration for**

**fulfillment of class II recommendations. a. Study design and baseline**

**characteristics; b. Summary of findings; c. Sub-analyses and sensitivity analyses 60**

1. **Table S3. Main characteristics and outcomes of RCTs investigating the role of**

**supplementary linear lesion ablation for the treatment of AF 65**

1. **Table S4. Main characteristics and outcomes of RCTs investigating the role of**

**supplementary CFAE for the treatment of AF 71**

1. **Table S5. Main characteristics and outcomes of RCTs investigating the role of**

**GP ablation for the treatment of AF 76**

1. **Table S6. Comparison of recently published recommendation schemes of**

**guideline and consensus documents on catheter ablation of AF from the European**

**Society of Cardiology (ESC), and the American College of Cardiology (ACC)/**

**American Heart Association (AHA)/ Heart Rhythm Society /HRS, and from the**

**European Heart Rhythm Association/HRS/ Asian Pacific HRS, and the Latin**

**American HRS with the present ECAS guideline document 83**

1. **Table S7. Main characteristics of ongoing RCTs investigating the role of linear**

**ablation or LPW isolation for the treatment of AF 87**

1. **Table S8. Main characteristics of ongoing RCTs investigating the role of CFAE**

**ablation for the treatment of AF 88**

1. **Table S9. Main characteristics of ongoing RCTs investigating the role of LAA**

**isolation for the treatment of AF 88**

1. **Table S10. Efficacy outcomes reported in prospective studies enrolling at least**

**100 patients undergoing catheter ablation of paroxysmal AF (a), persistent AF**

**(b) and paroxysmal/persistent AF (c) 89**

1. **Table S11. Safety outcomes reported in prospective studies enrolling at least**

**100 patients undergoing catheter ablation of paroxysmal AF (a), persistent AF**

**(b) and paroxysmal/persistent AF (c) 93**

# List of abbreviations

**AAD, anti-arrhythmic drugs**

**ACT, activated clotting time**

**AF atrial fibrillation**

**CB, cryo-balloon**

**CFAE, complex fragmented atrial electrograms**

**CHF, congestive heart failure**

**CT, computerized tomography**

**CV, cardioversion**

**DC, direct current**

**ECAS, European Cardiac Arrhythmia Society**

**GPA, ganglionated plexi ablation**

**HFmrEF, heart failure with mildly reduced ejection fraction**

**HFpEF, heart failure with preserved ejection fraction**

**HFrEF, heart failure with reduced ejection fraction**

**ICD-9-CM, International Classification of Diseases-Ninth Revision-Clinical Modification**

**ICE, intra cardiac echocardiography**

**LA, left atrium**

**LAA, left atrial appendage**

**LPW, left posterior wall**

**LSVC, left superior vena cava**

**LVEF, left ventricular ejection fraction**

**NIS, Nationwide In-hospital Sample**

**NOAC, novel oral anticoagulants**

**RCT, randomized clinical trial**

**RF, radiofrequency**

**PF, pulsed field**

**SVC, superior vena cava**

**PV, pulmonary vein**

**TIA, transient ischemic attack**

**USP, United States Pharmacopeia**

**VKA, vitamin K antagonist**

**WACA, wide area circumferential ablation**

# Narrative of ECAS GL production

# *Recruitment of authors and internal reviewers and definition of scopes and roles*

# In September 2023, the Chairman of the GL document approached 13 experts in the field of AF, including members of ECAS, to explore their availability in the making of an original contribution addressing the field of “catheter ablation of AF”. The team was composed of expert electrophysiologists, clinical cardiologists and epidemiologists. Selected authors were required to sign a non-disclosure agreement to ensure confidentiality of the information shared during the kick-off meeting and thereafter. This initiative was taken with the aim of protecting the intellectual content of the document throughout the period until publication. After signing, each author was provided with an official letter of assignment by ECAS.

Following official assignment, a video conference was set by the Chairman to present the aim of the document. In brief, the document would represent a concise, simple and coordinate text to serve practitioners and patients in the practice of proposing and receiving catheter ablation of AF based on as robust methodology as possible. In keeping with the proposed scheme,

- the manuscript should be concise,
- indications should be corroborated by high-quality, evidence-based literature,
- sub-categorization based on expert position in favor or against a given recommendation, and level of evidence would be eliminated
- clinical flow-charts would be developed to translate GL recommendation into an easy, user-friendly picture to remember
- custom-built meta-analyses would be performed by selected members of the working team (RP, SB, MF, and AC) to provide, when feasible, intermediate-quality information enabling qualification for class II recommendation in the GL scheme

The list of topics included the following: introduction (RC, SL), rationale for AF ablation (RC, SL), classification of AF (AA), techniques and technologies in 2024 (CHH), recommendation scheme (class I, II, and III) for efficacy with explanatory figures (PK, RP, SM, AA, HA, SB, MF, AC, TL, SL, RC), production of an efficacy outcome table from large prospective studies for readers’ reference (SM, HA), recommendation scheme (class I, II, and III) for peri-procedural anticoagulation (TL, SB, AC, RC), procedure-related complications (AA, PK, TL, RC), production of a table summarizing ongoing RCTs on AF ablation (HA, RP, SB, MF, AC, SM) for readers’ reference, pending indications (RC, HA, SM), special conditions including heart failure (PK) or mortality (RS) and future directions (RC).

At that time, a supplementary text was also proposed to accommodate complimentary relevant information. This initially included: figures of the recommendation scheme for efficacy with complimentary relevant data in addition to that available in the main text figures; figures of forest plots from custom-built meta-analyses performed in preparation of the GL document; schematics of pre- and post-procedural anti-coagulation scheme to complement the information available in the main GL manuscript; tables on recommendation for efficacy enriched with justifications adopted to qualify for class II in the efficacy recommendation scheme; tables reporting study design, baseline characteristics, outcomes, sub-analyses and sensitivity analyses of clinical trials reviewed for custom-built meta-analyses or pending indications; and, tables of ongoing clinical trials investigating technique or technology effects on AF ablation efficacy and safety. Figures and tables would be associated with reference list, when applicable.

Originally, class II recommendation would rely on the contribution provided by:

- custom-built meta-analyses (RP, SB, MF, AC, HA, RC)
- sub-studies from RCTs published as separate manuscript (RP, SB, MF, AC, HA, SM, RC)
- large prospective observational studies (RP, SM, HA)
- large surveys or registries (RP, SM, HA)

Inclusion criteria for custom-built meta-analyses were outcome events in large RCTs that were reported as secondary endpoints and could be accurately retrieved from the original manuscripts. The results of custom-built meta-analyses lead to the production of two separate manuscripts^1,2^.

All authors accepted the invitation and were assigned a topic for initial drafting as outlined in brackets in the pertinent text.

At that time, a list of internal reviewers was identified and approved by the authors. The list included: Douglas Packer, Feifan Ouyang, Stefan Willems, Ali Oto, Eli Ovsysher, Leonardo Calò, and Mark Estes III.

*Pre-publication document on GLs*

Given the growing attention by several disciplinary bodies in the field of medicine^3,4^, the authors resolved to write a pre-publication manuscript addressing the methods used in preparation of the final GL document. The main tasks of such publication would be: 1) respond to the need for increased rigor and transparency in GL production; 2) reduce the size of the GL document, and thus make it of greater practical value to the readers. The pre-publication manuscript was published in March 2024^5^.

*Start and course of activities*

The first months were dedicated in parallel to develop the pre-publication manuscript on the methods in preparation of the final GL document and to achieve the outlined goals. Activities took place in sub-groups as delineated above and were regularly re-capitulated during meetings with the whole team. A file was generated for each author by the Chairman to preserve all correspondence taking place throughout the time period of each author’s activity.

Following the first output of custom-built meta-analyses in November 2024, it became evident that the quality of information from these studies was superior to that obtained from large, prospective observational studies and surveys or registries. Therefore, these two sources of class II recommendation foundation were abandoned.

Contributions from sub-groups generated the first draft in April 2024., which led to:1) pre-planned joint conference meeting for shared preparation of recommendation tables and figures; 2) remodulation of the original list of topics.

The development process followed multiple iterations with online meetings for discussion of indications and their respective class of recommendation. These meetings included all manuscript authors when planning the outline of the Guideline and voting final deliberations, or smaller teams when developing aspects of the guideline to present to the remaining Guideline development group. After formal presentation of the guideline recommendations, a discussion was held between members of the guideline development group. For cases of disagreement, a consensus was sought after hearing the points of both disagreeing sides in purposely designated meetings or e-mail communications. Agreement by consensus was reached on a subsequent meeting. The final text of the Guideline was circulated to all members of the guideline development committee, and all approved the final version of the document.

*Preparation of recommendation tables and figures*

Definition of rigorous qualifying criteria and elimination of arbitrary sub-classification and level of evidence categories rendered the selection of indications easier and judgment diversities among authors less probable. Conversely, these criteria required accurate review of the literature to ensure the most accurate match between methods and selected recommendations. Recommendation tables were generated for disciplines for which high- and intermediate-quality studies, as described above, were available. They included recommendation for efficacy of AF ablation, whether superior to control treatment or analyzed with respect of comparative techniques or technologies, and recommendation for peri-procedural anticoagulation.

Fulfillment of the class II recommendation scheme represented the biggest challenge as this category is the one least supported by high-quality scientific evidence. In the interest of accuracy, we elected to introduce a supplementary table (**Table S1, S2a, S2b S3a S3b, S4, S5a, S5b**) providing the justifications adopted to qualify clinical or technical/technological conditions as a class II recommendation in our scheme.

Controversy was encountered with respect to class III recommendations. Following an internal poll, it was decided that indications in this class would be those for which there is high-quality evidence for harm or inefficacy. The rationale behind this decision stemmed from the intention to discourage use of catheter ablation in identified conditions on large scale. **Tables S7, S8 and S9** have been added to provide readers with a list of ongoing trials that will likely help to shed light in these fields in the next future.

*Remodulation of the original list of topics*

Based on manuscript development, we remodulated the original list of topics as follows: introduction, truth in medicine and revisitation of the term recommendation, EP rationale on current AF ablation techniques, classification of AF type related to catheter ablation, recommendations on efficacy, pending indications, efficacy outcomes of catheter ablation of AF, periprocedural anticoagulation, complications of AF ablation, ongoing trials, emerging role of PF ablation, heart failure and AF ablation, peri-procedural mortality, mortality benefit of catheter ablation of AF, summary and future directions. This was made to best accommodate with the modifications of the original plan as obtained during manuscript evolution.

Supplementary custom-built tables were added to provide readers with efficacy (**Table S10**) and safety outcomes (**Table S11**) from sufficiently large prospective studies investigating the role of AF ablation in various clinical conditions. This effort was taken based on the general difficulty to compare outcome data in various studies. We thought that a global picture on relatively solid data may help the readers to orient in this field.

As we were writing this document, PF ablation continues to emerge as a promising alternative to RF and CB in patients with AF. Given the limited number of high-quality studies, we elected to dedicate a specific chapter to this discipline, which may help setting the future direction in the field.

Peri-procedural death continues to remain a catastrophic complication and is, to our view, not duly addressed in GL documents on AF ablation. Therefore, we elected to dedicate a chapter which summarizes the view of the authors in this topic.

*Compliance with WHO recommendations for GL production*

A checklist was downloaded from the WHO platform to finally check that all suggested relevant items were duly covered in the final manuscript.

**References**

1. Providencia R, Ali H, Barra S, Creta A, Kanagaratnam P, Schilling R, Farkowski MM, Cappato R. Catheter ablation for atrial fibrillation and impact on clinical outcomes. Nature Open 2024;00,oeae. doi.org/10.1093/ehjopen/oeae058
2. Providencia R, Ali H, Creta A, Barra S, Kanagaratnam P, Schilling R, Farkowski MM, Cappato R. Impact of catheter ablation of atrial fibrillation on the risk of stroke: A Meta-analysis. Nature Rev 2024;00,
3. Burgers JS, Fervers B, Haugh M, Brouwers M, Browman G, Phillip T, Cluzeau FA. International assessment of the quality of clinical practice guidelines in oncology using the Appraisal of Guidelines and Research and Evaluation Instrument. J Clin Oncol. 2004;22:2000-7.
4. Brouwers MC, Kho ME, Browman GP, Burgers JS, Cluzeau F, Feder G, Fervers B, Graham ID, Grimshaw J, Hanna SE, Littlejohns P, Makarski J, Zitzelsberger L, for the AGREE Next Steps Consortium. AGREE II: Advancing guideline development, reporting and evaluation in healthcare. CMAJ 2010;182:E839-842.
5. Cappato R, Levy S, Providencia R, Ali H, Ardashev A, Barra S, Creta A, Farkowski M, Heeger CH, Kanagaratnam P, Lewalter T, Magnani S, Shah D, Schilling R. Concise guidelines of the European Cardiac Arrhythmias Society (ECAS) on "catheter ablation of atrial fibrillation": A prepublication of the methods in preparation of the final guidelines document. J Cardiovasc Electrophysiol. 2024 May 12. doi: 10.1111/jce.16254. Epub ahead of print. PMID: 3873615

# Figure S1. Flowchart highlighting clinical conditions and catheter techniques for which catheter ablation of AF is a class I recommendation with relevant numbers from reference studies


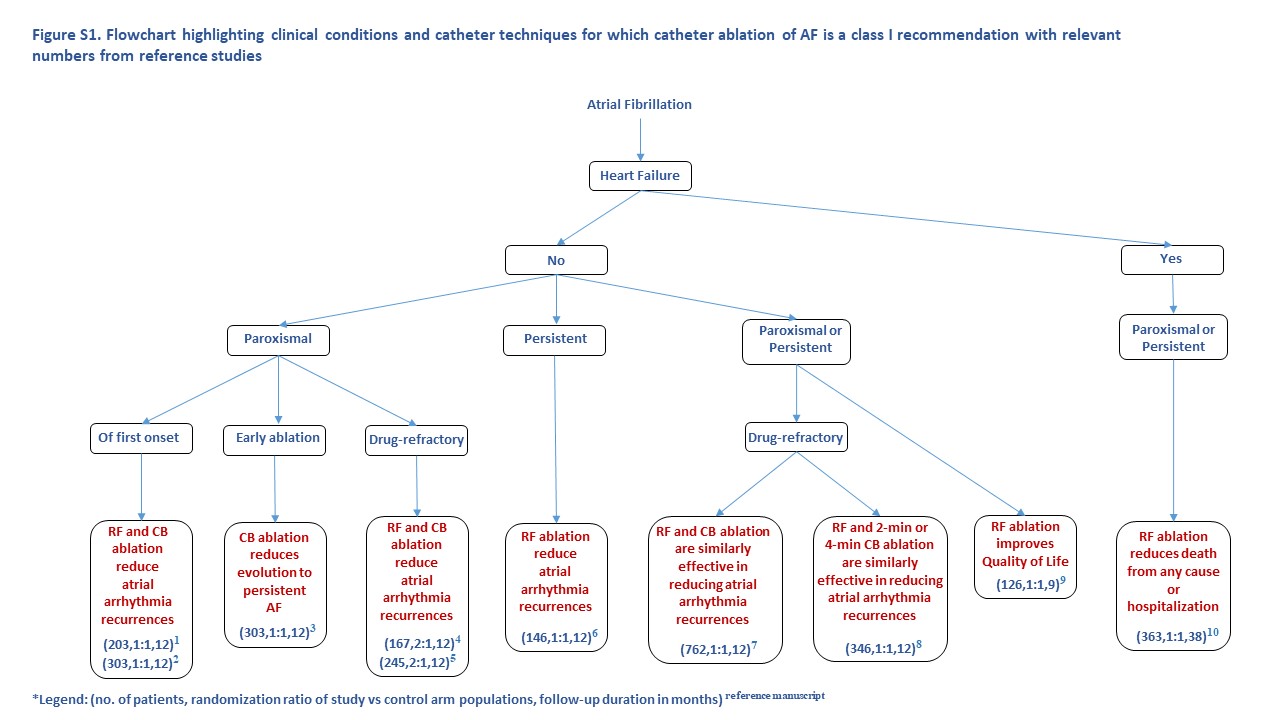


# Figure S2a. Flowchart highlighting clinical conditions for which catheter ablation of AF is a class II recommendation (evidence for superiority of catheter ablation versus control therapy or technologies7techniques), with relevant numbers from references studies


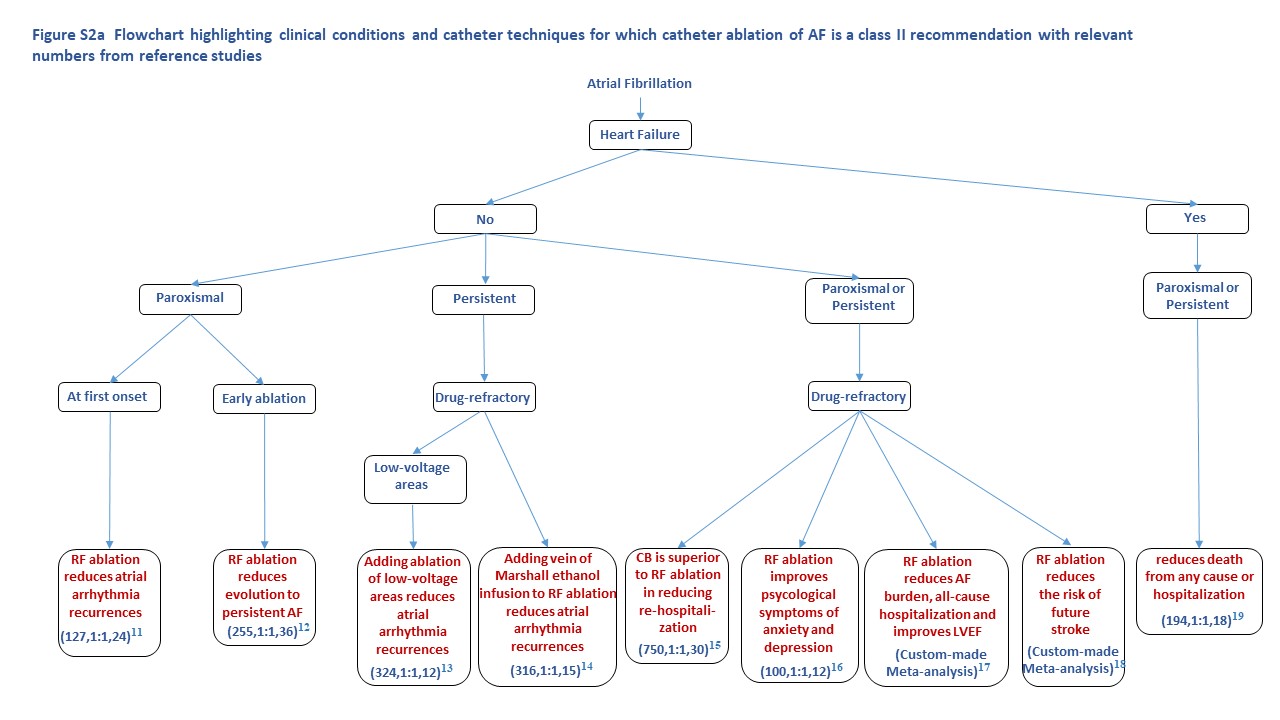


# Figure S2b. Flowchart highlighting clinical conditions and catheter techniques for which catheter ablation of AF is a class II recommendation (evidence for similarity of efficacy and safety profiles action of efficacy and safety profiles between comparative technologies or techniques) with relevant numbers from reference studies


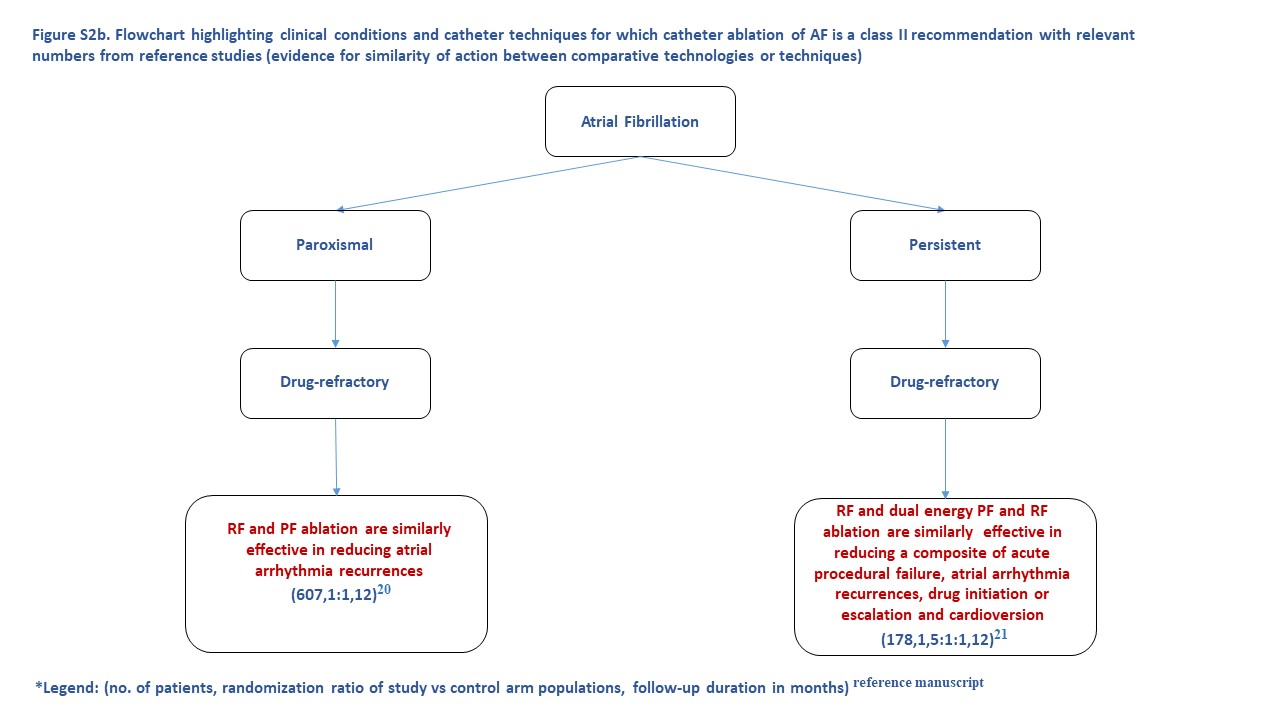


# Figure S3a. Flowchart highlighting clinical conditions and catheter techniques for which catheter ablation of AF is a class III recommendation based on evidence-based documentation of harm from complimentary ablation strategies, with relevant numbers from reference studies


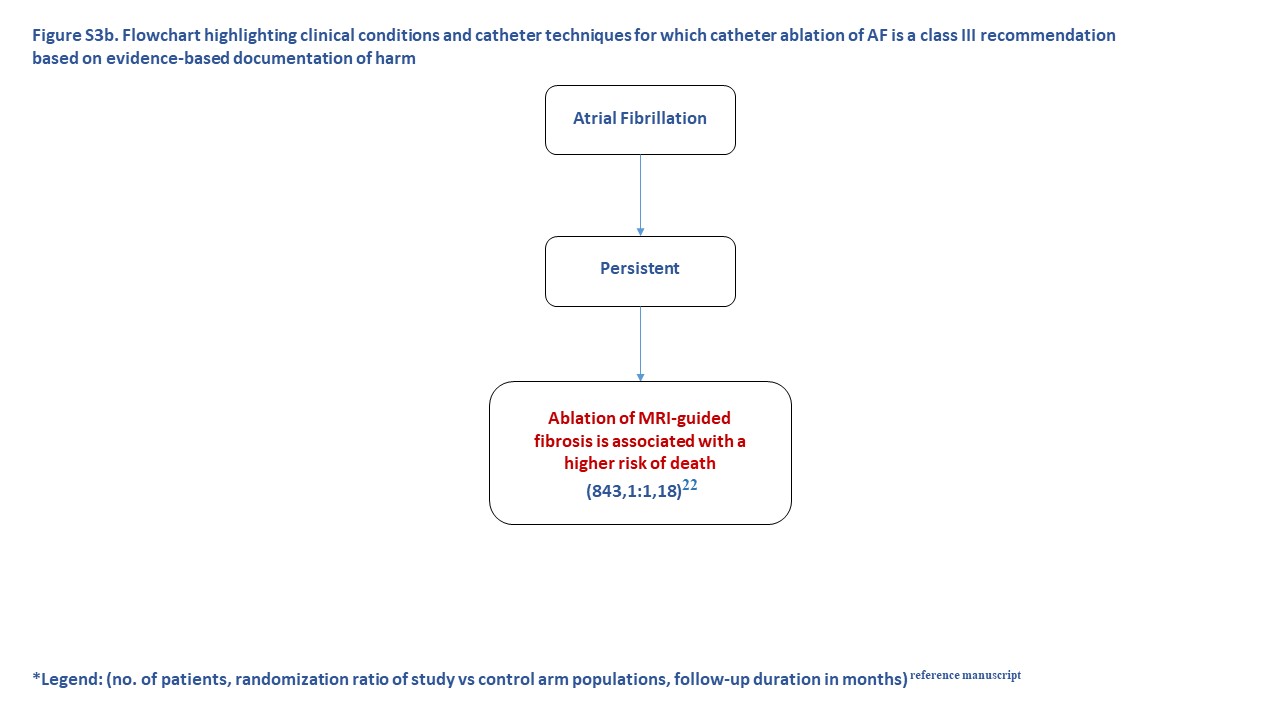


# Figure S3b. Flowchart highlighting clinical conditions and catheter techniques in which catheter ablation of AF is a class III recommendation based on evidence-based documentation of lack of benefit from complimentary ablation strategies, with relevant numbers from reference studies


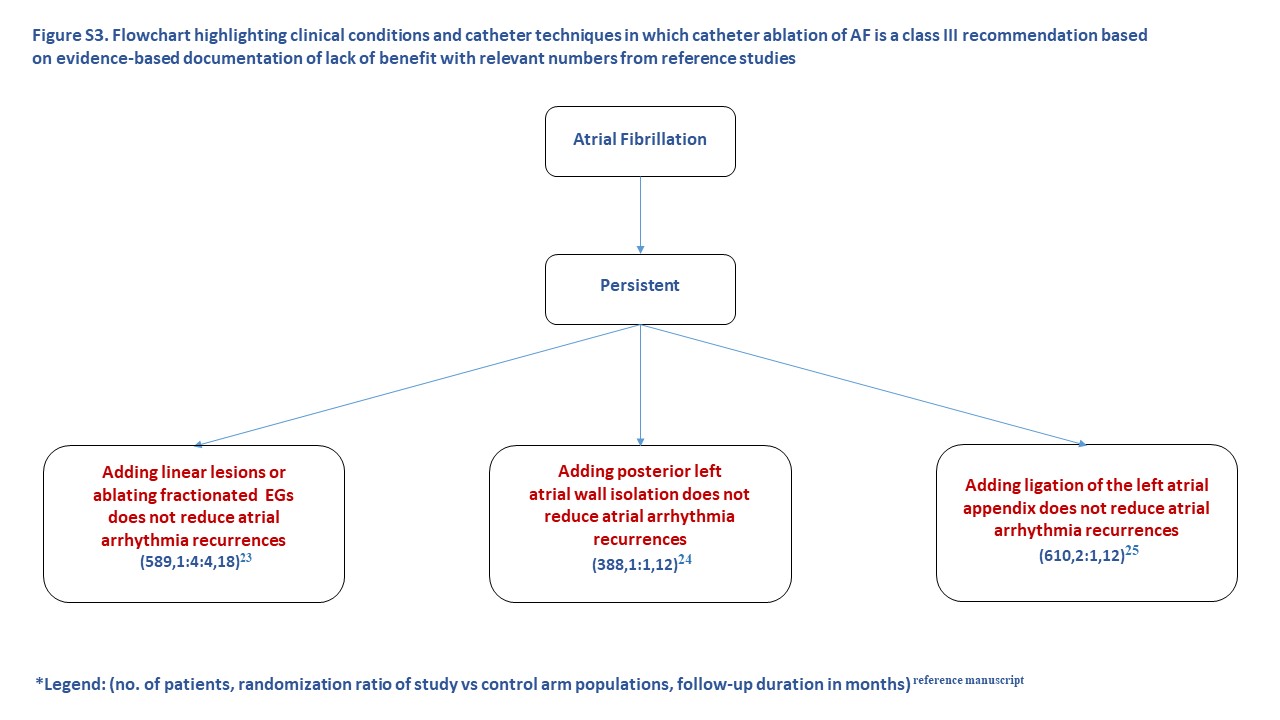


# Figure S4. Flowchart highlighting clinical conditions for which catheter ablation of AF is a class I recommendation showing AF type, technique or technology adopted and outcomes on different levels


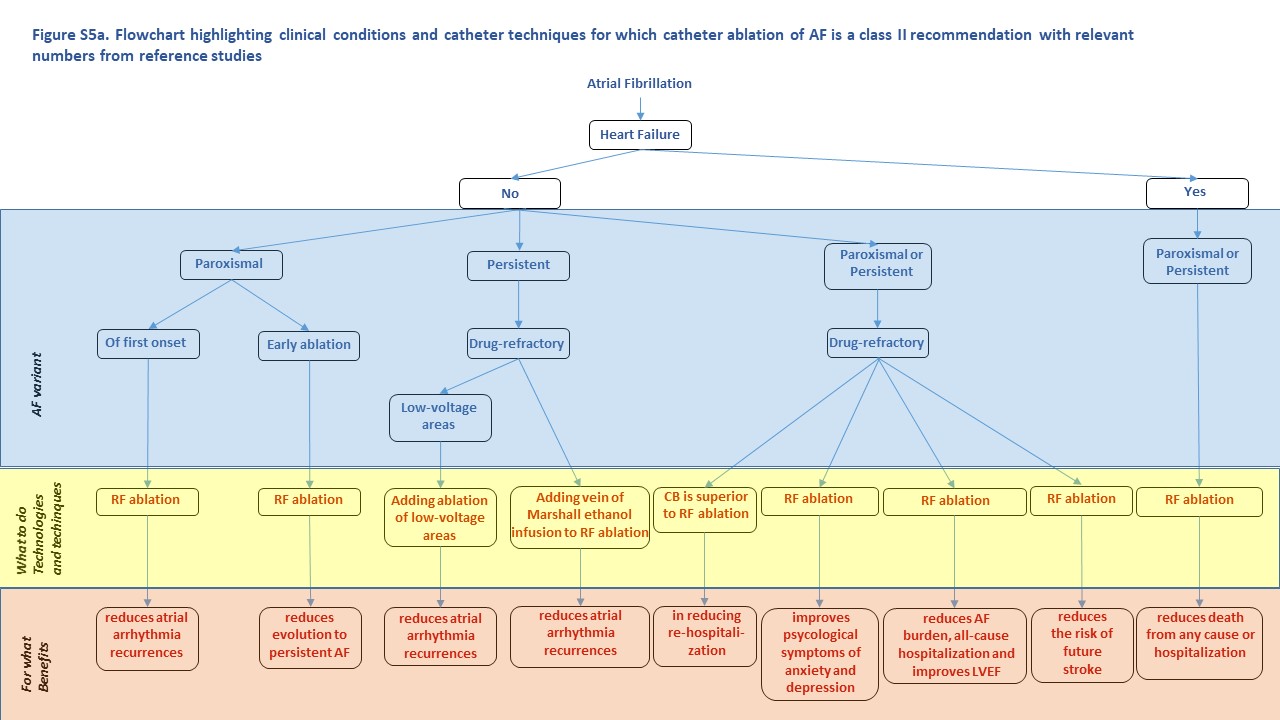


# Figure S5a. Flowchart highlighting clinical conditions and catheter techniques for which catheter ablation of AF is a class II recommendation (evidence for superiority of catheter ablation versus control therapy or technologies/techniques) showing AF type, technique or technology adopted and outcomes on different levels


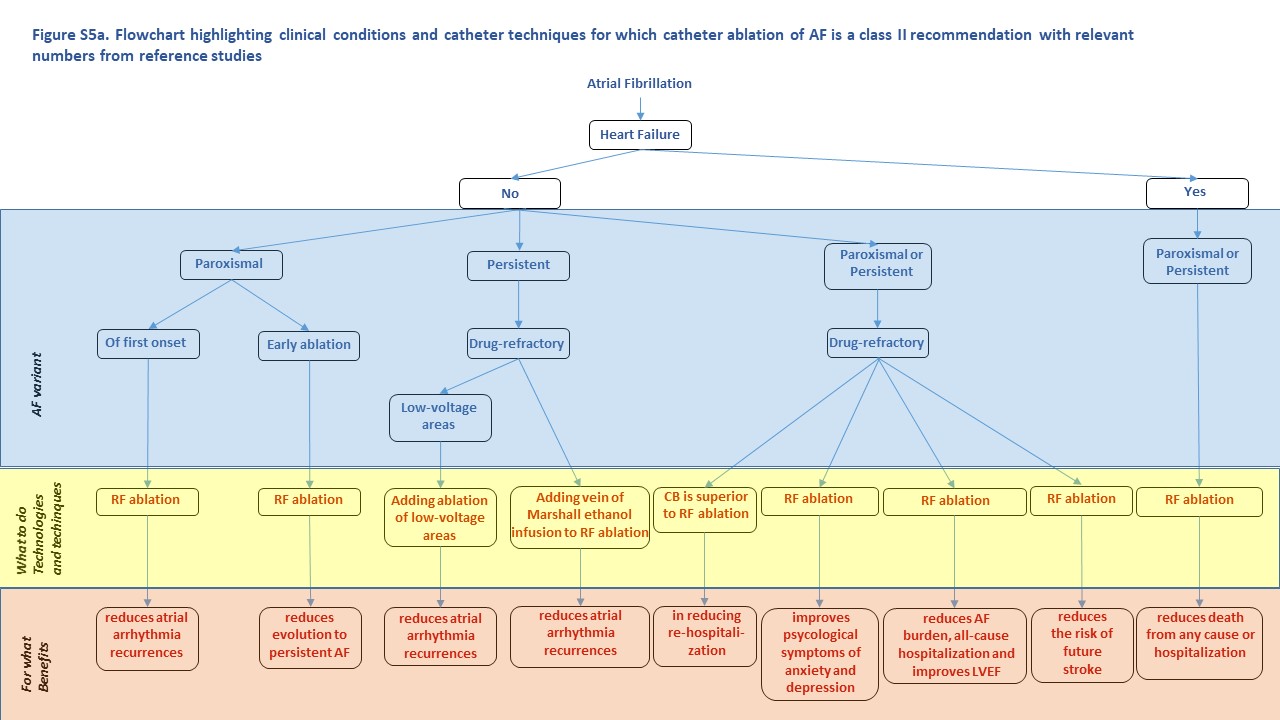


# Figure S5b. Flowchart highlighting clinical conditions and catheter techniques for which catheter ablation of AF is a class II recommendation (evidence for similarity of action between comparative technologies or techniques) showing AF type , technique or technology adopted and outcomes on different levels


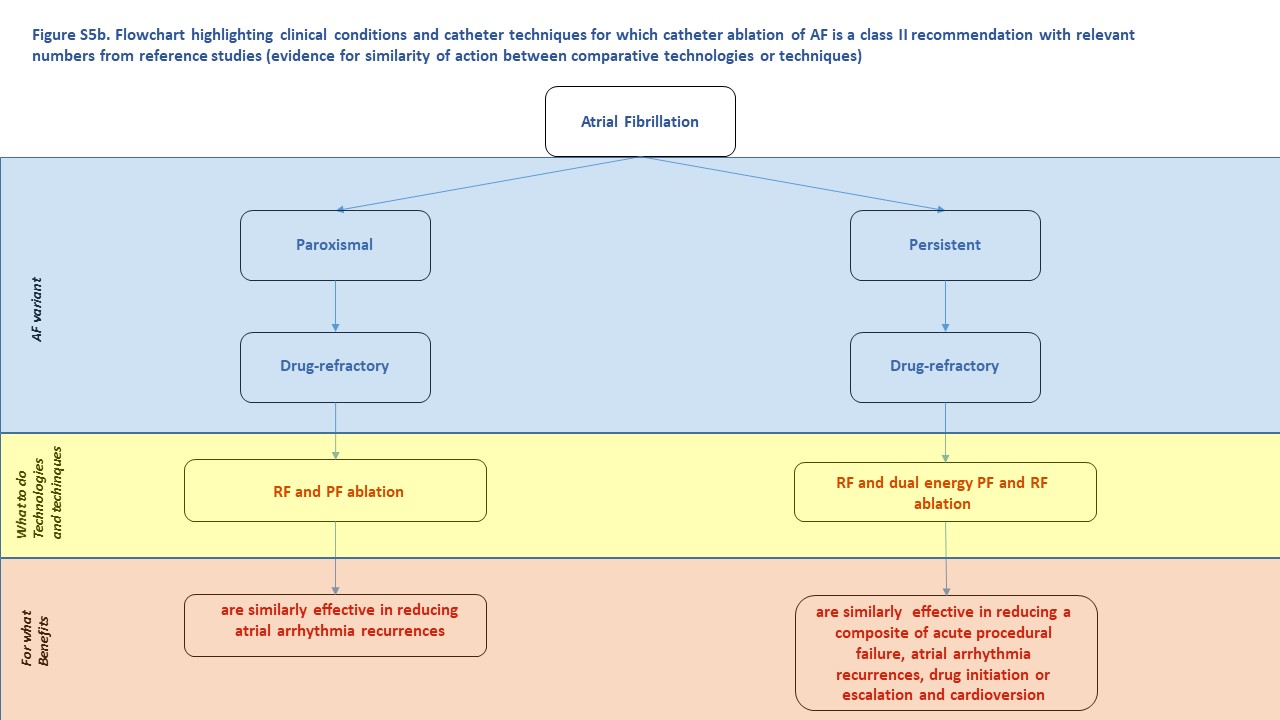


# Reference list of clinical studies representing the basis of the recommendation scheme as highlighted in figures S1, S2a, S2b, S3a and S3b

1. Andrade JG, Wazni OM, Kuniss M, et al. Cryoballoon Ablation as Initial Treatment for Atrial Fibrillation: JACC State-of-the-Art Review. J Am Coll Cardiol. 2021;78(9):914-930. doi:10.1016/j.jacc.2021.06.038

2. Wazni OM, Dandamudi G, Sood N, et al. Cryoballoon Ablation as Initial Therapy for Atrial Fibrillation. N Engl J Med. 2021;384(4):316-324. doi:10.1056/NEJMoa2029554

3. Andrade JG, Deyell MW, Macle L, et al. Progression of Atrial Fibrillation after Cryoablation or Drug Therapy. New England Journal of Medicine. 2023;388(2):105-116. doi:10.1056/NEJMoa2212540

4. Wilber DJ, Pappone C, Neuzil P, et al. Comparison of antiarrhythmic drug therapy and radiofrequency catheter ablation in patients with paroxysmal atrial fibrillation: a randomized controlled trial. JAMA. 2010;303(4):333-340. doi:10.1001/jama.2009.2029

5. Packer DL, Kowal RC, Wheelan KR, et al. Cryoballoon ablation of pulmonary veins for paroxysmal atrial fibrillation: first results of the North American Arctic Front (STOP AF) pivotal trial. J Am Coll Cardiol. 2013;61(16):1713-1723. doi:10.1016/j.jacc.2012.11.064

6. Mont L, Bisbal F, Hernández-Madrid A, et al. Catheter ablation vs. antiarrhythmic drug treatment of persistent atrial fibrillation: a multicentre, randomized, controlled trial (SARA study). Eur Heart J. 2014;35(8):501-507. doi:10.1093/eurheartj/eht457

7. Kuck KH, Brugada J, Fürnkranz A, et al. Cryoballoon or Radiofrequency Ablation for Paroxysmal Atrial Fibrillation. N Engl J Med. 2016;374(23):2235-2245. doi:10.1056/NEJMoa1602014

8. Andrade JG, Champagne J, Dubuc M, et al. Cryoballoon or Radiofrequency Ablation for Atrial Fibrillation Assessed by Continuous Monitoring: A Randomized Clinical Trial. Circulation. 2019;140(22):1779-1788. doi:10.1161/CIRCULATIONAHA.119.042622

9. Dulai R, Sulke N, Freemantle N, et al. Pulmonary Vein Isolation vs Sham Intervention in Symptomatic Atrial Fibrillation: The SHAM-PVI Randomized Clinical Trial. JAMA. Published online September 2, 2024. doi:10.1001/jama.2024.17921

10. Marrouche NF, Brachmann J, Andresen D, et al. Catheter Ablation for Atrial Fibrillation with Heart Failure. N Engl J Med. 2018;378(5):417-427. doi:10.1056/NEJMoa1707855

11. Morillo CA, Verma A, Connolly SJ, et al. Radiofrequency ablation vs antiarrhythmic drugs as first-line treatment of paroxysmal atrial fibrillation (RAAFT-2): a randomized trial. JAMA. 2014;311(7):692-700. doi:10.1001/jama.2014.467

12. Kuck KH, Lebedev DS, Mikhaylov EN, et al. Catheter ablation or medical therapy to delay progression of atrial fibrillation: the randomized controlled atrial fibrillation progression trial (ATTEST). Europace. 2021;23(3):362-369. doi:10.1093/europace/euaa298

13. Huo Y, Gaspar T, Schönbauer R, et al. Low-Voltage Myocardium-Guided Ablation Trial of Persistent Atrial Fibrillation. NEJM Evid. 2022;1(11):EVIDoa2200141. doi:10.1056/EVIDoa2200141

14. Valderrábano M, Peterson LE, Swarup V, et al. Effect of Catheter Ablation With Vein of Marshall Ethanol Infusion vs Catheter Ablation Alone on Persistent Atrial Fibrillation: The VENUS Randomized Clinical Trial. JAMA. 2020;324(16):1620-1628. doi:10.1001/jama.2020.16195

15. Kuck KH, Fürnkranz A, Chun KRJ, et al. Cryoballoon or radiofrequency ablation for symptomatic paroxysmal atrial fibrillation: reintervention, rehospitalization, and quality-of-life outcomes in the FIRE AND ICE trial. Eur Heart J. 2016;37(38):2858-2865. doi:10.1093/eurheartj/ehw285

15. Al-Kaisey AM, Parameswaran R, Bryant C, et al. Atrial Fibrillation Catheter Ablation vs Medical Therapy and Psychological Distress: A Randomized Clinical Trial. JAMA. 2023;330(10):925-933. doi:10.1001/jama.2023.14685

17. Providencia R, Ali H, Barra S, Creta A, Kukendra-Rajah K, Kanagaratnam P, Farkowski M, Cappato R. Ablation of atrial fibrillation and risk of stroke: a Meta-analysis. Heart Rhythm. 2025:22:

18. Providencia R, Ali H, Creta A, et al. Catheter ablation for atrial fibrillation and impact on clinical outcomes. Eur Heart J Open. 2024;4(4):oeae058. doi:10.1093/ehjopen/oeae058

19. Sohns C, Fox H, Marrouche NF, et al. Catheter Ablation in End-Stage Heart Failure with Atrial Fibrillation. N Engl J Med. 2023;389(15):1380-1389. doi:10.1056/NEJMoa2306037

20. Reddy, V. Y., Gerstenfeld, E. P., Natale, A., Whang, W., Cuoco, F. A., Patel, C., Mountantonakis, S. E., Gibson, D. N., Harding, J. D., Ellis, C. R., Ellenbogen, K. A., DeLurgio, D. B., Osorio, J., Achyutha, A. B., Schneider, C. W., Mugglin, A. S., Albrecht, E. M., Stein, K. M., Lehmann, J. W., & Mansour, M. (2023). Pulsed Field or Conventional Thermal Ablation for Paroxysmal Atrial Fibrillation. New England Journal of Medicine, 389(18), 1660–1671. https://doi.org/10.1056/nejmoa2307291

21. Anter E, Mansour M, Nair DG, et al. Dual-energy lattice-tip ablation system for persistent atrial fibrillation: a randomized trial. Nat Med. 2024;30(8):2303-2310. doi:10.1038/s41591-024-03022-6

22. Marrouche NF, Wazni O, McGann C, et al. Effect of MRI-Guided Fibrosis Ablation vs Conventional Catheter Ablation on Atrial Arrhythmia Recurrence in Patients With Persistent Atrial Fibrillation: The DECAAF II Randomized Clinical Trial. JAMA. 2022;327(23):2296-2305. doi:10.1001/jama.2022.8831

23. Verma A, Jiang C yang, Betts TR, et al. Approaches to catheter ablation for persistent atrial fibrillation. N Engl J Med. 2015;372(19):1812-1822. doi:10.1056/NEJMoa1408288

24. Kistler PM, Chieng D, Sugumar H, et al. Effect of Catheter Ablation Using Pulmonary Vein Isolation With vs Without Posterior Left Atrial Wall Isolation on Atrial Arrhythmia Recurrence in Patients With Persistent Atrial Fibrillation: The CAPLA Randomized Clinical Trial. JAMA. 2023;329(2):127-135. doi:10.1001/jama.2022.23722

25. Lakkireddy DR, Wilber DJ, Mittal S, et al. Pulmonary Vein Isolation With or Without Left Atrial Appendage Ligation in Atrial Fibrillation: The aMAZE Randomized Clinical Trial. JAMA. 2024;331(13):1099-1108. doi:10.1001/jama.2024.3026

# Figure S6. Forest plot of custom-built meta-analysis showing lack of efficacy of supplementary linear lesion ablation versus PV isolation in catheter ablation of paroxysmal and/or persistent AF


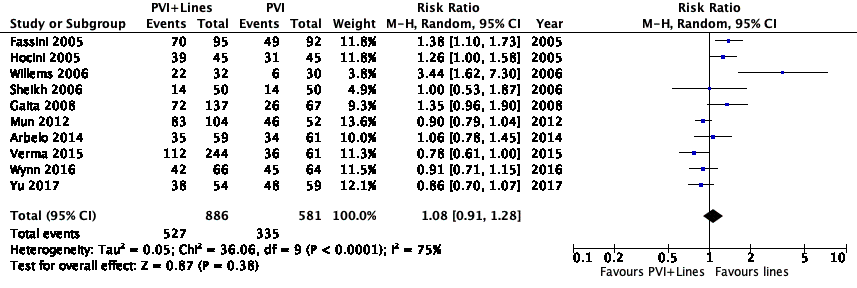


# Reference list of literature contributions representing the basis of search for selection of recommendation classes of efficacy in the guideline document

1. Dong Y, Zhao D, Chen X, Shi L, Chen Q, Zhang H, Yu Y, Ullah I, Kojodjojo P, Zhang F. Role of electroanatomical mapping guided superior vena cava isolation in paroxysmal atrial fibrillation patients without provoked superior vena cava trigger: A randomized controlled study. Europace. 2024 Feb 2:euae039. doi: 10.1093/europace/euae039. Epub ahead of print. PMID: 38306471.
2. Zeng L, Chen S, Zeng R, Hu H, Yang Q, Cui K, Chen Q, Fu H, Pu X. CLOSE protocol versus lower ablation index value for paroxysmal atrial fibrillation: A randomized noninferior clinical trial. J Cardiovasc Electrophysiol. 2024 Jan 28. doi: 10.1111/jce.16194. Epub ahead of print. PMID: 38282257.
3. Lin CY, Lin YJ, Higa S, Tsai WC, Lo MT, Chiang CH, Chang SL, Lo LW, Hu YF, Chao TF, Chung FP, Liao JN, Chang TY, Lin C, Tuan TC, Kuo L, Wu CI, Liu CM, Liu SH, Kuo MJ, Liao YC, Chuang CM, Chen YY, Hsieh YC, Chen SA. Catheter Ablation With Morphologic Repetitiveness Mapping for Persistent Atrial Fibrillation. JAMA Netw Open. 2023 Nov 1;6(11):e2344535. doi: 10.1001/jamanetworkopen.2023.44535. PMID: 37991761; PMCID: PMC10665974.
4. Joza J, Nair GM, Birnie DH, Nery PB, Redpath CJ, Sarrazin JF, Champagne J, Bernick J, Wells GA, Essebag V; other members of the AWARE Study Consortium (Augmented Wide Area Circumferential Catheter Ablation for Reduction of Atrial Fibrillation Recurrence). High-power short-duration versus low-power long-duration ablation for pulmonary vein isolation: A substudy of the AWARE randomized controlled trial. J Cardiovasc Electrophysiol. 2024 Jan;35(1):136-145. doi: 10.1111/jce.16123. Epub 2023 Nov 21. PMID: 37990448.
5. Xu C, Zhang F, Chen W, Chen N, Zhang Y, Zhu X, Liu Y, Jiang W, Wu S, Liu X, Qin M. Adjunctive Right Atrial Ablation for Persistent Atrial Fibrillation with Right Atrial Enlargement: A Pilot Study. Can J Cardiol. 2023 Nov 17:S0828-282X(23)01934-7. doi: 10.1016/j.cjca.2023.11.013. Epub ahead of print. PMID: 37981042.
6. Schrickel JW, Beiert T, Linhart M, Luetkens JA, Schmitz J, Schmid M, Hindricks G, Arentz T, Stellbrink C, Deneke T, Bogossian H, Sause A, Steven D, Gonska BD, Rudic B, Lewalter T, Zabel M, Geisler T, Schumacher B, Jung W, Kleemann T, Luik A, Veltmann C, Coenen M, Nickenig G. Prevention of cerebral thromboembolism by oral anticoagulation with dabigatran after pulmonary vein isolation for atrial fibrillation: the ODIn-AF trial. Clin Res Cardiol. 2023 Nov 3. doi: 10.1007/s00392-023-02319-9. Epub ahead of print. PMID: 37921923.
7. Demolder A, O'Neill L, El Haddad M, Scherr D, Vijgen J, Wolf M, Berte B, Bisbal F, Johannessen A, Rivero-Ayerza M, De Potter T, De Becker B, Polain de Waroux JL, Knecht S, Tavernier R, Duytschaever M. No Effect of Continued Antiarrhythmic Drug Treatment on Top of Optimized Pulmonary Vein Isolation in Patients With Persistent Atrial Fibrillation: Results From the POWDER-AF2 Trial. Circ Arrhythm Electrophysiol. 2023 Nov;16(11):e012043. doi: 10.1161/CIRCEP.123.012043. Epub 2023 Nov 3. PMID: 37921006.
8. Weiss R, Osorio J, Nair D, Aguinaga L, Arabia L, Alcivar D, Al-Ahmad A, Tomassoni G, Kahaly O, Mehta R, Ward C, Holmes B, Patel D, Killu AM, Munger T, Essandoh M, Houmsse M, Rajendra A, Morales G, Hummel JD, Balasubramanian G, Daoud EG. EsophAguS Deviation During RadiofrequencY Ablation of Atrial Fibrillation: The EASY AF Trial. JACC Clin Electrophysiol. 2024 Jan;10(1):68-78. doi: 10.1016/j.jacep.2023.09.004. Epub 2023 Oct 25. PMID: 37897465.
9. William J, Chieng D, Sugumar H, Ling LH, Segan L, Crowley R, Al-Kaisey A, Hawson J, Prabhu S, Voskoboinik A, Wong G, Morton JB, Lee G, McLellan AJ, Wong M, Pathak RK, Sterns L, Ginks M, Reid CM, Sanders P, Kalman JM, Kistler PM. The Role of Posterior Wall Isolation in Catheter Ablation for Persistent Atrial Fibrillation and Systolic Heart Failure: A Secondary Analysis of a Randomized Clinical Trial. JAMA Cardiol. 2023 Nov 1;8(11):1077-1082. doi: 10.1001/jamacardio.2023.3208. PMID: 37755920; PMCID: PMC10534992.
10. Al-Kaisey AM, Parameswaran R, Bryant C, Anderson RD, Hawson J, Chieng D, Segan L, Voskoboinik A, Sugumar H, Wong GR, Finch S, Joseph SA, McLellan A, Ling LH, Morton J, Sparks P, Sanders P, Lee G, Kistler PM, Kalman JM. Atrial Fibrillation Catheter Ablation vs Medical Therapy and Psychological Distress: A Randomized Clinical Trial. JAMA. 2023 Sep 12;330(10):925-933. doi: 10.1001/jama.2023.14685. PMID: 37698564; PMCID: PMC10498333.
11. Reddy VY, Gerstenfeld EP, Natale A, Whang W, Cuoco FA, Patel C, Mountantonakis SE, Gibson DN, Harding JD, Ellis CR, Ellenbogen KA, DeLurgio DB, Osorio J, Achyutha AB, Schneider CW, Mugglin AS, Albrecht EM, Stein KM, Lehmann JW, Mansour M; ADVENT Investigators. Pulsed Field or Conventional Thermal Ablation for Paroxysmal Atrial Fibrillation. N Engl J Med. 2023 Nov 2;389(18):1660-1671. doi: 10.1056/NEJMoa2307291. Epub 2023 Aug 27. PMID: 37634148.
12. Andrade JG, Deyell MW, Khairy P, Champagne J, Leong-Sit P, Novak P, Sterns L, Roux JF, Sapp J, Bennett R, Bennett M, Hawkins N, Sanders P, Macle L. Atrial fibrillation progression after cryoablation vs. radiofrequency ablation: the CIRCA-DOSE trial. Eur Heart J. 2024 Feb 16;45(7):510-518. doi: 10.1093/eurheartj/ehad572. PMID: 37624879.
13. Chen H, Li C, Han B, Xiao F, Yi F, Wei Y, Jiang C, Zou C, Shi L, Ma W, Wang W, Wang Y, Du H, Chen L, Chen M; STABLE-SR-III Investigators. Circumferential Pulmonary Vein Isolation With vs Without Additional Low-Voltage-Area Ablation in Older Patients With Paroxysmal Atrial Fibrillation: A Randomized Clinical Trial. JAMA Cardiol. 2023 Aug 1;8(8):765-772. doi: 10.1001/jamacardio.2023.1749. PMID: 37378966; PMCID: PMC10308299.
14. Sohns C, Fox H, Marrouche NF, Crijns HJGM, Costard-Jaeckle A, Bergau L, Hindricks G, Dagres N, Sossalla S, Schramm R, Fink T, El Hamriti M, Moersdorf M, Sciacca V, Konietschke F, Rudolph V, Gummert J, Tijssen JGP, Sommer P; CASTLE HTx Investigators. Catheter Ablation in End-Stage Heart Failure with Atrial Fibrillation. N Engl J Med. 2023 Oct 12;389(15):1380-1389. doi: 10.1056/NEJMoa2306037. Epub 2023 Aug 27. PMID: 37634135.
15. Mililis P, Kariki O, Saplaouras A, Bazoukis G, Dragasis S, Patsiotis IG, Batsouli A, Vlachos K, Letsas KP, Efremidis M. Radiofrequency versus cryoballoon catheter ablation in patients with persistent atrial fibrillation: A randomized trial. J Cardiovasc Electrophysiol. 2023 Jul;34(7):1523-1528. doi: 10.1111/jce.15965. Epub 2023 Jun 9. PMID: 37293822.
16. Al-Kaisey AM, Parameswaran R, Bryant C, Anderson RD, Hawson J, Chieng D, Voskoboinik A, Sugumar H, West D, Azzopardi S, Finch S, Wong G, Joseph SA, McLellan A, Ling LH, Sanders P, Lee G, Kistler PM, Kalman JM. Impact of Catheter Ablation on Cognitive Function in Atrial Fibrillation: A Randomized Control Trial. JACC Clin Electrophysiol. 2023 Jul;9(7 Pt 2):1024-1034. doi: 10.1016/j.jacep.2023.02.020. Epub 2023 May 24. PMID: 37227345.
17. Kalman JM, Al-Kaisey AM, Parameswaran R, Hawson J, Anderson RD, Lim M, Chieng D, Joseph SA, McLellan A, Morton JB, Sparks PB, Lee G, Sanders P, Kistler PM. Impact of early vs. delayed atrial fibrillation catheter ablation on atrial arrhythmia recurrences. Eur Heart J. 2023 Jul 14;44(27):2447-2454. doi: 10.1093/eurheartj/ehad247. PMID: 37062010.
18. O'Neill L, El Haddad M, Berte B, Kobza R, Hilfiker G, Scherr D, Manninger M, Wijnmaalen AP, Trines SA, Wielandts JY, Gillis K, Lycke M, De Becker B, Tavernier R, Le Polain De Waroux JB, Knecht S, Duytschaever M. Very High-Power Ablation for Contiguous Pulmonary Vein Isolation: Results From the Randomized POWER PLUS Trial. JACC Clin Electrophysiol. 2023 Apr;9(4):511-522. doi: 10.1016/j.jacep.2022.10.039. Epub 2023 Jan 18. PMID: 36752467.
19. Chieng D, Sugumar H, Segan L, Tan C, Vizi D, Nanayakkara S, Al-Kaisey A, Hawson J, Prabhu S, Voskoboinik A, Finch S, Morton JB, Lee G, Mariani J, La Gerche A, Taylor AJ, Howden E, Kistler PM, Kalman JM, Kaye DM, Ling LH. Atrial Fibrillation Ablation for Heart Failure With Preserved Ejection Fraction: A Randomized Controlled Trial. JACC Heart Fail. 2023 Jun;11(6):646-658. doi: 10.1016/j.jchf.2023.01.008. Epub 2023 Mar 1. PMID: 36868916.
20. Li K, Xu C, Zhu X, Wang X, Ye P, Jiang W, Wu S, Xu K, Li X, Wang Y, Zheng Q, Wang Y, Leng L, Zhang Z, Han B, Zhang Y, Qin M, Liu X. Multi-centre, prospective randomized comparison of three different substrate ablation strategies for persistent atrial fibrillation. Europace. 2023 May 19;25(5):euad090. doi: 10.1093/europace/euad090. PMID: 37050858; PMCID: PMC10228617.
21. Nair GM, Birnie DH, Nery PB, Redpath CJ, Sarrazin JF, Roux JF, Parkash R, Bernier M, Sterns LD, Sapp J, Novak P, Veenhuyzen G, Morillo CA, Singh SM, Sadek MM, Golian M, Klein A, Sturmer M, Chauhan VS, Angaran P, Green MS, Bernick J, Wells GA, Essebag V. Standard vs Augmented Ablation of Paroxysmal Atrial Fibrillation for Reduction of Atrial Fibrillation Recurrence: The AWARE Randomized Clinical Trial. JAMA Cardiol. 2023 May 1;8(5):475-483. doi: 10.1001/jamacardio.2023.0212. PMID: 36947030; PMCID: PMC10034661.
22. Kanagaratnam P, McCready J, Tayebjee M, Shepherd E, Sasikaran T, Todd D, Johnson N, Kyriacou A, Hayat S, Hobson NA, Mann I, Balasubramaniam R, Whinnett Z, Earley M, Petkar S, Veasey R, Kirubakaran S, Coyle C, Kim MY, Lim PB, O'Neill J, Davies DW, Peters NS, Babalis D, Linton N, Falaschetti E, Tanner M, Shah J, Poulter N. Ablation versus anti-arrhythmic therapy for reducing all hospital episodes from recurrent atrial fibrillation: a prospective, randomized, multi-centre, open label trial. Europace. 2023 Mar 30;25(3):863-872. doi: 10.1093/europace/euac253. PMID: 36576323; PMCID: PMC10062288.castle
23. Kistler PM, Chieng D, Sugumar H, Ling LH, Segan L, Azzopardi S, Al-Kaisey A, Parameswaran R, Anderson RD, Hawson J, Prabhu S, Voskoboinik A, Wong G, Morton JB, Pathik B, McLellan AJ, Lee G, Wong M, Finch S, Pathak RK, Raja DC, Sterns L, Ginks M, Reid CM, Sanders P, Kalman JM. Effect of Catheter Ablation Using Pulmonary Vein Isolation With vs Without Posterior Left Atrial Wall Isolation on Atrial Arrhythmia Recurrence in Patients With Persistent Atrial Fibrillation: The CAPLA Randomized Clinical Trial. JAMA. 2023 Jan 10;329(2):127-135. doi: 10.1001/jama.2022.23722. PMID: 36625809; PMCID: PMC9856612.
24. Andrade JG, Deyell MW, Macle L, Wells GA, Bennett M, Essebag V, Champagne J, Roux JF, Yung D, Skanes A, Khaykin Y, Morillo C, Jolly U, Novak P, Lockwood E, Amit G, Angaran P, Sapp J, Wardell S, Lauck S, Cadrin-Tourigny J, Kochhäuser S, Verma A; EARLY-AF Investigators. Progression of Atrial Fibrillation after Cryoablation or Drug Therapy. N Engl J Med. 2023 Jan 12;388(2):105-116. doi: 10.1056/NEJMoa2212540. Epub 2022 Nov 7. PMID: 36342178.
25. Kanagaratnam P, McCready J, Tayebjee M, Shepherd E, Sasikaran T, Todd D, Johnson N, Kyriacou A, Hayat S, Hobson NA, Mann I, Balasubramaniam R, Whinnett Z, Earley M, Petkar S, Veasey R, Kirubakaran S, Coyle C, Kim MY, Lim PB, O'Neill J, Davies DW, Peters NS, Babalis D, Linton N, Falaschetti E, Tanner M, Shah J, Poulter N. Ablation versus anti-arrhythmic therapy for reducing all hospital episodes from recurrent atrial fibrillation: a prospective, randomized, multi-centre, open label trial. Europace. 2023 Mar 30;25(3):863-872. doi: 10.1093/europace/euac253. PMID: 36576323; PMCID: PMC10062288.
26. Ding J, Cheng A, Li P, Yan Y, Shi Y, Xue Z, Sun S, Xu J. Cryoballoon catheter ablation or drug therapy to delay progression of atrial fibrillation: A single-center randomized trial. Front Cardiovasc Med. 2022 Oct 19;9:1003305. doi: 10.3389/fcvm.2022.1003305. PMID: 36337878; PMCID: PMC9627306.
27. Kirstein B, Tomala J, Mayer J, Ulbrich S, Wagner M, Pu L, Piorkowski J, Hankel A, Huo Y, Gaspar T, Richter U, Hindricks G, Piorkowski C. Effect of concomitant Renal DeNervation and cardiac ablation on Atrial Fibrillation recurrence - RDN+AF study. J Cardiovasc Electrophysiol. 2023 Jan;34(1):44-53. doi: 10.1111/jce.15714. Epub 2022 Dec 1. PMID: 36259713.
28. Baimbetov AK, Bizhanov KA, Jukenova AM, Aubakirova AT, Ualiyeva AY, Sagatov IY. Comparative Effectiveness and Safety of Cryoablation Versus Radiofrequency Ablation Treatments for Persistent Atrial Fibrillation. Am J Cardiol. 2022 Dec 1;184:22-30. doi: 10.1016/j.amjcard.2022.08.031. Epub 2022 Sep 30. PMID: 36184349.
29. Spitzer SG, Miller JM, Sommer P, Szili-Torok T, Reddy VY, Nölker G, Williams C, Sarver A, Wilber DJ. Randomized evaluation of redo ablation procedures of atrial fibrillation with focal impulse and rotor modulation-guided procedures: the REDO-FIRM study. Europace. 2023 Feb 8;25(1):74-82. doi: 10.1093/europace/euac122. PMID: 36056882; PMCID: PMC10103554.
30. Yang G, Zheng L, Jiang C, Fan J, Liu X, Zhan X, Li J, Wang L, Yang H, Zhu W, Du H, Ma G, Ma W, Kojodjojo P, Chen M; STABLE-SR-II Investigators. Circumferential Pulmonary Vein Isolation Plus Low-Voltage Area Modification in Persistent Atrial Fibrillation: The STABLE-SR-II Trial. JACC Clin Electrophysiol. 2022 Jul;8(7):882-891. doi: 10.1016/j.jacep.2022.03.012. Epub 2022 Apr 27. PMID: 35863814.
31. Marrouche NF, Wazni O, McGann C, Greene T, Dean JM, Dagher L, Kholmovski E, Mansour M, Marchlinski F, Wilber D, Hindricks G, Mahnkopf C, Wells D, Jais P, Sanders P, Brachmann J, Bax JJ, Morrison-de Boer L, Deneke T, Calkins H, Sohns C, Akoum N; DECAAF II Investigators. Effect of MRI-Guided Fibrosis Ablation vs Conventional Catheter Ablation on Atrial Arrhythmia Recurrence in Patients With Persistent Atrial Fibrillation: The DECAAF II Randomized Clinical Trial. JAMA. 2022 Jun 21;327(23):2296-2305. doi: 10.1001/jama.2022.8831. PMID: 35727277; PMCID: PMC9214588.
32. Kim D, Yu HT, Kim TH, Uhm JS, Joung B, Lee MH, Pak HN. Electrical Posterior Box Isolation in Repeat Ablation for Atrial Fibrillation: A Prospective Randomized Clinical Study. JACC Clin Electrophysiol. 2022 May;8(5):582-592. doi: 10.1016/j.jacep.2022.01.003. Epub 2022 Feb 23. PMID: 35589170.
33. Parkash R, Wells GA, Rouleau J, Talajic M, Essebag V, Skanes A, Wilton SB, Verma A, Healey JS, Sterns L, Bennett M, Roux JF, Rivard L, Leong-Sit P, Jensen-Urstad M, Jolly U, Philippon F, Sapp JL, Tang ASL. Randomized Ablation-Based Rhythm-Control Versus Rate-Control Trial in Patients With Heart Failure and Atrial Fibrillation: Results from the RAFT-AF trial. Circulation. 2022 Jun 7;145(23):1693-1704. doi: 10.1161/CIRCULATIONAHA.121.057095. Epub 2022 Mar 22. PMID: 35313733.
34. Ahn J, Shin DG, Han SJ, Lim HE. Does isolation of the left atrial posterior wall using cryoballoon ablation improve clinical outcomes in patients with persistent atrial fibrillation? A prospective randomized controlled trial. Europace. 2022 Jul 21;24(7):1093-1101. doi: 10.1093/europace/euac005. PMID: 35138376.
35. Theis C, Kaiser B, Kaesemann P, Hui F, Pirozzolo G, Bekeredjian R, Huber C. Pulmonary vein isolation using cryoballoon ablation versus RF ablation using ablation index following the CLOSE protocol: A prospective randomized trial. J Cardiovasc Electrophysiol. 2022 May;33(5):866-873. doi: 10.1111/jce.15383. Epub 2022 Mar 14. PMID: 35066944.
36. Wazni O, Dandamudi G, Sood N, Hoyt R, Tyler J, Durrani S, Niebauer M, Makati K, Halperin B, Gauri A, Morales G, Shao M, Pouliot E, Kaplon RE, Nissen SE; STOP AF First Trial Investigators. Quality of life after the initial treatment of atrial fibrillation with cryoablation versus drug therapy. Heart Rhythm. 2022 Feb;19(2):197-205. doi: 10.1016/j.hrthm.2021.10.009. Epub 2021 Oct 16. PMID: 34666139.
37. Bahnson TD, Giczewska A, Mark DB, Russo AM, Monahan KH, Al-Khalidi HR, Silverstein AP, Poole JE, Lee KL, Packer DL; CABANA Investigators. Association Between Age and Outcomes of Catheter Ablation Versus Medical Therapy for Atrial Fibrillation: Results From the CABANA Trial. Circulation. 2022 Mar 15;145(11):796-804. doi: 10.1161/CIRCULATIONAHA.121.055297. Epub 2021 Dec 22. PMID: 34933570; PMCID: PMC9003625.
38. Masuda M, Asai M, Iida O, Okamoto S, Ishihara T, Nanto K, Kanda T, Tsujimura T, Matsuda Y, Hata Y, Uematsu H, Mano T. Low-Voltage-Area Ablation in Paroxysmal Atrial Fibrillation　- Extended Follow-up Results of the VOLCANO Trial. Circ J. 2022 Jan 25;86(2):245-252. doi: 10.1253/circj.CJ-21-0476. Epub 2021 Jul 28. PMID: 34321377.
39. Willems S, Borof K, Brandes A, Breithardt G, Camm AJ, Crijns HJGM, Eckardt L, Gessler N, Goette A, Haegeli LM, Heidbuchel H, Kautzner J, Ng GA, Schnabel RB, Suling A, Szumowski L, Themistoclakis S, Vardas P, van Gelder IC, Wegscheider K, Kirchhof P. Systematic, early rhythm control strategy for atrial fibrillation in patients with or without symptoms: the EAST-AFNET 4 trial. Eur Heart J. 2022 Mar 21;43(12):1219-1230. doi: 10.1093/eurheartj/ehab593. PMID: 34447995; PMCID: PMC8934687.
40. Brignole M, Pentimalli F, Palmisano P, Landolina M, Quartieri F, Occhetta E, Calò L, Mascia G, Mont L, Vernooy K, van Dijk V, Allaart C, Fauchier L, Gasparini M, Parati G, Soranna D, Rienstra M, Van Gelder IC; APAF-CRT Trial Investigators. AV junction ablation and cardiac resynchronization for patients with permanent atrial fibrillation and narrow QRS: the APAF-CRT mortality trial. Eur Heart J. 2021 Dec 7;42(46):4731-4739. doi: 10.1093/eurheartj/ehab569. Erratum in: Eur Heart J. 2021 Oct 16;: Erratum in: Eur Heart J. 2021 Dec 08;: PMID: 34453840.
41. Pak HN, Park JW, Yang SY, Kim TH, Uhm JS, Joung B, Lee MH, Yu HT. Cryoballoon Versus High-Power, Short-Duration Radiofrequency Ablation for Pulmonary Vein Isolation in Patients With Paroxysmal Atrial Fibrillation: A Single-Center, Prospective, Randomized Study. Circ Arrhythm Electrophysiol. 2021 Sep;14(9):e010040. doi: 10.1161/CIRCEP.121.010040. Epub 2021 Sep 1. PMID: 34465132.
42. Samuel M, Khairy P, Champagne J, Deyell MW, Macle L, Leong-Sit P, Novak P, Badra-Verdu M, Sapp J, Tardif JC, Andrade JG. Association of Atrial Fibrillation Burden With Health-Related Quality of Life After Atrial Fibrillation Ablation: Substudy of the Cryoballoon vs Contact-Force Atrial Fibrillation Ablation (CIRCA-DOSE) Randomized Clinical Trial. JAMA Cardiol. 2021 Nov 1;6(11):1324-1328. doi: 10.1001/jamacardio.2021.3063. PMID: 34406350; PMCID: PMC8374730.
43. Rillig A, Magnussen C, Ozga AK, Suling A, Brandes A, Breithardt G, Camm AJ, Crijns HJGM, Eckardt L, Elvan A, Goette A, Gulizia M, Haegeli L, Heidbuchel H, Kuck KH, Ng A, Szumowski L, van Gelder I, Wegscheider K, Kirchhof P. Early Rhythm Control Therapy in Patients With Atrial Fibrillation and Heart Failure. Circulation. 2021 Sep 14;144(11):845-858. doi: 10.1161/CIRCULATIONAHA.121.056323. Epub 2021 Jul 30. PMID: 34328366; PMCID: PMC8456351.
44. Sørensen SK, Johannessen A, Worck R, Hansen ML, Hansen J. Radiofrequency Versus Cryoballoon Catheter Ablation for Paroxysmal Atrial Fibrillation: Durability of Pulmonary Vein Isolation and Effect on Atrial Fibrillation Burden: The RACE-AF Randomized Controlled Trial. Circ Arrhythm Electrophysiol. 2021 May;14(5):e009573. doi: 10.1161/CIRCEP.120.009573. Epub 2021 Apr 9. PMID: 33835823; PMCID: PMC8136462.
45. Kuniss M, Pavlovic N, Velagic V, Hermida JS, Healey S, Arena G, Badenco N, Meyer C, Chen J, Iacopino S, Anselme F, Packer DL, Pitschner HF, Asmundis C, Willems S, Di Piazza F, Becker D, Chierchia GB; Cryo-FIRST Investigators. Cryoballoon ablation vs. antiarrhythmic drugs: first-line therapy for patients with paroxysmal atrial fibrillation. Europace. 2021 Jul 18;23(7):1033-1041. doi: 10.1093/europace/euab029. PMID: 33728429; PMCID: PMC8286851.
46. Aryana A, Allen SL, Pujara DK, Bowers MR, O'Neill PG, Yamauchi Y, Shigeta T, Vierra EC, Okishige K, Natale A. Concomitant Pulmonary Vein and Posterior Wall Isolation Using Cryoballoon With Adjunct Radiofrequency in Persistent Atrial Fibrillation. JACC Clin Electrophysiol. 2021 Feb;7(2):187-196. doi: 10.1016/j.jacep.2020.08.016. Epub 2020 Oct 28. PMID: 33602399.
47. Packer DL, Piccini JP, Monahan KH, Al-Khalidi HR, Silverstein AP, Noseworthy PA, Poole JE, Bahnson TD, Lee KL, Mark DB; CABANA Investigators. Ablation Versus Drug Therapy for Atrial Fibrillation in Heart Failure: Results From the CABANA Trial. Circulation. 2021 Apr 6;143(14):1377-1390. doi: 10.1161/CIRCULATIONAHA.120.050991. Epub 2021 Feb 8. PMID: 33554614; PMCID: PMC8030730.
48. Kim MY, Coyle C, Tomlinson DR, Sikkel MB, Sohaib A, Luther V, Leong KM, Malcolme-Lawes L, Low B, Sandler B, Lim E, Todd M, Fudge M, Wright IJ, Koa-Wing M, Ng FS, Qureshi NA, Whinnett ZI, Peters NS, Newcomb D, Wood C, Dhillon G, Hunter RJ, Lim PB, Linton NWF, Kanagaratnam P. Ectopy-triggering ganglionated plexuses ablation to prevent atrial fibrillation: GANGLIA-AF study. Heart Rhythm. 2022 Apr;19(4):516-524. doi: 10.1016/j.hrthm.2021.12.010. Epub 2021 Dec 13. PMID: 34915187; PMCID: PMC8976158.
49. Sandler B, Kim MY, Sikkel MB, Malcolme-Lawes L, Koa-Wing M, Whinnett ZI, Coyle C, Linton NWF, Lim PB, Kanagaratnam P; other members of the Imperial College London, Cardiovascular Study Group/Consortium. Targeting the ectopy-triggering ganglionated plexuses without pulmonary vein isolation prevents atrial fibrillation. J Cardiovasc Electrophysiol. 2021 Feb;32(2):235-244. doi: 10.1111/jce.14870. Epub 2021 Jan 19. PMID: 33421265; PMCID: PMC8611799.
50. Chun JKR, Bordignon S, Last J, Mayer L, Tohoku S, Zanchi S, Bianchini L, Bologna F, Nagase T, Urbanek L, Chen S, Schmidt B. Cryoballoon Versus Laserballoon: Insights From the First Prospective Randomized Balloon Trial in Catheter Ablation of Atrial Fibrillation. Circ Arrhythm Electrophysiol. 2021 Feb;14(2):e009294. doi: 10.1161/CIRCEP.120.009294. Epub 2021 Jan 8. PMID: 33417476.
51. Wu G, Huang H, Cai L, Yang Y, Liu X, Yu B, Tang Y, Jiang H, Huang C; CAPA Study Investigators. Long-term observation of catheter ablation vs. pharmacotherapy in the management of persistent and long-standing persistent atrial fibrillation (CAPA study). Europace. 2021 May 21;23(5):731-739. doi: 10.1093/europace/euaa356. PMID: 33367669.
52. Tilz RR, Lenz C, Sommer P, Roza MS, Sarver AE, Williams CG, Heeger C, Hindricks G, Vogler J, Eitel C. Focal Impulse and Rotor Modulation Ablation vs. Pulmonary Vein isolation for the treatment of paroxysmal Atrial Fibrillation: results from the FIRMAP AF study. Europace. 2021 May 21;23(5):722-730. doi: 10.1093/europace/euaa378. PMID: 33351076; PMCID: PMC8139814.
53. Sugumar H, Prabhu S, Costello B, Chieng D, Azzopardi S, Voskoboinik A, Parameswaran R, Wong GR, Anderson R, Al-Kaisey AM, Ling LH, Kotschet E, Taylor AJ, Kalman JM, Kistler PM. Catheter Ablation Versus Medication in Atrial Fibrillation and Systolic Dysfunction: Late Outcomes of CAMERA-MRI Study. JACC Clin Electrophysiol. 2020 Dec 14;6(13):1721-1731. doi: 10.1016/j.jacep.2020.08.019. Epub 2020 Oct 28. PMID: 33334453.
54. Kuck KH, Lebedev DS, Mikhaylov EN, Romanov A, Gellér L, Kalējs O, Neumann T, Davtyan K, On YK, Popov S, Bongiorni MG, Schlüter M, Willems S, Ouyang F. Catheter ablation or medical therapy to delay progression of atrial fibrillation: the randomized controlled atrial fibrillation progression trial (ATTEST). Europace. 2021 Mar 8;23(3):362-369. doi: 10.1093/europace/euaa298. PMID: 33330909; PMCID: PMC7947582.
55. Andrade JG, Wells GA, Deyell MW, Bennett M, Essebag V, Champagne J, Roux JF, Yung D, Skanes A, Khaykin Y, Morillo C, Jolly U, Novak P, Lockwood E, Amit G, Angaran P, Sapp J, Wardell S, Lauck S, Macle L, Verma A; EARLY-AF Investigators. Cryoablation or Drug Therapy for Initial Treatment of Atrial Fibrillation. N Engl J Med. 2021 Jan 28;384(4):305-315. doi: 10.1056/NEJMoa2029980. Epub 2020 Nov 16. PMID: 33197159.
56. Wazni OM, Dandamudi G, Sood N, Hoyt R, Tyler J, Durrani S, Niebauer M, Makati K, Halperin B, Gauri A, Morales G, Shao M, Cerkvenik J, Kaplon RE, Nissen SE; STOP AF First Trial Investigators. Cryoballoon Ablation as Initial Therapy for Atrial Fibrillation. N Engl J Med. 2021 Jan 28;384(4):316-324. doi: 10.1056/NEJMoa2029554. Epub 2020 Nov 16. PMID: 33197158.
57. Wielandts JY, Kyriakopoulou M, Almorad A, Hilfiker G, Strisciuglio T, Phlips T, El Haddad M, Lycke M, Unger P, Le Polain de Waroux JB, Vandekerckhove Y, Tavernier R, Duytschaever M, Knecht S. Prospective Randomized Evaluation of High Power During CLOSE-Guided Pulmonary Vein Isolation: The POWER-AF Study. Circ Arrhythm Electrophysiol. 2021 Jan;14(1):e009112. doi: 10.1161/CIRCEP.120.009112. Epub 2020 Dec 10. PMID: 33300809.
58. Pavlovic N, Chierchia GB, Velagic V, Hermida JS, Healey S, Arena G, Badenco N, Meyer C, Chen J, Iacopino S, Anselme F, Dekker L, Scazzuso F, Packer DL, de Asmundis C, Pitschner HF, Piazza FD, Kaplon RE, Kuniss M; Cryo-FIRST Investigators. Initial rhythm control with cryoballoon ablation vs drug therapy: Impact on quality of life and symptoms. Am Heart J. 2021 Dec;242:103-114. doi: 10.1016/j.ahj.2021.08.007. Epub 2021 Sep 8. PMID: 34508694.
59. Inoue K, Hikoso S, Masuda M, Furukawa Y, Hirata A, Egami Y, Watanabe T, Minamiguchi H, Miyoshi M, Tanaka N, Oka T, Okada M, Kanda T, Matsuda Y, Kawasaki M, Hayashi K, Kitamura T, Dohi T, Sunaga A, Mizuno H, Nakatani D, Sakata Y; OCVC Arrhythmia Investigators. Pulmonary vein isolation alone vs. more extensive ablation with defragmentation and linear ablation of persistent atrial fibrillation: the EARNEST-PVI trial. Europace. 2021 Apr 6;23(4):565-574. doi: 10.1093/europace/euaa293. PMID: 33200213.
60. Gallagher MM, Yi G, Gonna H, Leung LWM, Harding I, Evranos B, Bastiaenen R, Sharma R, Wright S, Norman M, Zuberi Z, Camm AJ. Multi-catheter cryotherapy compared with radiofrequency ablation in long-standing persistent atrial fibrillation: a randomized clinical trial. Europace. 2021 Mar 8;23(3):370-379. doi: 10.1093/europace/euaa289. PMID: 33188692.
61. Kim SH, Oh YS, Choi Y, Hwang Y, Kim JY, Kim TS, Kim JH, Jang SW, Lee MY, Joung B, Choi KJ. Long-Term Efficacy of Prophylactic Cavotricuspid Isthmus Ablation during Atrial Fibrillation Ablation in Patients Without Typical Atrial Flutter: a Prospective, Multicentre, Randomized Trial. Korean Circ J. 2021 Jan;51(1):58-64. doi: 10.4070/kcj.2020.0174. Epub 2020 Sep 14. PMID: 33150753; PMCID: PMC7779821.
62. Bisbal F, Benito E, Teis A, Alarcón F, Sarrias A, Caixal G, Villuendas R, Garre P, Soto N, Cozzari J, Guasch E, Juncà G, Prat-Gonzalez S, Perea RJ, Bazán V, Tolosana JM, Arbelo E, Bayés-Genís A, Mont L. Magnetic Resonance Imaging-Guided Fibrosis Ablation for the Treatment of Atrial Fibrillation: The ALICIA Trial. Circ Arrhythm Electrophysiol. 2020 Nov;13(11):e008707. doi: 10.1161/CIRCEP.120.008707. Epub 2020 Oct 8. PMID: 33031713.
63. Valderrábano M, Peterson LE, Swarup V, Schurmann PA, Makkar A, Doshi RN, DeLurgio D, Athill CA, Ellenbogen KA, Natale A, Koneru J, Dave AS, Giorgberidze I, Afshar H, Guthrie ML, Bunge R, Morillo CA, Kleiman NS. Effect of Catheter Ablation With Vein of Marshall Ethanol Infusion vs Catheter Ablation Alone on Persistent Atrial Fibrillation: The VENUS Randomized Clinical Trial. JAMA. 2020 Oct 27;324(16):1620-1628. doi: 10.1001/jama.2020.16195. PMID: 33107945; PMCID: PMC7592031.
64. Yao Y, Hu F, Du Z, He J, Shi H, Zhang J, Cai H, Jia Y, Tang M, Niu G, Chen G, Ding L, Zheng L, Liang E, Wu L. The value of extensive catheter linear ablation on persistent atrial fibrillation (the CLEAR-AF Study). Int J Cardiol. 2020 Oct 1;316:125-129. doi: 10.1016/j.ijcard.2020.05.032. Epub 2020 May 24. PMID: 32461117.
65. Sohns C, Zintl K, Zhao Y, Dagher L, Andresen D, Siebels J, Wegscheider K, Sehner S, Boersma L, Merkely B, Pokushalov E, Sanders P, Schunkert H, Bänsch D, Mahnkopf C, Brachmann J, Marrouche NF. Impact of Left Ventricular Function and Heart Failure Symptoms on Outcomes Post Ablation of Atrial Fibrillation in Heart Failure: CASTLE-AF Trial. Circ Arrhythm Electrophysiol. 2020 Oct;13(10):e008461. doi: 10.1161/CIRCEP.120.008461. Epub 2020 Sep 9. PMID: 32903044.
66. Shin DG, Ahn J, Han SJ, Lim HE. Efficacy of high-power and short-duration ablation in patients with atrial fibrillation: a prospective randomized controlled trial. Europace. 2020 Oct 1;22(10):1495-1501. doi: 10.1093/europace/euaa144. PMID: 32810203.
67. Pak HN, Park J, Park JW, Yang SY, Yu HT, Kim TH, Uhm JS, Choi JI, Joung B, Lee MH, Kim YH, Shim J. Electrical Posterior Box Isolation in Persistent Atrial Fibrillation Changed to Paroxysmal Atrial Fibrillation: A Multicenter, Prospective, Randomized Study. Circ Arrhythm Electrophysiol. 2020 Sep;13(9):e008531. doi: 10.1161/CIRCEP.120.008531. Epub 2020 Jul 28. PMID: 32755396.
68. Masuda M, Asai M, Iida O, Okamoto S, Ishihara T, Nanto K, Kanda T, Tsujimura T, Matsuda Y, Okuno S, Hata Y, Mano T. Additional Low-Voltage-Area Ablation in Patients With Paroxysmal Atrial Fibrillation: Results of the Randomized Controlled VOLCANO Trial. J Am Heart Assoc. 2020 Jul 7;9(13):e015927. doi: 10.1161/JAHA.120.015927. Epub 2020 Jun 24. PMID: 32578466; PMCID: PMC7670527.
69. Zhang X, Kuang X, Gao X, Xiang H, Wei F, Liu T, Wu H, Wang G, Zuo Z, Wang L, Ding L, Zhang J, Shehata M, Wang X, Yang B, Fan J. RESCUE-AF in Patients Undergoing Atrial Fibrillation Ablation: The RESCUE-AF Trial. Circ Arrhythm Electrophysiol. 2019 May;12(5):e007044. doi: 10.1161/CIRCEP.118.007044. PMID: 32125792.
70. Chauhan VS, Verma A, Nayyar S, Timmerman N, Tomlinson G, Porta-Sanchez A, Gizurarson S, Haldar S, Suszko A, Ragot D, Ha ACT. Focal source and trigger mapping in atrial fibrillation: Randomized controlled trial evaluating a novel adjunctive ablation strategy. Heart Rhythm. 2020 May;17(5 Pt A):683-691. doi: 10.1016/j.hrthm.2019.12.011. Epub 2020 Jan 25. PMID: 31991116.
71. Steinberg JS, Shabanov V, Ponomarev D, Losik D, Ivanickiy E, Kropotkin E, Polyakov K, Ptaszynski P, Keweloh B, Yao CJ, Pokushalov EA, Romanov AB. Effect of Renal Denervation and Catheter Ablation vs Catheter Ablation Alone on Atrial Fibrillation Recurrence Among Patients With Paroxysmal Atrial Fibrillation and Hypertension: The ERADICATE-AF Randomized Clinical Trial. JAMA. 2020 Jan 21;323(3):248-255. doi: 10.1001/jama.2019.21187. Erratum in: JAMA. 2020 Mar 3;323(9):896. PMID: 31961420; PMCID: PMC6990678.
72. Xu Q, Ju W, Xiao F, Yang B, Chen H, Yang G, Zhang F, Gu K, Li M, Wang D, Chen M. Circumferential pulmonary vein antrum ablation for the treatment of paroxysmal atrial fibrillation: A randomized controlled trial. Pacing Clin Electrophysiol. 2020 Mar;43(3):280-288. doi: 10.1111/pace.13863. Epub 2020 Feb 5. PMID: 31849079.
73. Kuck KH, Merkely B, Zahn R, Arentz T, Seidl K, Schlüter M, Tilz RR, Piorkowski C, Gellér L, Kleemann T, Hindricks G. Catheter Ablation Versus Best Medical Therapy in Patients With Persistent Atrial Fibrillation and Congestive Heart Failure: The Randomized AMICA Trial. Circ Arrhythm Electrophysiol. 2019 Dec;12(12):e007731. doi: 10.1161/CIRCEP.119.007731. Epub 2019 Nov 25. PMID: 31760819.
74. Lee JM, Shim J, Park J, Yu HT, Kim TH, Park JK, Uhm JS, Kim JB, Joung B, Lee MH, Kim YH, Pak HN; POBI-AF Investigators. The Electrical Isolation of the Left Atrial Posterior Wall in Catheter Ablation of Persistent Atrial Fibrillation. JACC Clin Electrophysiol. 2019 Nov;5(11):1253-1261. doi: 10.1016/j.jacep.2019.08.021. Epub 2019 Oct 30. Erratum in: JACC Clin Electrophysiol. 2023 Jan;9(1):145. PMID: 31753429.
75. Andrade JG, Champagne J, Dubuc M, Deyell MW, Verma A, Macle L, Leong-Sit P, Novak P, Badra-Verdu M, Sapp J, Mangat I, Khoo C, Steinberg C, Bennett MT, Tang ASL, Khairy P; CIRCA-DOSE Study Investigators. Cryoballoon or Radiofrequency Ablation for Atrial Fibrillation Assessed by Continuous Monitoring: A Randomized Clinical Trial. Circulation. 2019 Nov 26;140(22):1779-1788. doi: 10.1161/CIRCULATIONAHA.119.042622. Epub 2019 Oct 21. PMID: 31630538.
76. Nogami A, Harada T, Sekiguchi Y, Otani R, Yoshida Y, Yoshida K, Nakano Y, Nuruki N, Nakahara S, Goya M, Origasa H, Kihara Y, Hirao K, Aonuma K; ABRIDGE-J (ABlation peRIoperative DabiGatran in use Envisioning in Japan) Investigators. Safety and Efficacy of Minimally Interrupted Dabigatran vs Uninterrupted Warfarin Therapy in Adults Undergoing Atrial Fibrillation Catheter Ablation: A Randomized Clinical Trial. JAMA Netw Open. 2019 Apr 5;2(4):e191994. doi: 10.1001/jamanetworkopen.2019.1994. PMID: 31002317; PMCID: PMC6481436.
77. Hohnloser SH, Camm J, Cappato R, Diener HC, Heidbüchel H, Mont L, Morillo CA, Abozguia K, Grimaldi M, Rauer H, Reimitz PE, Smolnik R, Mönninghoff C, Kautzner J. Uninterrupted edoxaban vs. vitamin K antagonists for ablation of atrial fibrillation: the ELIMINATE-AF trial. Eur Heart J. 2019 Sep 21;40(36):3013-3021. doi: 10.1093/eurheartj/ehz190. Erratum in: Eur Heart J. 2019 Sep 21;40(36):3043. PMID: 30976787; PMCID: PMC6754569.
78. Packer DL, Mark DB, Robb RA, Monahan KH, Bahnson TD, Poole JE, Noseworthy PA, Rosenberg YD, Jeffries N, Mitchell LB, Flaker GC, Pokushalov E, Romanov A, Bunch TJ, Noelker G, Ardashev A, Revishvili A, Wilber DJ, Cappato R, Kuck KH, Hindricks G, Davies DW, Kowey PR, Naccarelli GV, Reiffel JA, Piccini JP, Silverstein AP, Al-Khalidi HR, Lee KL; CABANA Investigators. Effect of Catheter Ablation vs Antiarrhythmic Drug Therapy on Mortality, Stroke, Bleeding, and Cardiac Arrest Among Patients With Atrial Fibrillation: The CABANA Randomized Clinical Trial. JAMA. 2019 Apr 2;321(13):1261-1274. doi: 10.1001/jama.2019.0693. PMID: 30874766; PMCID: PMC6450284.
79. Nagao T, Suzuki H, Matsunaga S, Nishikawa Y, Harada K, Mamiya K, Shinoda N, Harada K, Kato M, Marui N, Amano T, Inden Y, Murohara T. Impact of periprocedural anticoagulation therapy on the incidence of silent stroke after atrial fibrillation ablation in patients receiving direct oral anticoagulants: uninterrupted vs. interrupted by one dose strategy. Europace. 2019 Apr 1;21(4):590-597. doi: 10.1093/europace/euy224. PMID: 30376051.
80. Blomström-Lundqvist C, Gizurarson S, Schwieler J, Jensen SM, Bergfeldt L, Kennebäck G, Rubulis A, Malmborg H, Raatikainen P, Lönnerholm S, Höglund N, Mörtsell D. Effect of Catheter Ablation vs Antiarrhythmic Medication on Quality of Life in Patients With Atrial Fibrillation: The CAPTAF Randomized Clinical Trial. JAMA. 2019 Mar 19;321(11):1059-1068. doi: 10.1001/jama.2019.0335. PMID: 30874754; PMCID: PMC6439911.
81. Mark DB, Anstrom KJ, Sheng S, Piccini JP, Baloch KN, Monahan KH, Daniels MR, Bahnson TD, Poole JE, Rosenberg Y, Lee KL, Packer DL; CABANA Investigators. Effect of Catheter Ablation vs Medical Therapy on Quality of Life Among Patients With Atrial Fibrillation: The CABANA Randomized Clinical Trial. JAMA. 2019 Apr 2;321(13):1275-1285. doi: 10.1001/jama.2019.0692. Erratum in: JAMA. 2019 Jun 18;321(23):2370. PMID: 30874716; PMCID: PMC6450275.
82. Lee KN, Choi JI, Kim YG, Oh SK, Kim DH, Lee DI, Roh SY, Ahn JH, Shim J, Park SW, Kim YH. Comparison between linear and focal ablation of complex fractionated atrial electrograms in patients with non-paroxysmal atrial fibrillation: a prospective randomized trial. Europace. 2019 Apr 1;21(4):598-606. doi: 10.1093/europace/euy313. PMID: 30649276.
83. Kimura T, Kashimura S, Nishiyama T, Katsumata Y, Inagawa K, Ikegami Y, Nishiyama N, Fukumoto K, Tanimoto Y, Aizawa Y, Tanimoto K, Fukuda K, Takatsuki S. Asymptomatic Cerebral Infarction During Catheter Ablation for Atrial Fibrillation: Comparing Uninterrupted Rivaroxaban and Warfarin (ASCERTAIN). JACC Clin Electrophysiol. 2018 Dec;4(12):1598-1609. doi: 10.1016/j.jacep.2018.08.003. Epub 2018 Sep 26. PMID: 30573125.
84. Nakamura K, Naito S, Sasaki T, Take Y, Minami K, Kitagawa Y, Motoda H, Inoue M, Otsuka Y, Niijima K, Yamashita E, Sugai Y, Kumagai K, Koyama K, Funabashi N, Oshima S. Uninterrupted vs. interrupted periprocedural direct oral anticoagulants for catheter ablation of atrial fibrillation: a prospective randomized single-centre study on post-ablation thrombo-embolic and haemorrhagic events. Europace. 2019 Feb 1;21(2):259-267. doi: 10.1093/europace/euy148. PMID: 29982562.
85. Lee KN, Roh SY, Baek YS, Park HS, Ahn J, Kim DH, Lee DI, Shim J, Choi JI, Park SW, Kim YH. Long-Term Clinical Comparison of Procedural End Points After Pulmonary Vein Isolation in Paroxysmal Atrial Fibrillation: Elimination of Nonpulmonary Vein Triggers Versus Noninducibility. Circ Arrhythm Electrophysiol. 2018 Feb;11(2):e005019. doi: 10.1161/CIRCEP.117.005019. PMID: 29431632.
86. Marrouche NF, Brachmann J, Andresen D, Siebels J, Boersma L, Jordaens L, Merkely B, Pokushalov E, Sanders P, Proff J, Schunkert H, Christ H, Vogt J, Bänsch D; CASTLE-AF Investigators. Catheter Ablation for Atrial Fibrillation with Heart Failure. N Engl J Med. 2018 Feb 1;378(5):417-427. doi: 10.1056/NEJMoa1707855. PMID: 29385358.
87. Rillig A, Schmidt B, Di Biase L, Lin T, Scholz L, Heeger CH, Metzner A, Steven D, Wohlmuth P, Willems S, Trivedi C, Galllinghouse JG, Natale A, Ouyang F, Kuck KH, Tilz RR. Manual Versus Robotic Catheter Ablation for the Treatment of Atrial Fibrillation: The Man and Machine Trial. JACC Clin Electrophysiol. 2017 Aug;3(8):875-883. doi: 10.1016/j.jacep.2017.01.024. Epub 2017 Jun 28. PMID: 29759785.
88. Duytschaever M, Demolder A, Phlips T, Sarkozy A, El Haddad M, Taghji P, Knecht S, Tavernier R, Vandekerckhove Y, De Potter T. PulmOnary vein isolation With vs. without continued antiarrhythmic Drug trEatment in subjects with Recurrent Atrial Fibrillation (POWDER AF): results from a multicentre randomized trial. Eur Heart J. 2018 Apr 21;39(16):1429-1437. doi: 10.1093/eurheartj/ehx666. PMID: 29211857.
89. Kircher S, Arya A, Altmann D, Rolf S, Bollmann A, Sommer P, Dagres N, Richter S, Breithardt OA, Dinov B, Husser D, Eitel C, Gaspar T, Piorkowski C, Hindricks G. Individually tailored vs. standardized substrate modification during radiofrequency catheter ablation for atrial fibrillation: a randomized study. Europace. 2018 Nov 1;20(11):1766-1775. doi: 10.1093/europace/eux310. PMID: 29177475.
90. Yang B, Jiang C, Lin Y, Yang G, Chu H, Cai H, Lu F, Zhan X, Xu J, Wang X, Ching CK, Singh B, Kim YH, Chen M; STABLE-SR Investigators*. STABLE-SR (Electrophysiological Substrate Ablation in the Left Atrium During Sinus Rhythm) for the Treatment of Nonparoxysmal Atrial Fibrillation: A Prospective, Multicenter Randomized Clinical Trial. Circ Arrhythm Electrophysiol. 2017 Nov;10(11):e005405. doi: 10.1161/CIRCEP.117.005405. PMID: 29141843.
91. Wang YL, Liu X, Zhang Y, Jiang WF, Zhou L, Qin M, Zhang DL, Zhang XD, Wu SH, Xu K. Optimal endpoint for catheter ablation of longstanding persistent atrial fibrillation: A randomized clinical trial. Pacing Clin Electrophysiol. 2018 Feb;41(2):172-178. doi: 10.1111/pace.13221. Epub 2018 Jan 16. PMID: 29023875.
92. Prabhu S, Taylor AJ, Costello BT, Kaye DM, McLellan AJA, Voskoboinik A, Sugumar H, Lockwood SM, Stokes MB, Pathik B, Nalliah CJ, Wong GR, Azzopardi SM, Gutman SJ, Lee G, Layland J, Mariani JA, Ling LH, Kalman JM, Kistler PM. Catheter Ablation Versus Medical Rate Control in Atrial Fibrillation and Systolic Dysfunction: The CAMERA-MRI Study. J Am Coll Cardiol. 2017 Oct 17;70(16):1949-1961. doi: 10.1016/j.jacc.2017.08.041. Epub 2017 Aug 27. PMID: 28855115.
93. Fink T, Schlüter M, Heeger CH, Lemes C, Maurer T, Reissmann B, Riedl J, Rottner L, Santoro F, Schmidt B, Wohlmuth P, Mathew S, Sohns C, Ouyang F, Metzner A, Kuck KH. Stand-Alone Pulmonary Vein Isolation Versus Pulmonary Vein Isolation With Additional Substrate Modification as Index Ablation Procedures in Patients With Persistent and Long-Standing Persistent Atrial Fibrillation: The Randomized Alster-Lost-AF Trial (Ablation at St. Georg Hospital for Long-Standing Persistent Atrial Fibrillation). Circ Arrhythm Electrophysiol. 2017 Jul;10(7):e005114. doi: 10.1161/CIRCEP.117.005114. PMID: 28687670.
94. Yu HT, Shim J, Park J, Kim IS, Kim TH, Uhm JS, Joung B, Lee MH, Kim YH, Pak HN. Pulmonary Vein Isolation Alone Versus Additional Linear Ablation in Patients With Persistent Atrial Fibrillation Converted to Paroxysmal Type With Antiarrhythmic Drug Therapy: A Multicenter, Prospective, Randomized Study. Circ Arrhythm Electrophysiol. 2017 Jun;10(6):e004915. doi: 10.1161/CIRCEP.116.004915. PMID: 28611206.
95. Calkins H, Willems S, Gerstenfeld EP, Verma A, Schilling R, Hohnloser SH, Okumura K, Serota H, Nordaby M, Guiver K, Biss B, Brouwer MA, Grimaldi M; RE-CIRCUIT Investigators. Uninterrupted Dabigatran versus Warfarin for Ablation in Atrial Fibrillation. N Engl J Med. 2017 Apr 27;376(17):1627-1636. doi: 10.1056/NEJMoa1701005. Epub 2017 Mar 19. PMID: 28317415.
96. Kim TH, Uhm JS, Kim JY, Joung B, Lee MH, Pak HN. Does Additional Electrogram-Guided Ablation After Linear Ablation Reduce Recurrence After Catheter Ablation for Longstanding Persistent Atrial Fibrillation? A Prospective Randomized Study. J Am Heart Assoc. 2017 Feb 7;6(2):e004811. doi: 10.1161/JAHA.116.004811. PMID: 28174170; PMCID: PMC5523774.
97. Gunawardene MA, Hoffmann BA, Schaeffer B, Chung DU, Moser J, Akbulak RO, Jularic M, Eickholt C, Nuehrich J, Meyer C, Willems S. Influence of energy source on early atrial fibrillation recurrences: a comparison of cryoballoon vs. radiofrequency current energy ablation with the endpoint of unexcitability in pulmonary vein isolation. Europace. 2018 Jan 1;20(1):43-49. doi: 10.1093/europace/euw307. PMID: 27742775.
98. Parkash R, Wells GA, Sapp JL, Healey JS, Tardif JC, Greiss I, Rivard L, Roux JF, Gula L, Nault I, Novak P, Birnie D, Ha A, Wilton SB, Mangat I, Gray C, Gardner M, Tang ASL. Effect of Aggressive Blood Pressure Control on the Recurrence of Atrial Fibrillation After Catheter Ablation: A Randomized, Open-Label Clinical Trial (SMAC-AF [Substrate Modification With Aggressive Blood Pressure Control]). Circulation. 2017 May 9;135(19):1788-1798. doi: 10.1161/CIRCULATIONAHA.116.026230. Epub 2017 Feb 22. PMID: 28228428.
99. Nielsen JC, Johannessen A, Raatikainen P, Hindricks G, Walfridsson H, Pehrson SM, Englund A, Hartikainen J, Mortensen LS, Hansen PS; MANTRA-PAF Investigators. Long-term efficacy of catheter ablation as first-line therapy for paroxysmal atrial fibrillation: 5-year outcome in a randomised clinical trial. Heart. 2017 Mar;103(5):368-376. doi: 10.1136/heartjnl-2016-309781. Epub 2016 Aug 26. PMID: 27566295.
100. Sohara H, Ohe T, Okumura K, Naito S, Hirao K, Shoda M, Kobayashi Y, Yamauchi Y, Yamaguchi Y, Kuwahara T, Hirayama H, YeongHwa C, Kusano K, Kaitani K, Banba K, Fujii S, Kumagai K, Yoshida H, Matsushita M, Satake S, Aonuma K. HotBalloon Ablation of the Pulmonary Veins for Paroxysmal AF: A Multicenter Randomized Trial in Japan. J Am Coll Cardiol. 2016 Dec 27;68(25):2747-2757. doi: 10.1016/j.jacc.2016.10.037. PMID: 28007137.
101. Driessen AHG, Berger WR, Krul SPJ, van den Berg NWE, Neefs J, Piersma FR, Chan Pin Yin DRPP, de Jong JSSG, van Boven WP, de Groot JR. Ganglion Plexus Ablation in Advanced Atrial Fibrillation: The AFACT Study. J Am Coll Cardiol. 2016 Sep 13;68(11):1155-1165. doi: 10.1016/j.jacc.2016.06.036. PMID: 27609676.
102. Kuck KH, Fürnkranz A, Chun KR, Metzner A, Ouyang F, Schlüter M, Elvan A, Lim HW, Kueffer FJ, Arentz T, Albenque JP, Tondo C, Kühne M, Sticherling C, Brugada J; FIRE AND ICE Investigators. Cryoballoon or radiofrequency ablation for symptomatic paroxysmal atrial fibrillation: reintervention, rehospitalization, and quality-of-life outcomes in the FIRE AND ICE trial. Eur Heart J. 2016 Oct 7;37(38):2858-2865. doi: 10.1093/eurheartj/ehw285. Epub 2016 Jul 5. PMID: 27381589; PMCID: PMC5070448.
103. Boersma LV, van der Voort P, Debruyne P, Dekker L, Simmers T, Rossenbacker T, Balt J, Wijffels M, Degreef Y. Multielectrode Pulmonary Vein Isolation Versus Single Tip Wide Area Catheter Ablation for Paroxysmal Atrial Fibrillation: A Multinational Multicenter Randomized Clinical Trial. Circ Arrhythm Electrophysiol. 2016 Apr;9(4):e003151. doi: 10.1161/CIRCEP.115.003151. PMID: 27071830.
104. Kuck KH, Brugada J, Fürnkranz A, Metzner A, Ouyang F, Chun KR, Elvan A, Arentz T, Bestehorn K, Pocock SJ, Albenque JP, Tondo C; FIRE AND ICE Investigators. Cryoballoon or Radiofrequency Ablation for Paroxysmal Atrial Fibrillation. N Engl J Med. 2016 Jun 9;374(23):2235-45. doi: 10.1056/NEJMoa1602014. Epub 2016 Apr 4. PMID: 27042964.
105. Di Biase L, Mohanty P, Mohanty S, Santangeli P, Trivedi C, Lakkireddy D, Reddy M, Jais P, Themistoclakis S, Dello Russo A, Casella M, Pelargonio G, Narducci ML, Schweikert R, Neuzil P, Sanchez J, Horton R, Beheiry S, Hongo R, Hao S, Rossillo A, Forleo G, Tondo C, Burkhardt JD, Haissaguerre M, Natale A. Ablation Versus Amiodarone for Treatment of Persistent Atrial Fibrillation in Patients With Congestive Heart Failure and an Implanted Device: Results From the AATAC Multicenter Randomized Trial. Circulation. 2016 Apr 26;133(17):1637-44. doi: 10.1161/CIRCULATIONAHA.115.019406. Epub 2016 Mar 30. PMID: 27029350.
106. Bassiouny M, Saliba W, Hussein A, Rickard J, Diab M, Aman W, Dresing T, Callahan T 4th, Bhargava M, Martin DO, Shao M, Baranowski B, Tarakji K, Tchou PJ, Hakim A, Kanj M, Lindsay B, Wazni O. Randomized Study of Persistent Atrial Fibrillation Ablation: Ablate in Sinus Rhythm Versus Ablate Complex-Fractionated Atrial Electrograms in Atrial Fibrillation. Circ Arrhythm Electrophysiol. 2016 Feb;9(2):e003596. doi: 10.1161/CIRCEP.115.003596. PMID: 26857909.
107. Kuwahara T, Abe M, Yamaki M, Fujieda H, Abe Y, Hashimoto K, Ishiba M, Sakai H, Hishikari K, Takigawa M, Okubo K, Takagi K, Tanaka Y, Nakajima J, Takahashi A. Apixaban versus Warfarin for the Prevention of Periprocedural Cerebral Thromboembolism in Atrial Fibrillation Ablation: Multicenter Prospective Randomized Study. J Cardiovasc Electrophysiol. 2016 May;27(5):549-54. doi: 10.1111/jce.12928. Epub 2016 Feb 12. PMID: 26766541.
108. Kaitani K, Inoue K, Kobori A, Nakazawa Y, Ozawa T, Kurotobi T, Morishima I, Miura F, Watanabe T, Masuda M, Naito M, Fujimoto H, Nishida T, Furukawa Y, Shirayama T, Tanaka M, Okajima K, Yao T, Egami Y, Satomi K, Noda T, Miyamoto K, Haruna T, Kawaji T, Yoshizawa T, Toyota T, Yahata M, Nakai K, Sugiyama H, Higashi Y, Ito M, Horie M, Kusano KF, Shimizu W, Kamakura S, Morimoto T, Kimura T, Shizuta S; EAST-AF Trial Investigators. Efficacy of Antiarrhythmic Drugs Short-Term Use After Catheter Ablation for Atrial Fibrillation (EAST-AF) trial. Eur Heart J. 2016 Feb 14;37(7):610-8. doi: 10.1093/eurheartj/ehv501. Epub 2015 Sep 28. PMID: 26417061.
109. Kuck KH, Hoffmann BA, Ernst S, Wegscheider K, Treszl A, Metzner A, Eckardt L, Lewalter T, Breithardt G, Willems S; Gap-AF–AFNET 1 Investigators*. Impact of Complete Versus Incomplete Circumferential Lines Around the Pulmonary Veins During Catheter Ablation of Paroxysmal Atrial Fibrillation: Results From the Gap-Atrial Fibrillation-German Atrial Fibrillation Competence Network 1 Trial. Circ Arrhythm Electrophysiol. 2016 Jan;9(1):e003337. doi: 10.1161/CIRCEP.115.003337. PMID: 26763226.
110. Wynn GJ, Panikker S, Morgan M, Hall M, Waktare J, Markides V, Hussain W, Salukhe T, Modi S, Jarman J, Jones DG, Snowdon R, Todd D, Wong T, Gupta D. Biatrial linear ablation in sustained nonpermanent AF: Results of the substrate modification with ablation and antiarrhythmic drugs in nonpermanent atrial fibrillation (SMAN-PAF) trial. Heart Rhythm. 2016 Feb;13(2):399-406. doi: 10.1016/j.hrthm.2015.10.006. Epub 2015 Oct 9. PMID: 26455343.
111. Hunter RJ, Baker V, Finlay MC, Duncan ER, Lovell MJ, Tayebjee MH, Ullah W, Siddiqui MS, McLEAN A, Richmond L, Kirkby C, Ginks MR, Dhinoja M, Sporton S, Earley MJ, Schilling RJ. Point-by-Point Radiofrequency Ablation Versus the Cryoballoon or a Novel Combined Approach: A Randomized Trial Comparing 3 Methods of Pulmonary Vein Isolation for Paroxysmal Atrial Fibrillation (The Cryo Versus RF Trial). J Cardiovasc Electrophysiol. 2015 Dec;26(12):1307-14. doi: 10.1111/jce.12846. Epub 2015 Nov 25. PMID: 26727045.
112. Kobori A, Shizuta S, Inoue K, Kaitani K, Morimoto T, Nakazawa Y, Ozawa T, Kurotobi T, Morishima I, Miura F, Watanabe T, Masuda M, Naito M, Fujimoto H, Nishida T, Furukawa Y, Shirayama T, Tanaka M, Okajima K, Yao T, Egami Y, Satomi K, Noda T, Miyamoto K, Haruna T, Kawaji T, Yoshizawa T, Toyota T, Yahata M, Nakai K, Sugiyama H, Higashi Y, Ito M, Horie M, Kusano KF, Shimizu W, Kamakura S, Kimura T; UNDER-ATP Trial Investigators. Adenosine triphosphate-guided pulmonary vein isolation for atrial fibrillation: the UNmasking Dormant Electrical Reconduction by Adenosine TriPhosphate (UNDER-ATP) trial. Eur Heart J. 2015 Dec 7;36(46):3276-87. doi: 10.1093/eurheartj/ehv457. Epub 2015 Aug 30. PMID: 26321237.
113. Vogler J, Willems S, Sultan A, Schreiber D, Lüker J, Servatius H, Schäffer B, Moser J, Hoffmann BA, Steven D. Pulmonary Vein Isolation Versus Defragmentation: The CHASE-AF Clinical Trial. J Am Coll Cardiol. 2015 Dec 22;66(24):2743-2752. doi: 10.1016/j.jacc.2015.09.088. PMID: 26700836.
114. Gula LJ, Leong-Sit P, Manlucu J, Hillock L, Yee R, Tang AS, Klein GJ, Skanes AC. Pulmonary Vein Isolation With Incomplete Antral Ablation Lines: Is More Ablation Necessary? Results of a Randomized Trial. J Cardiovasc Electrophysiol. 2016 Mar;27(3):298-302. doi: 10.1111/jce.12876. Epub 2015 Dec 18. PMID: 26538372.
115. Ghanbari H, Jani R, Hussain-Amin A, Al-Assad W, Huether E, Ansari S, Jongnarangsin K, Crawford T, Latchamsetty R, Bogun F, Morady F, Oral H, Chugh A. Role of adenosine after antral pulmonary vein isolation of paroxysmal atrial fibrillation: A randomized controlled trial. Heart Rhythm. 2016 Feb;13(2):407-15. doi: 10.1016/j.hrthm.2015.10.016. Epub 2015 Oct 9. PMID: 26455342.
116. Nakamura K, Naito S, Sasaki T, Nakano M, Minami K, Nakatani Y, Ikeda K, Yamashita E, Kumagai K, Funabashi N, Oshima S. Randomized comparison of contact force-guided versus conventional circumferential pulmonary vein isolation of atrial fibrillation: prevalence, characteristics, and predictors of electrical reconnections and clinical outcomes. J Interv Card Electrophysiol. 2015 Dec;44(3):235-45. doi: 10.1007/s10840-015-0056-7. Epub 2015 Sep 19. PMID: 26387117.
117. Dukkipati SR, Cuoco F, Kutinsky I, Aryana A, Bahnson TD, Lakkireddy D, Woollett I, Issa ZF, Natale A, Reddy VY; HeartLight Study Investigators. Pulmonary Vein Isolation Using the Visually Guided Laser Balloon: A Prospective, Multicenter, and Randomized Comparison to Standard Radiofrequency Ablation. J Am Coll Cardiol. 2015 Sep 22;66(12):1350-60. doi: 10.1016/j.jacc.2015.07.036. PMID: 26383722.
118. Theis C, Konrad T, Mollnau H, Sonnenschein S, Kämpfner D, Potstawa M, Ocete BQ, Bock K, Himmrich E, Münzel T, Rostock T. Arrhythmia Termination Versus Elimination of Dormant Pulmonary Vein Conduction as a Procedural End Point of Catheter Ablation for Paroxysmal Atrial Fibrillation: A Prospective Randomized Trial. Circ Arrhythm Electrophysiol. 2015 Oct;8(5):1080-7. doi: 10.1161/CIRCEP.115.002786. Epub 2015 Aug 21. PMID: 26297786; PMCID: PMC4608486.
119. Luik A, Radzewitz A, Kieser M, Walter M, Bramlage P, Hörmann P, Schmidt K, Horn N, Brinkmeier-Theofanopoulou M, Kunzmann K, Riexinger T, Schymik G, Merkel M, Schmitt C. Cryoballoon Versus Open Irrigated Radiofrequency Ablation in Patients With Paroxysmal Atrial Fibrillation: The Prospective, Randomized, Controlled, Noninferiority FreezeAF Study. Circulation. 2015 Oct 6;132(14):1311-9. doi: 10.1161/CIRCULATIONAHA.115.016871. Epub 2015 Aug 17. PMID: 26283655; PMCID: PMC4590523.
120. Reddy VY, Dukkipati SR, Neuzil P, Natale A, Albenque JP, Kautzner J, Shah D, Michaud G, Wharton M, Harari D, Mahapatra S, Lambert H, Mansour M. Randomized, Controlled Trial of the Safety and Effectiveness of a Contact Force-Sensing Irrigated Catheter for Ablation of Paroxysmal Atrial Fibrillation: Results of the TactiCath Contact Force Ablation Catheter Study for Atrial Fibrillation (TOCCASTAR) Study. Circulation. 2015 Sep 8;132(10):907-15. doi: 10.1161/CIRCULATIONAHA.114.014092. Epub 2015 Aug 10. PMID: 26260733.
121. Macle L, Khairy P, Weerasooriya R, Novak P, Verma A, Willems S, Arentz T, Deisenhofer I, Veenhuyzen G, Scavée C, Jaïs P, Puererfellner H, Levesque S, Andrade JG, Rivard L, Guerra PG, Dubuc M, Thibault B, Talajic M, Roy D, Nattel S; ADVICE trial investigators. Adenosine-guided pulmonary vein isolation for the treatment of paroxysmal atrial fibrillation: an international, multicentre, randomised superiority trial. Lancet. 2015 Aug 15;386(9994):672-9. doi: 10.1016/S0140-6736(15)60026-5. Epub 2015 Jul 23. PMID: 26211828.
122. Raatikainen MJ, Hakalahti A, Uusimaa P, Nielsen JC, Johannessen A, Hindricks G, Walfridsson H, Pehrson S, Englund A, Hartikainen J, Kongstad O, Mortensen LS, Hansen PS; MANTRA-PAF investigators. Radiofrequency catheter ablation maintains its efficacy better than antiarrhythmic medication in patients with paroxysmal atrial fibrillation: On-treatment analysis of the randomized controlled MANTRA-PAF trial. Int J Cardiol. 2015 Nov 1;198:108-14. doi: 10.1016/j.ijcard.2015.06.160. Epub 2015 Jul 4. PMID: 26163901.
123. Faustino M, Pizzi C, Agricola T, Xhyheri B, Costa GM, Flacco ME, Capasso L, Cicolini G, Di Girolamo E, Leonzio L, Manzoli L. Stepwise ablation approach versus pulmonary vein isolation in patients with paroxysmal atrial fibrillation: Randomized controlled trial. Heart Rhythm. 2015 Sep;12(9):1907-15. doi: 10.1016/j.hrthm.2015.06.009. Epub 2015 Jun 5. PMID: 26051530.
124. Cappato R, Marchlinski FE, Hohnloser SH, Naccarelli GV, Xiang J, Wilber DJ, Ma CS, Hess S, Wells DS, Juang G, Vijgen J, Hügl BJ, Balasubramaniam R, De Chillou C, Davies DW, Fields LE, Natale A; VENTURE-AF Investigators. Uninterrupted rivaroxaban vs. uninterrupted vitamin K antagonists for catheter ablation in non-valvular atrial fibrillation. Eur Heart J. 2015 Jul 21;36(28):1805-11. doi: 10.1093/eurheartj/ehv177. Epub 2015 May 14. PMID: 25975659; PMCID: PMC4508487.
125. McLellan AJ, Ling LH, Azzopardi S, Lee GA, Lee G, Kumar S, Wong MC, Walters TE, Lee JM, Looi KL, Halloran K, Stiles MK, Lever NA, Fynn SP, Heck PM, Sanders P, Morton JB, Kalman JM, Kistler PM. A minimal or maximal ablation strategy to achieve pulmonary vein isolation for paroxysmal atrial fibrillation: a prospective multi-centre randomized controlled trial (the Minimax study). Eur Heart J. 2015 Jul 21;36(28):1812-21. doi: 10.1093/eurheartj/ehv139. Epub 2015 Apr 28. PMID: 25920401.
126. Verma A, Jiang CY, Betts TR, Chen J, Deisenhofer I, Mantovan R, Macle L, Morillo CA, Haverkamp W, Weerasooriya R, Albenque JP, Nardi S, Menardi E, Novak P, Sanders P; STAR AF II Investigators. Approaches to catheter ablation for persistent atrial fibrillation. N Engl J Med. 2015 May 7;372(19):1812-22. doi: 10.1056/NEJMoa1408288. PMID: 25946280.
127. Da Costa A, Levallois M, Romeyer-Bouchard C, Bisch L, Gate-Martinet A, Isaaz K. Remote-controlled magnetic pulmonary vein isolation combined with superior vena cava isolation for paroxysmal atrial fibrillation: a prospective randomized study. Arch Cardiovasc Dis. 2015 Mar;108(3):163-71. doi: 10.1016/j.acvd.2014.10.005. Epub 2015 Feb 7. PMID: 25662699.
128. Walfridsson H, Walfridsson U, Nielsen JC, Johannessen A, Raatikainen P, Janzon M, Levin LA, Aronsson M, Hindricks G, Kongstad O, Pehrson S, Englund A, Hartikainen J, Mortensen LS, Hansen PS. Radiofrequency ablation as initial therapy in paroxysmal atrial fibrillation: results on health-related quality of life and symptom burden. The MANTRA-PAF trial. Europace. 2015 Feb;17(2):215-21. doi: 10.1093/europace/euu342. Epub 2015 Jan 6. PMID: 25567068.
129. Kim JS, Shin SY, Na JO, Choi CU, Kim SH, Kim JW, Kim EJ, Rha SW, Park CG, Seo HS, Oh DJ, Hwang C, Lim HE. Does isolation of the left atrial posterior wall improve clinical outcomes after radiofrequency catheter ablation for persistent atrial fibrillation?: A prospective randomized clinical trial. Int J Cardiol. 2015 Feb 15;181:277-83. doi: 10.1016/j.ijcard.2014.12.035. Epub 2014 Dec 11. PMID: 25535691.
130. Atienza F, Almendral J, Ormaetxe JM, Moya A, Martínez-Alday JD, Hernández-Madrid A, Castellanos E, Arribas F, Arias MÁ, Tercedor L, Peinado R, Arcocha MF, Ortiz M, Martínez-Alzamora N, Arenal A, Fernández-Avilés F, Jalife J; RADAR-AF Investigators. Comparison of radiofrequency catheter ablation of drivers and circumferential pulmonary vein isolation in atrial fibrillation: a noninferiority randomized multicenter RADAR-AF trial. J Am Coll Cardiol. 2014 Dec 16;64(23):2455-67. doi: 10.1016/j.jacc.2014.09.053. PMID: 25500229.
131. Atienza F, Almendral J, Ormaetxe JM, Moya A, Martínez-Alday JD, Hernández-Madrid A, Castellanos E, Arribas F, Arias MÁ, Tercedor L, Peinado R, Arcocha MF, Ortiz M, Martínez-Alzamora N, Arenal A, Fernández-Avilés F, Jalife J; RADAR-AF Investigators. Comparison of radiofrequency catheter ablation of drivers and circumferential pulmonary vein isolation in atrial fibrillation: a noninferiority randomized multicenter RADAR-AF trial. J Am Coll Cardiol. 2014 Dec 16;64(23):2455-67. doi: 10.1016/j.jacc.2014.09.053. PMID: 25500229.
132. Zhang XD, Gu J, Jiang WF, Zhao L, Zhou L, Wang YL, Liu YG, Liu X. Optimal rhythm-control strategy for recurrent atrial tachycardia after catheter ablation of persistent atrial fibrillation: a randomized clinical trial. Eur Heart J. 2014 May 21;35(20):1327-34. doi: 10.1093/eurheartj/ehu017. Epub 2014 Feb 3. PMID: 24497338.
133. Kim TH, Park J, Park JK, Uhm JS, Joung B, Hwang C, Lee MH, Pak HN. Linear ablation in addition to circumferential pulmonary vein isolation (Dallas lesion set) does not improve clinical outcome in patients with paroxysmal atrial fibrillation: a prospective randomized study. Europace. 2015 Mar;17(3):388-95. doi: 10.1093/europace/euu245. Epub 2014 Oct 21. PMID: 25336665.
134. Arbelo E, Guiu E, Ramos P, Bisbal F, Borras R, Andreu D, Tolosana JM, Berruezo A, Brugada J, Mont L. Benefit of left atrial roof linear ablation in paroxysmal atrial fibrillation: a prospective, randomized study. J Am Heart Assoc. 2014 Sep 5;3(5):e000877. doi: 10.1161/JAHA.114.000877. PMID: 25193295; PMCID: PMC4323787.
135. Darkner S, Chen X, Hansen J, Pehrson S, Johannessen A, Nielsen JB, Svendsen JH. Recurrence of arrhythmia following short-term oral AMIOdarone after CATheter ablation for atrial fibrillation: a double-blind, randomized, placebo-controlled study (AMIO-CAT trial). Eur Heart J. 2014 Dec 14;35(47):3356-64. doi: 10.1093/eurheartj/ehu354. Epub 2014 Sep 2. PMID: 25182250.
136. McCready J, Chow AW, Lowe MD, Segal OR, Ahsan S, de Bono J, Dhaliwal M, Mfuko C, Ng A, Rowland ER, Bradley RJ, Paisey J, Roberts P, Morgan JM, Sandilands A, Yue A, Lambiase PD. Safety and efficacy of multipolar pulmonary vein ablation catheter vs. irrigated radiofrequency ablation for paroxysmal atrial fibrillation: a randomized multicentre trial. Europace. 2014 Aug;16(8):1145-53. doi: 10.1093/europace/euu064. Epub 2014 May 19. PMID: 24843051; PMCID: PMC4114331.
137. Steinberg JS, Romanov A, Musat D, Preminger M, Bayramova S, Artyomenko S, Shabanov V, Losik D, Karaskov A, Shaw RE, Pokushalov E. Prophylactic pulmonary vein isolation during isthmus ablation for atrial flutter: the PReVENT AF Study I. Heart Rhythm. 2014 Sep;11(9):1567-72. doi: 10.1016/j.hrthm.2014.05.011. Epub 2014 May 12. PMID: 24832767.
138. Di Biase L, Burkhardt JD, Santangeli P, Mohanty P, Sanchez JE, Horton R, Gallinghouse GJ, Themistoclakis S, Rossillo A, Lakkireddy D, Reddy M, Hao S, Hongo R, Beheiry S, Zagrodzky J, Rong B, Mohanty S, Elayi CS, Forleo G, Pelargonio G, Narducci ML, Dello Russo A, Casella M, Fassini G, Tondo C, Schweikert RA, Natale A. Periprocedural stroke and bleeding complications in patients undergoing catheter ablation of atrial fibrillation with different anticoagulation management: results from the Role of Coumadin in Preventing Thromboembolism in Atrial Fibrillation (AF) Patients Undergoing Catheter Ablation (COMPARE) randomized trial. Circulation. 2014 Jun 24;129(25):2638-44. doi: 10.1161/CIRCULATIONAHA.113.006426. Epub 2014 Apr 17. PMID: 24744272.
139. Lin YJ, Chang SL, Lo LW, Hu YF, Chong E, Chao TF, Chung FP, Liao J, Li CH, Tsao HM, Kao T, Chen YY, Huang JL, Chen SA. A prospective and randomized comparison of limited versus extensive atrial substrate modification after circumferential pulmonary vein isolation in nonparoxysmal atrial fibrillation. J Cardiovasc Electrophysiol. 2014 Aug;25(8):803-812. doi: 10.1111/jce.12407. Epub 2014 Apr 9. PMID: 24628987.
140. Han SW, Shin SY, Im SI, Na JO, Choi CU, Kim SH, Kim JW, Kim EJ, Rha SW, Park CG, Seo HS, Oh DJ, Hwang C, Lim HE. Does the amount of atrial mass reduction improve clinical outcomes after radiofrequency catheter ablation for long-standing persistent atrial fibrillation? Comparison between linear ablation and defragmentation. Int J Cardiol. 2014 Jan 15;171(1):37-43. doi: 10.1016/j.ijcard.2013.11.041. Epub 2013 Nov 23. PMID: 24315152.
141. Mont L, Bisbal F, Hernández-Madrid A, Pérez-Castellano N, Viñolas X, Arenal A, Arribas F, Fernández-Lozano I, Bodegas A, Cobos A, Matía R, Pérez-Villacastín J, Guerra JM, Ávila P, López-Gil M, Castro V, Arana JI, Brugada J; SARA investigators. Catheter ablation vs. antiarrhythmic drug treatment of persistent atrial fibrillation: a multicentre, randomized, controlled trial (SARA study). Eur Heart J. 2014 Feb;35(8):501-7. doi: 10.1093/eurheartj/eht457. Epub 2013 Oct 17. PMID: 24135832; PMCID: PMC3930872.
142. Morillo CA, Verma A, Connolly SJ, Kuck KH, Nair GM, Champagne J, Sterns LD, Beresh H, Healey JS, Natale A; RAAFT-2 Investigators. Radiofrequency ablation vs antiarrhythmic drugs as first-line treatment of paroxysmal atrial fibrillation (RAAFT-2): a randomized trial. JAMA. 2014 Feb 19;311(7):692-700. doi: 10.1001/jama.2014.467. Erratum in: JAMA. 2014 Jun 11;311(22):2337. Erratum in: JAMA. 2021 Jul 27;326(4):360. PMID: 24549549.
143. Andrade JG, Khairy P, Macle L, Packer DL, Lehmann JW, Holcomb RG, Ruskin JN, Dubuc M. Incidence and significance of early recurrences of atrial fibrillation after cryoballoon ablation: insights from the multicenter Sustained Treatment of Paroxysmal Atrial Fibrillation (STOP AF) Trial. Circ Arrhythm Electrophysiol. 2014 Feb;7(1):69-75. doi: 10.1161/CIRCEP.113.000586. Epub 2014 Jan 19. PMID: 24446022.
144. Hunter RJ, Berriman TJ, Diab I, Kamdar R, Richmond L, Baker V, Goromonzi F, Sawhney V, Duncan E, Page SP, Ullah W, Unsworth B, Mayet J, Dhinoja M, Earley MJ, Sporton S, Schilling RJ. A randomized controlled trial of catheter ablation versus medical treatment of atrial fibrillation in heart failure (the CAMTAF trial). Circ Arrhythm Electrophysiol. 2014 Feb;7(1):31-8. doi: 10.1161/CIRCEP.113.000806. Epub 2014 Jan 1. PMID: 24382410.
145. Katritsis DG, Pokushalov E, Romanov A, Giazitzoglou E, Siontis GC, Po SS, Camm AJ, Ioannidis JP. Autonomic denervation added to pulmonary vein isolation for paroxysmal atrial fibrillation: a randomized clinical trial. J Am Coll Cardiol. 2013 Dec 17;62(24):2318-25. doi: 10.1016/j.jacc.2013.06.053. Epub 2013 Aug 21. PMID: 23973694.
146. Jones DG, Haldar SK, Hussain W, Sharma R, Francis DP, Rahman-Haley SL, McDonagh TA, Underwood SR, Markides V, Wong T. A randomized trial to assess catheter ablation versus rate control in the management of persistent atrial fibrillation in heart failure. J Am Coll Cardiol. 2013 May 7;61(18):1894-903. doi: 10.1016/j.jacc.2013.01.069. Epub 2013 Mar 7. PMID: 23500267.
147. Wang YL, Liu X, Tan HW, Zhou L, Jiang WF, Gu J, Liu YG. Evaluation of linear lesions in the left and right atrium in ablation of long-standing atrial fibrillation. Pacing Clin Electrophysiol. 2013 Oct;36(10):1202-10. doi: 10.1111/pace.12168. Epub 2013 May 16. PMID: 23678857.
148. Mun HS, Joung B, Shim J, Hwang HJ, Kim JY, Lee MH, Pak HN. Does additional linear ablation after circumferential pulmonary vein isolation improve clinical outcome in patients with paroxysmal atrial fibrillation? Prospective randomised study. Heart. 2012 Mar;98(6):480-4. doi: 10.1136/heartjnl-2011-301107. Epub 2012 Jan 27. PMID: 22285969; PMCID: PMC3285139.
149. Cosedis Nielsen J, Johannessen A, Raatikainen P, Hindricks G, Walfridsson H, Kongstad O, Pehrson S, Englund A, Hartikainen J, Mortensen LS, Hansen PS. Radiofrequency ablation as initial therapy in paroxysmal atrial fibrillation. N Engl J Med. 2012 Oct 25;367(17):1587-95. doi: 10.1056/NEJMoa1113566. PMID: 23094720.
150. Pokushalov E, Romanov A, Corbucci G, Artyomenko S, Turov A, Shirokova N, Karaskov A. Use of an implantable monitor to detect arrhythmia recurrences and select patients for early repeat catheter ablation for atrial fibrillation: a pilot study. Circ Arrhythm Electrophysiol. 2011 Dec;4(6):823-31. doi: 10.1161/CIRCEP.111.964809. Epub 2011 Sep 19. PMID: 21930653.
151. Chen M, Yang B, Chen H, Ju W, Zhang F, Tse HF, Cao K. Randomized comparison between pulmonary vein antral isolation versus complex fractionated electrogram ablation for paroxysmal atrial fibrillation. J Cardiovasc Electrophysiol. 2011 Sep;22(9):973-81. doi: 10.1111/j.1540-8167.2011.02051.x. Epub 2011 May 3. PMID: 21539635.
152. Katritsis DG, Giazitzoglou E, Zografos T, Pokushalov E, Po SS, Camm AJ. Rapid pulmonary vein isolation combined with autonomic ganglia modification: a randomized study. Heart Rhythm. 2011 May;8(5):672-8. doi: 10.1016/j.hrthm.2010.12.047. Epub 2010 Dec 31. PMID: 21199686.
153. Verma A, Mantovan R, Macle L, De Martino G, Chen J, Morillo CA, Novak P, Calzolari V, Guerra PG, Nair G, Torrecilla EG, Khaykin Y. Substrate and Trigger Ablation for Reduction of Atrial Fibrillation (STAR AF): a randomized, multicentre, international trial. Eur Heart J. 2010 Jun;31(11):1344-56. doi: 10.1093/eurheartj/ehq041. Epub 2010 Mar 9. PMID: 20215126; PMCID: PMC2878965.
154. Wilber DJ, Pappone C, Neuzil P, De Paola A, Marchlinski F, Natale A, Macle L, Daoud EG, Calkins H, Hall B, Reddy V, Augello G, Reynolds MR, Vinekar C, Liu CY, Berry SM, Berry DA; ThermoCool AF Trial Investigators. Comparison of antiarrhythmic drug therapy and radiofrequency catheter ablation in patients with paroxysmal atrial fibrillation: a randomized controlled trial. JAMA. 2010 Jan 27;303(4):333-40. doi: 10.1001/jama.2009.2029. PMID: 20103757.
155. Oral H, Chugh A, Yoshida K, Sarrazin JF, Kuhne M, Crawford T, Chalfoun N, Wells D, Boonyapisit W, Veerareddy S, Billakanty S, Wong WS, Good E, Jongnarangsin K, Pelosi F Jr, Bogun F, Morady F. A randomized assessment of the incremental role of ablation of complex fractionated atrial electrograms after antral pulmonary vein isolation for long-lasting persistent atrial fibrillation. J Am Coll Cardiol. 2009 Mar 3;53(9):782-9. doi: 10.1016/j.jacc.2008.10.054. PMID: 19245970.
156. Jaïs P, Cauchemez B, Macle L, Daoud E, Khairy P, Subbiah R, Hocini M, Extramiana F, Sacher F, Bordachar P, Klein G, Weerasooriya R, Clémenty J, Haïssaguerre M. Catheter ablation versus antiarrhythmic drugs for atrial fibrillation: the A4 study. Circulation. 2008 Dec 9;118(24):2498-505. doi: 10.1161/CIRCULATIONAHA.108.772582. Epub 2008 Nov 24. Erratum in: Circulation. 2009 Sep 8;120(10):e83. PMID: 19029470.
157. Nilsson B, Chen X, Pehrson S, Køber L, Hilden J, Svendsen JH. Recurrence of pulmonary vein conduction and atrial fibrillation after pulmonary vein isolation for atrial fibrillation: a randomized trial of the ostial versus the extraostial ablation strategy. Am Heart J. 2006 Sep;152(3):537.e1-8. doi: 10.1016/j.ahj.2006.05.029. PMID: 16923426.
158. Stabile G, Bertaglia E, Senatore G, De Simone A, Zoppo F, Donnici G, Turco P, Pascotto P, Fazzari M, Vitale DF. Catheter ablation treatment in patients with drug-refractory atrial fibrillation: a prospective, multi-centre, randomized, controlled study (Catheter Ablation For The Cure Of Atrial Fibrillation Study). Eur Heart J. 2006 Jan;27(2):216-21. doi: 10.1093/eurheartj/ehi583. Epub 2005 Oct 7. PMID: 16214831.
159. Arentz T, Weber R, Bürkle G, Herrera C, Blum T, Stockinger J, Minners J, Neumann FJ, Kalusche D. Small or large isolation areas around the pulmonary veins for the treatment of atrial fibrillation? Results from a prospective randomized study. Circulation. 2007 Jun 19;115(24):3057-63. doi: 10.1161/CIRCULATIONAHA.107.690578. Epub 2007 Jun 11. PMID: 17562956.
160. Karch MR, Zrenner B, Deisenhofer I, Schreieck J, Ndrepepa G, Dong J, Lamprecht K, Barthel P, Luciani E, Schömig A, Schmitt C. Freedom from atrial tachyarrhythmias after catheter ablation of atrial fibrillation: a randomized comparison between 2 current ablation strategies. Circulation. 2005 Jun 7;111(22):2875-80. doi: 10.1161/CIRCULATIONAHA.104.491530. Epub 2005 May 31. PMID: 15927974.
161. Oral H, Chugh A, Lemola K, Cheung P, Hall B, Good E, Han J, Tamirisa K, Bogun F, Pelosi F Jr, Morady F. Noninducibility of atrial fibrillation as an end point of left atrial circumferential ablation for paroxysmal atrial fibrillation: a randomized study. Circulation. 2004 Nov 2;110(18):2797-801. doi: 10.1161/01.CIR.0000146786.87037.26. Epub 2004 Oct 25. PMID: 15505091.

# Table S1. Justifications adopted to qualify efficacy criteria for clinical or technical/technological conditions as class II recommendations for catheter ablation of AF

**Class II**

**Recommendation Class Ref**

***Evidence for superiority of catheter ablation versus control therapies or***

***technologies/techniques***

- In **paroxysmal AF,** *early (i.e., within 24 months from onset) RF* *ablation*  **II 1**

*is superior to AADs** in preventing progression to persistent AF

study not completed for slow enrollment

- In **paroxysmal AF of first onset,** *RF ablation is superior to AADs** in  **II 2**

reducing or suppressing atrial arrhythmia recurrences

sample size calculation and working hypothesis underwent multiple

revisions during ongoing study

- In **RF ablation of persistent AF,** *adding vein of Marshall ethanol* **II 3**

*infusion is superior to PV isolation only* in reducing atrial arrhythmia

recurrences

symptomatic atrial tachycardia recurrence showed no difference; cannulation

of Vein of Marshall may be technically difficult on large scale; incidence

of complications, especially post-procedural pericarditis and fluid overload,

significantly larger in vein of Marshall arm; attrition [ability to keep

patients in the trial as per randomization; loss of follow-up, not ITT

analysis

- In **symptomatic AF and end-stage heart failure with reduced EF**, **II 4**

*RF* *ablation is superior to guideline-directed medical therapy* in reducing

all-cause death, implantation of a left ventricular assist device or urgent heart

transplantation

single-center; selective reporting of outcomes [risk of bias]

In **paroxysmal and persistent AF,** *ablation is superior to AADs** **II 5**

in improving Quality of Life

lacks sham control

- In **paroxysmal and persistent AF,** *ablation is superior to AADs** **II 6**

in improving psychological symptoms of anxiety and depression

lacks sham control

- In **persistent AF with documented low-voltage atrial areas,** *adding* **II 7**

*ablation of low voltage areas is superior to PV isolation* in reducing

or suppressing atrial arrhythmia recurrences

statistical analysis plan did not include a provision for correcting

for multiplicity; trend towards higher complication in the study group;

- In **drug-refractory paroxysmal and persistent AF,** *ablation is* **II 8**

*superior to AADs** in reducing the risk of future stroke

data from guideline team custom-made meta-analysis

- In **paroxysmal and persistent AF with or without previously II 9**

**failed AADs,** *catheter ablation is superior to AADs** in

- - reducing AF burden
  - reducing all-cause hospitalization
  - improving LVEF
- data from guideline team custom-made meta-analysis
- In **drug-refractory paroxysmal AF,** *Cryo-balloon ablation is* **II 10**

*superior to RF ablation* in reducing re-hospitalization

***Evidence for similarity of efficacy and safety profiles*** ***between comparative***

***technologies or techniques***

- In **drug-refractory paroxysmal AF,** *RF and PF ablation* are similarly  **II 11**

effective in preventing atrial arrhythmia recurrences

early experience with PF ablation, comparative study, 1 death case in the PF

group; as time passes more adverse events are reported, including renal

failure, coronary artery spasm and silent stroke; more time needed before

safety outcomes are collected and reliably compared with those reported

with RF and CB ablation

- In **persistent AF**, *RF and dual-energy PF and RF ablation* are **II 12**

similarly effective in reducing acute procedural failure, atrial

arrhythmia recurrences, drug initiation or escalation or cardioversion

composite outcome; effects on efficacy diluted

**References class II**

1. Kuck KH, Lebedev DS, Mikhaylov EN, Romanov A, Geller L, Kalejs O, Neumann T, Davtyan K, On YK, Popov S, Bongiorni MG, Schluter M, Willems S, Ouyang F. Catheter ablation or medical therapy to delay progression of atrial fibrillation: the randomized controlled atrial fibrillation progression trial (ATTEST). Europace 2021; 23:362-369°. doi:10.1093/euaa298
2. Morillo C, Verma A, Connolly S, Kuck KH, Sterns LD, Beresh H, Healy JS, Natale A for the RAAFT-2 investigators. Radiofrequency ablation vs antiarrhythmic drugs as first-line treatment of paroxysmal atrial fibrillation (RAAFT-2): a randomized trial. JAMA 2014;311:692-699. Doi: 10.001/jama.2014.467
3. Valderrábano M, Peterson LE, Swarup V, Schurmann PA, Makkar A, Doshi RN, DeLurgio D, Athill CA, Ellenbogen KA, Natale A, Koneru J, Dave AS, Giorgberidze I, Afshar H, Guthrie ML, Bunge R, Morillo CA, Kleiman NS. Effect of Catheter Ablation With Vein of Marshall Ethanol Infusion vs Catheter Ablation Alone on Persistent Atrial Fibrillation: The VENUS Randomized Clinical Trial. JAMA. 2020 Oct 27;324(16):1620-1628. doi: 10.1001/jama.2020.16195. PMID: 33107945; PMCID: PMC7592031.
4. Sohns C, Fox H, Marrouche NF, et al. Catheter ablation in end-stage heart failure with atrial fibrillation. New Engl J Med 2023;389:1380-1389 DOI:10.1056/NEJMoa2306037
5. Blomström-Lundqvist C, Gizurarson S, Schwieler J, Jensen SM, Bergfeldt L, Kennebäck G, Rubulis A, Malmborg H, Raatikainen P, Lönnerholm S, Höglund N, Mörtsell D. Effect of Catheter Ablation vs Antiarrhythmic Medication on Quality of Life in Patients With Atrial Fibrillation: The CAPTAF Randomized Clinical Trial. JAMA. 2019 Mar 19;321(11):1059-1068. doi: 10.1001/jama.2019.0335. PMID: 30874754; PMCID: PMC6439911. Other studies?
6. Al-Kaisey AM, Parameswaran R, Bryant C, Anderson R, Hawson J, Chieng D, Segan L, Voskoboinik A, Sugumar H, Wong GR, Finch S, Joseph S , McLellan A, Ling LH , Morton J, Paul Sparks P, n Sanders P, Lee G , Kistler PM, Kalman JM. Atrial Fibrillation Catheter Ablation vs Medical Therapy and Psychological Distress: A Randomized Clinical Trial. JAMA 2023 Sep 12;330(10):925-933.
7. Huo Y, Gaspar T, Schoebauer R, Wojcik M, Fiedler L, Roithinger FX, Martinek M, et al. Low-voltage myocardium-guided ablation trial of persistent atrial fibrillation. NEJM Evid 2022(11). DOI: 10.1056/EVIDoa2200141
8. Providencia R, Ali H, Barra S, Creta A, Kanagaratnam P, Schilling R, Farkowski MM, Cappato R. Catheter ablation for atrial fibrillation and impact on clinical outcomes. Nature Open 2024;00,oeae. doi.org/10.1093/ehjopen/oeae058
9. Providencia R, Ali H, Creta A, Barra S, Kanagaratnam P, Schilling R, Farkowski MM, Cappato R. Impact of catheter ablation of atrial fibrillation on the risk of stroke: A Meta-analysis. Nature Rev 2024; under review
10. Kuck KH, Fürnkranz A, Chun KRJ, Metzner A, Ouyang F, Schlüter M, Elvan A, Lim HW, Kueffer FJ, Arentz T , Albenque JP, Tondo C, Kühne M, Sticherling C, Brugada J ; FIRE AND ICE Investigators Cryoballoon or radiofrequency ablation for symptomatic paroxysmal atrial fibrillation: reintervention, rehospitalization, and quality-of-life outcomes in the FIRE AND ICE trial. Eur Heart J 2016 Oct 7;37(38):2858-2865. doi: 10.1093/eurheartj/ehw285. Epub 2016 Jul 5.
11. Reddy VY, Gerstenfeld EP, Natale A, Whang W, Cuoco FA, Patel C, Mountantonakis SE, Gibson DN, Harding JD, Ellis CR, Ellenbogen KA, DeLurgio DB, Osorio J, Achyutha AB, Schneider CW, Mugglin AS, Albrecht EM, Stein KM, Lehmann JW, Mansour M; ADVENT Investigators. Pulsed Field or Conventional Thermal Ablation for Paroxysmal Atrial Fibrillation. N Engl J Med. 2023 Nov 2;389(18):1660-1671. doi: 10.1056/NEJMoa2307291. Epub 2023 Aug 27. PMID: 37634148
12. Edar et al. Dual-energy lattice-tip ablation system for persistent atrial fibrillation: a randomized trial. Nature Med 2024; doi.org/10.1038/s41591-024-0302

# Table S2 RCTs included in the custom-made meta-analysis elaboration for fulfillment of class II recommendations. a. Study design and baseline characteristics; b. Summary of findings; c. Sub-analyses and sensitivity analyses

**Table S2a**. Summary of Study design and baseline characteristics

| **Study**  **Centres** | **Country** | **N** | **Paroxysmal AF** | **Age** | **Women** | **N of failed AADs** | **BMI (Kg/m^2^)** | **Valvular / HCM** | **CHA_2_DS_2_VASc** | **HF** | **LVEF %** |
| --- | --- | --- | --- | --- | --- | --- | --- | --- | --- | --- | --- |
| CAMTAF 2014  Single-center | UK | Abl 26  Med 24 | 0% (0) | Abl 55±12  Med 60±10 | Abl 3.8% (1)  Med 12.5% (3) | Abl 1 (IQR 0-1)  Med 1 (IQR 0-1) | NA | NA | NA | 100% (50) | <50% |
| RAAFT-AF 2 2014  Multicenter | Canada, USA & Germany | Abl 66  Med 61 | Abl 99% (65)  Med 97% (59) | Abl 56±9  Med 54±12 | Abl 23% (15)  Med 26% (16) | Abl 0  Med 0 | NA | 0% (0) | CHADS_2_  Abl 0 (IQR 0-1)  Med 0 (IQR 0-1) | Abl 3% (2)  Med 2% (1) | >40% |
| AATAC 2016  Multicenter | USA, Italy, France & Czechia | Abl 102  Med 101 | 0% (0) | Abl 62±10  Med 60±11 | Abl 25% (27)  Med 27% (28) | NA | Abl 30±8  Med 29±4 | NA | NA | 100% (203) | ≤40% |
| Sohara et al. 2016  Multicenter | Japan | Abl 100  Med 43 | 100% | Abl 59±10  Med 61±10 | Abl 20% (20)  Med 19% (8) | Refractory to ≥1 class I to IV AAD | NA | NA | CHADS_2_  Abl 1±1  Med 1±1 | NA | Abl 67±6  Med 67±7 |
| CAMERA-MRI 2017  Multicenter | Australia | Abl 33  Med 33 | 0% (0) | Abl 59± 11  Med 62± 9.4 | Abl 6% (2)  Med 12% (4) | Previous use of Amiodarone in  82-91% | Abl 30±7  Med 31±4 | NA | CHA_2_DS_2_VASc  2.4 ±1.0 | 100% (66) | Abl 32±9  Med 34±8 |
| CASTLE-AF 2018  Multicenter | USA, Germany, Netherlands, Hungary & Russia | Abl 179  Med 184 | Abl 30% (54)  Med 35% (64) | Abl 64  (IQR 56-71)  Med 64  (IQR 56-74) | Abl 13% (23)  Med 16% (29) | Previous use of  Amiodarone in  57-60% | Abl 29 (26-32)  Med 29 (26-32) | NA | NA | 100% (363) | ≤35% |
| AMICA 2019  Multicenter | Germany & Hungary | Abl 68  Med 72 | 0% (0) | Abl 65±8  Med 65±8 | Abl 12% (8)  Med 8% (6) | NA | NA | Valvular  Abl 2% (2)  Med 1% (1) | NA | 100% (140) | ≤35% |
| CABANA 2019  Multicenter | USA, Australia, Canada, China, Czechia, Germany, Italy, Korea, Russia & UK | Abl 1108  Med 1096 | Abl 42.4% (470)  Med 43.5% (476) | Abl 68  (IQR 62-72)  Med 67  (IQR 62-72) | Abl 37.3% (413)  Med 37.0% (406) | Previous use of  1 AAD in 82.2%  ≥ 2 AAD in17.8% | Abl 30  (IQR 27-34)  Med 30  (IQR 26-35) | NA | 3  (IQR 2-4) | Abl 34.1% (378)  Med 36.5% (400) | ≤35%  Abl 4.8% (38)  Med 4.2% (31) |
| CAPTAF 2019  Multicenter | Sweden & Finland | Abl 79  Med 76 | 70.9% (56)  75% (57) | Abl 55.8±10.6  Med 56.3±8.9 | Abl 26.6% (21)  Med 18.4% (14) | Previous use of AAD in  Abl 38.0% (30)  Med 44.7% (34) | Abl 27±4  Med 27±4 | Valvular  1.3% (1) | Abl 1 (IQR 0-2)  Med 1 (IQR 0-1) | Abl 2.5% (2)  Med 3.9% (3) | Abl 56±7  Med 56±8 |
| ATTEST 2020  Multicenter | Germany, Russia, Hungary, Latvia, Korea, Italy | Abl 128  Med 127 | 100% | Abl 67.8±4.8  Med 67.6±4.6 | Abl 57.8% (74)  Med 58.2% (74) | Previous use of AAD in  Abl 47.7% (61)  Med 54.3% (69) | NA | 0% HCM | NA | Abl 18.8% (24)  Med 21.3% (21) | Abl 62±6  Med 62±5 |
| STOP AF First 2020  Multicenter | USA | Abl 104  Med 99 | 100% | Abl 60.4±11.2  Med 61.16±11.2 | Abl 39.4% (41)  Med 42.4% (42) | 0% | NA | Valvular  Abl 8% (8)  Med 9% (9) | CHA2DS2-VASc ≥3:  Abl 22% (23)  Med 36.3% (36) | Abl 1% (1)  Med 3% (3) | Abl 61±6  Med 61±6 |
| EARLY-AF 2021  Multicenter | Canada | Abl 154  Med 149 | Abl 95.5% (147)  Med 94.0% (140) | Abl 57.5±12.3  Med 59.5±10.6 | Abl 27.2% (42)  Med 31.5% (47) | Remote/irregular or under-dosed:  Abl 26% (40)  Med 29.5% (44) | Abl 31±14  Med 30±9 | NA / Excluded  *LVH >18mm, >moderate-severe MR, prothesic valves* | Abl 1.9±1.0  Med 1.9±1.1 | Abl 9.1% (14)  Med 9.4% (14) | Abl 60±7  Med 60±8 |
| CAPA 2021  Multicenter | China | Abl 327  Med 321 | 0% | Abl 64.8±12.6  Med 64.4±13.6 | Abl 33.3% (109)  Med 36.7% (118) | Previous use of Amiodarone in  Abl 73.7% (241)  Med 68.8% (221) | NA | NA / Excluded HCM | Abl 2.0±0.9  Med 2.1±1.0 | 0% | Abl 53±9  Med 52±9 |
| Cryo-FIRST 2021  Multicenter | Germany, Italy, Belgium, Croatia, France, Norway & USA | Abl 107  Med 111 | 100% | Abl 50.5±13.1  Med 54.1±13.4 | Abl 28.9% (31)  Med 35.1% (39) | 0% | NA | Valvular  Abl 2.8% (3)  Med 1.8% (2)  HCM 0% | CHA_2_DS_2_VASC  ≥3:  Abl 6.5% (7)  Med 10.8% (12) | 0% | Abl 63±5  Med 64±5 |
| RAFT-AF 2022  Multicenter | Canada, Brazil, Sweden & Taiwan | Abl 214  Med 197 | Abl 8.9% (19)  Med 5.6% (11) | Abl 65.9±8.6  Med 67.5±8.0 | Abl 26.6% (57)  Med 24.9% (49) | Previous use of AAD in  Abl 43.9% (94)  Med 39.1% (77) | Abl 30±7  Med 31±7 | NA  Excluded Severe valvular or rheumatic | CHA_2_DS_2_VASC  ≥3:  Abl 65.8% (141)  Med 70.5% (139) | 100% | ≤45% in  Abl 57.9% (124)  Med 58.9% (116) |
| AVATAR 2022  Single-center | UK | Abl 218  Med 103 | 100% | Abl 59.9±10.6  Med 60.5±10.3 | Abl 40.4% (88)  Med 44.6% (46) | Previous use of AAD in 1/3 | Abl 29±5  Med 28±5 | NA | CHA_2_DS_2_VASC  ≥3:  Abl 16.0% (35)  Med 22.5% (23) | NA | Abl 58±5  Med 58±6 |
| CASTLE-HTx 2023  Single-center | Germany | Abl 97  Med 97 | Abl 29% (28)  Med 32% (31) | Abl 62±12  Med 65±10 | Abl 12.3% (12)  Med 25.7% (25) | Previous use of Amiodarone in  Abl 45% (44)  Med 47% (46) | Abl 28±4  Med 28±5 | NA | NA | 100% (endstage pre-transplant) | Abl 29±6  Med 25±6 |
| Chieng et al. 2023  Single-center | Australia | Abl 16  Med 15 | Abl 18.8% (3)  Med 20% (3) | Abl 65.5±7.6  Med 66.7±7.9 | Abl 50% (8)  Med 53.3% (8) | Previous use of AAD in  Abl 68.75% (11)  Med 66.66% (10) | Abl 31±6  Med 32±4 | 0% / excluded | CHA_2_DS_2_VASC  Abl 3±2  Med 4±2 | 100%  (HFPEF) | Abl 60±5  Med 59±5 |

**Table S2b**. Summary of Findings Table

| **Outcome** | **Effect Size**  **95%CI**  **P** | **Studies**  **Sample size** | **NNT to prevent one event** | **Heterogeneity**  **Risk of Bias Assessment** | **Indirectness**  **Imprecision**  **Publication Bias** | **Interpretation**  **Quality of Evidence/GRADE** |
| --- | --- | --- | --- | --- | --- | --- |
| Stroke | RR=0.63  0.45-0.89  P=0.008 | 12 RCTs,  5418 patients | 79.0 patients  1.8% vs. 3.1%  52/2815 vs. 81/2603 | Low heterogeneity (I^2^=0%)  RoB – ↓1 level  (Selection) | No indirectness  No Imprecision  No Publication Bias | Significant reduction in Stroke  Moderate Quality  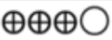 |
| All-Cause Mortality | RR= 0.69  0.57-0.85  P=0.0003 | 16 RCTs,  5871 patients | 42.2 patients  4.7% vs. 7.1%  143/3016 vs. 203/2855 | Low heterogeneity (I^2^=12%)  RoB – ↓1 level  (Selection) | No indirectness *  No Imprecision  No Publication Bias | Significant reduction in All-cause Mortality  Moderate Quality  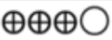 |
| Cardiovascular Mortality | RR=0.55  0.34-0.87  P=0.01 | 8 RCTs  3732 patients | 46.1 patients  2.7% vs. 4.8%  50/1872 vs. 90/1860 | Low heterogeneity (I^2^=33%)  RoB – ↓1 level  (Selection & Performance) | No indirectness *  No Imprecision  N.A. (<10 RCT) | Significant reduction in Cardiovascular Mortality  Moderate Quality  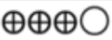 |
| All-Cause Hospitalizations | RR=0.61  0.41-0.89  P=0.01 | 7 RCTs  1870 patients | 8.5 patients  20.6% vs. 32.3%  207/1004 vs. 280/866 | High heterogeneity (I^2^=75%)  RoB – ↓1 level  (Selection & Performance) | No indirectness  No Imprecision  N.A. (<10 RCT) | Significant reduction in All-Cause Hospitalizations  Low Quality  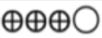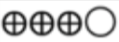 |
| Cardiovascular hospitalizations | RR=0.83  0.71-0.97  P=0.02 | 5 RCTs  3365 patients | 11.6 patients  38.1% vs. 46.4%  663/1752 vs. 749/1613 | Low heterogeneity (I^2^=29%)  RoB – ↓1 level  (Selection & Performance) | No indirectness *  No Imprecision  N.A. (<10 RCT) | Significant reduction in Cardiovascular hospitalizations  Moderate Quality  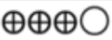 |
| Heart Failure hospitalizations | RR=0.71  95%CI 0.54-0.94  P=0.02 | 4 RCTs  1618 patients | 19.2 patients  13.6% vs. 18.8%  109/804 vs. 153/814 | Low heterogeneity (I^2^=29%)  RoB – ↓1 level  (Selection & Performance) | No indirectness *  No Imprecision  N.A. (<10 RCT) | Significant reduction in Heart Failure hospitalizations  Moderate Quality  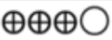 |
| AF Burden | MD=14.9  2.0-27.8  P=0.02 | 4 RCTs  615 patients | - | High heterogeneity (I^2^=91%)  RoB – ↓1 level  (Selection) | No indirectness  No Imprecision  N.A. (<10 RCT) | Significant reduction in AF burden  Low Quality  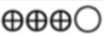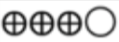 |
| AF relapse | RR=0.26  95%CI 0.12-0.57  P=0.0007 | 16 RCTs  4751 patients | 2.9 patients  35.4% vs. 70.0%  876/2473 vs. 1595/2278 | High heterogeneity (I^2^=92%)  RoB – ↓1 level  (Selection & Performance) | No indirectness  No Imprecision  Publication Bias | Significant reduction in AF relapse  Low Quality  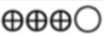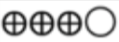 |
| LVEF change | MD=5.65  95%CI 3.45-7.85  P<0.00001 | 7 RCTs  1121 patients  FUP – range: | - | High heterogeneity (I^2^=88%)  RoB – ↓1 level  (Selection & Performance) | No indirectness *  No Imprecision  N.A. (<10 RCT) | Significant improvement in LV ejection fraction  Low Quality  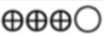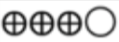 |
| Quality of Life  MLHFQ  △ over 12 months  AFEQT  △ over 12 months  SF-36  at 12 months | MD=-7.96  -12.88 to  -3.04  P=0.002  MD=6.98  4.80-9.17  P<0.0001  MD=3.54  1.23-5.84  P=0.003 | 5 RCTs  653 patients  7 RCTs  3206 patients  4RCTs  2256 patients | - | Moderate heterogeneity (I^2^=68%)  High heterogeneity (I^2^=96%)  High heterogeneity (I^2^=67%)  RoB – ↓1 level  (Selection & Performance) | No indirectness  No Imprecision  N.A. (<10 RCT) | Significant improvement in Quality of Life  Low Quality  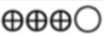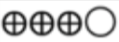 |

**Table S2c.** Sub-analyses and Sensitivity Analyses

|  | **N Trials, Patients** | **Stroke** | **All-Cause mortality** | **All-Cause Hospital.** | **AF Relapse** |
| --- | --- | --- | --- | --- | --- |
| HF | 9  2260 | RR=0.60  0.28-1.29  P=0.19  I2=0% | RR=0.61  0.48-0.77  P<0.0001  I2=0% | RR=0.71  0.48-1.06  P=0.10  I2=77% | RR=0.38  0.25-0.58  P<0.00001  I2=93% |
| No HF | 9  3611 | RR=0.64  0.43-0.93  P=0.02  I2=0% | RR=1.08  0.71-1.64  P=0.72  I2=0% | RR=0.42  0.26-0.67  P=0.0003  I2=0% | RR=0.57  0.45-0.73  P<0.00001  I2=92% |
| Paroxysmal AF | 6  1382 | RR=0.65  0.11-3.93  P=0.64  I2=0% | RR=0.77  0.12-4.76  P=0.78  I2=32% | RR=0.42  0.26-0.67  P=0.0003  I2=0% | RR=0.53  0.47-0.59  P<0.00001  I2=85% |
| Persistent AF | 6  1573 | RR=0.63  0.35-1.16  P=0.14  I2=0% | RR=0.72  0.51-1.02  P=0.07  I2=0% | RR=0.61  0.45-0.84  P=0.002  I2=23% | RR=0.32  0.28-0.37  P<0.00001  I2=90% |
| Mixed Parox/Pers | 4  2916 | RR=0.63  0.41-0.96  P=0.03  I2=0 | RR=0.59  0.36-0.97  P=0.04  I2=65% | RR=0.96  0.83-1.12  P=0.60  I2=NA | RR=0.66  0.61-0.71  P<0.00001  I2=91% |
| Early Ablation | 4  851 | RR=0.32  0.01-7.86  P=0.49  I2=NA | NA | RR=0.38  0.23-0.63  P=0.0002  I2=0% | RR=0.65  0.55-0.76  P<0.00001  I2=3% |
| Ablation after AAD failure | 12  5020 | RR=0.63  0.45-0.89  P=0.009  I2=0% | RR=0.72  0.59-0.89  P=0.003  I2=19% | RR=0.72  0.50-1.04  P=0.08  I2=69% | RR=0.43  0.32-0.56  P<0.00001  I2=94% |
| Higher quality studies  (≤1 high risk of bias domain) | 15  5223 | RR=0.64  0.43-0.95  P=0.03  I2=0% | RR=0.67  0.52-0.86  P=0.002  I2=19% | RR=0.61  0.41-0.89  P=0.010  I2=75% | RR=0.47  0.37-0.60  P<0.00001  I2=92% |
| Lower Quality  (≥2 high risk of bias domains) | 2  791 | RR=0.60  0.31-1.18  P=0.14  I2=0% | RR=0.98  0.29-3.36  P=0.98  I2=NA | NA | RR=0.42  0.35-0.50  P<0.00001  I2=NA |
| Studies published  < 5 years ago | 11  5062 | RR=0.64  0.44-0.92  P=0.02  I2=0% | RR=0.78  0.60-1.01  P=0.06  I2=5% | RR=0.55  0.37-0.83  P=0.004  I2=29% | RR=0.45  0.34-0.61  P<0.00001  I2=94% |
| Studies published  ≥ 5 years ago | 4  809 | RR=0.55  0.21-1.47  P=0.23  I2=0% | RR=0.51  0.34-0.75  P=0.0006  I2=0% | RR=0.68  0.28-1.24  P=0.21  I2=84% | RR=0.51  0.39-0.67  P<0.00001  I2=75% |

# Table S3. Main characteristics and outcomes of RCTs investigating the role of supplementary linear lesion ablation for the treatment of AF

| **1^st^ Author**  **(Trial name)** | **Journal/year** | **Study design** | **Pt n°** | **Parox**  **AF%** | **Approach**  **Open irrigated tip nearly all*** | **I° endpoint** | **Follow-up duration**  **Methodology** | **Outcomes** | **Complications** | **Comments** |
| --- | --- | --- | --- | --- | --- | --- | --- | --- | --- | --- |
| Gaita F^1^ | Circ AE/2008 | RCT | 204 | 61% | PVI vs PVI plus linear lesions (roof and mitral isthmus); 1:2 random assignment; CTI ablation for all | SR maintenance w/o AADs; palpitations lasting >30 seconds or documented AT/AF > 30s | 3 yrs; 24hr Holter @1,3,6,12,18,24 mnths and then 6 monthly | PAF: SR in 29% PVI only vs 53% PVI+LL, log rank test of KM estimates significant  PeAF; SR in 19% PVI only vs 41% PVI+LL,  PVI+LL more effective than PVI only for 36mnth f-up for all combined; p=0.0014 | Tamponade in 2, TIA in 2, | Rather low rate (31%) of complete block at the mitral isthmus since no CS ablation;  Higher efficacy of PVI + LL in both PAF and PeAF @ 3yrs but not in PAF @ 12months  Larger LA+WACA |
| Fassini G^2^ | JCE 2005 | RCT | 187 | 67% | PV disconnection (PVD) vs PVD and mitral isthmus line | First AF recurrence or Rate of sinus rhythm maintenance at 1 yr | 1 yr; 2 months twice daily telephonic ECG, 24h Holter @ 1,3,6,9 & 12 mnths | Combined PAF&PeAF: 53+/-5 vs 71+/-5% (PVD+MIL) SR@1yr; p=0.01  PAF: 64 vs 76%; PeAF: 36 vs 74% | 1 tamponade, 1 TIA, | 76% MI block, 3 atypical flutter with incomplete MI block; fluoroscopic guidance;  Ostial Lasso guided PVI |
| Hocini M^3^ | Circulation 2005 | RCT | 90 | 100% | PV isolation vs PV isolation with linear lesion joining superior PVs | Freedom from flutter or AF without antiarrhythmic drugs | 15+/-4 mnths, Exercise testing & 48hr Holter 1,3,6,12 mnths | 87% with roofline vs 69% with PVI alone arrhythmia free w/o AADs, p=0.04 | 1 tamponade, 1 phrenic nerve injury with complete recovery, I asymptomatic 70% PV stenosis | Additional roofline prolonged AF CL, terminated AF, rendered it non-inducible and provided improved clinical outcome; 3 patients developed perimitral flutter;  Ostial, circumferential PVI |
| Willems S^4^ | European Hrt J, 2006 | RCT with interim analysis | 100 planned; 62 – early termination by interim analysis | 0 | PVI alone (+/-CTI ablation) vs supplementary roof and mitral isthmus line | AF/flutter on Tele-ECG daily and in case of symptoms | 1 yr for all, median 487 days | Freedom from AF/Flutter 20% PVI alone vs 69% with supplementary linear LA ablation; | 1 tamponade, 1 minor ischemic stroke | Relatively small sample size, large effect difference.  44% roof and 72% MI block – no CS ablation.  incomplete LA lines associated with high recurrence rate  PeAF and non-circumferential PVI |
| Shaikh I^5^ | JICE 2006 | RCT | 100 | 100% | PVI alone vs supplementary roof and mitral line | freedom from AF after single ablation procedure, with or without antiarrhythmics | 9 months | 86% (+LLs) vs 58% (PVI) freedom from AF at 1 month; 90 vs 82% at 9 months.  54% & 62% on AADs | 1 tamponade, 1 TIA, 1 pericarditis: all in PVI only group | Non-irrigated catheter, no evaluation of conduction block across LLs, Holters only for symptoms |
| Mun HS^6^ | Heart 2012 | RCT | 156 | 100% | PVI alone vs PVI+roofline vs PVI+post box lesion | Procedure time, ablation time and freedom from AF/AT recurrence>30s; | 15.6+/-5 Holters/event recorders at 3,6 & 12 mnths mnths | Recurrence in 11.5% in CPVI, 21.2% in CPVI+RL and 19.2% in CPVI+PostBox @ 3 mnths, p=ns; KM curve log rank p=ns als0 | 1 TIA, 1 pericardial effusion, 4 pericarditis | Roof line block: 81%; box: 60%;  WACA+small LA+ young age? To explain the absence of difference |
| Arbelo E^7^ | JAHA 2014 | RCT | 120 | 100% | PVI+roofline vs PVI alone | any atrial tachyarrhythmia lasting ≥30 seconds: | 1,3&6 mnth Holters, mean fup 15+/-10 mnths | Freedom from any AT/AF: 59% PVI+RL vs. PVI: 56% at 12 months; log rank P=0.877 | 1 Tamponade, 1 mitral chordal rupture (entrapped Lasso; emergency surgery), 1 pericarditis, 1 TIA | WACA, Young patients, small LA, as above.  81% roof line block: no difference in outcomes with vs w/o block |
| Verma A8 | NEJM 2015 | Multicentric international RCT | 589 | 0 | 1:4:4 ratio randomisation to PVI only, PVI+CFAE, PVI +LL (roof and mitral isthmus lines) | freedom from documented AF recurrence longer than 30 seconds after single ablation procedure, with or w/o AADs | 3, 6-, 9-, 12-, and 18-month Holters + trans-telephonic ECG for symptoms (all 18 months) | 59% PVI only, 49% PVI+CFAE, 46% PVI+LLs at 18 mnths; p=0.15 | 3 tamponades, 3 TIAs, 1 atrio-esophageal fistula | Larger LA, older cohort compared to preceding series;  74% conduction block of LLs; PVI alone group underpowered |
| Wynn G^9^ | Heart Rhythm 2016 | Multicentric international RCT | 122 | 39% | PVI alone vs PVI + roof line + mitral isthmus line + CTI line | atrial tachyarrhythmia recurrence lasting >30 seconds | Holter @ 3, 6, and 12 months and according to symptoms, 12 mnth fup | 38% PVI + lines group and 32% PVI-only group (P >0 .50) | 1 pericardial effusion requiring drainage, 2 fem pseudoaneurysms, 5 fem hematomas, 2 hrt failures | Older and larger LA and higher CHADSVASC vs older studies;  Non-irrigated cath also used; CF also!  WACA+ LL block in 90% roofline, 83% MI& 96% CTI |
| Yu HT^10^ | CircAE 2017 | Multicentric RCT | 113 | PeAF to PAF with amio, 100% | PVI alone vs PVI + roofline + post inf line + ant line | any documented AF or AT >/= 30 s with or without AAD | 24-hour Holter recording at 3, 6 and every 6 months, 18.6+/-11.4 mnths fup | 19%PVI only vs 31% PVI+ lines clinical recurrence rate | 1 tamponade, 1 fem AV fistula, 1 AE fistula (dcd) | No difference in intention to treat outcomes; but PVI + lines more effective when complete block (subgroup) |

**References: Linear Lesion ablation**

1. Gaita F, Caponi D, Scaglione M, Montefusco A, Corleto A, Di Monte F, Coin D, Di Donna P, Giustetto C. Long-term clinical results of 2 different ablation strategies in patients with paroxysmal and persistent atrial fibrillation. Circ Arrhythm Electrophysiol 2008;1(4):269-75. doi: 10.1161/CIRCEP.108.774885.
2. Fassini G, Riva S, Chiodelli R, Trevisi N, Berti M, Carbucicchio C, Maccabelli G, Giraldi F, Della Bella P. Left mitral isthmus ablation associated with PV Isolation: long-term results of a prospective randomized study. J Cardiovasc Electrophysiol 2005;16(11):1150-6. doi: 10.1111/j.1540-8167.2005.50192.x.
3. Hocini M, Jaïs P, Sanders P, Takahashi Y, Rotter M, Rostock T, Hsu L, Sacher F, Reuter S, Clémenty J, Haïssaguerre M. Techniques, evaluation, and consequences of linear block at the left atrial roof in paroxysmal atrial fibrillation: a prospective randomized study. Circulation 2005;112(24):3688-96. doi: 10.1161/CIRCULATIONAHA.105.541052.
4. Willems S, Klemm H, Rostock T, Brandstrup B, Ventura R, Steven D, Risius T, Lutomsky B, Meinertz T. Substrate modification combined with pulmonary vein isolation improves outcome of catheter ablation in patients with persistent atrial fibrillation: a prospective randomized comparison. Eur Heart J 2006;27(23):2871-8. doi: 10.1093/eurheartj/ehl093.
5. Sheikh I, Krum D. Cooley R, Dhala A, Blanck Z, Bhatia A, Nangia V, Akhtar M, Sra J. Pulmonary vein isolation and linear lesions in atrial fibrillation ablation. J Interv Card Electrophysiol (2006) 17:103–109 DOI 10.1007/s10840-006-9066-9.
6. Mun H, Joung B, Shim J, Hwang H, Kim J, Lee M, Pak H. Does additional linear ablation after circumferential pulmonary vein isolation improve clinical outcome in patients with paroxysmal atrial fibrillation? Prospective randomised study. Heart 2012; 98:480-484. doi:10.1136/heartjnl-2011-301107.
7. Arbelo E, Guiu E, Bisbal F, Ramos P, Borras R, Andreu D, Tolosana J M, Berruezo A, Brugada J, Mont L. Benefit of Left Atrial Roof Linear Ablation in Paroxysmal Atrial Fibrillation: A Prospective, Randomized Study . J Am Heart Assoc. 2014;3:e000877 doi:10.1161/JAHA.114.000877.
8. Verma A, Jiang C, Betts TR, Chen J, Deisenhofer I, Mantovan R, Macle L, Morillo CA, Haverkamp W, Weerasooriya R, Albenque JP, Nardi S, Menardi E, Novak P, Sanders P; STAR AF II Investigators. Approaches to catheter ablation for persistent atrial fibrillation. N Engl J Med 2015;372(19):1812-22. doi: 10.1056/NEJMoa1408288.
9. Wynn GJ, Panikker S, Morgan M, Hall M, Waktare J, Markides V, Hussain W, Salukhe T, Modi S, Jarman J, Jones DG, Snowdon R, Todd D, Wong T, Gupta D. Biatrial linear ablation in sustained nonpermanent AF: Results of the substrate modification with ablation and antiarrhythmic drugs in nonpermanent atrial fibrillation (SMAN-PAF) trial. Heart Rhythm 2016;13(2):399-406. doi: 10.1016/j.hrthm.2015.10.006.
10. Yu HT, Shim J, Park J, Kim IS, Kim TH, Uhm JS, Joung B, Lee MH, Kim YH, Pak HN. Pulmonary Vein Isolation Alone Versus Additional Linear Ablation in Patients With Persistent Atrial Fibrillation Converted to Paroxysmal Type With Antiarrhythmic Drug Therapy: A Multicenter, Prospective, Randomized Study. Circ Arrhythm Electrophysiol 2017 Jun;10(6):e004915. doi: 10.1161/CIRCEP.116.004915.

# Table S4. Main characteristics and outcomes of RCTs investigating the role of supplementary CFAE for the treatment of AF

| **1st Author**  **(Trial name)** | **Journal/year** | **Study design** | **Pt n°** | **Parox**  **AF%** | **Approach**  **Open irrigated tip except *** | **I° endpoint** | **Follow-up duration**  **Methodology** | **Outcomes** | **Complications** | **Comments** |
| --- | --- | --- | --- | --- | --- | --- | --- | --- | --- | --- |
| Oral H1 | Circulation 2004 | RCT | 100 of whom 60 randomised | 100% | *, 8 mm tip,  Circumferential PVI vs PVI plus ‘lines’ through CFAE electrograms: septum, roof, posterior mitral annulus, and/or anterior wall  CFAE: ‘fractionated or rapid atrial activity’ | Documented AF | 8+/-6 mnths, symptoms suggestive of AF, in which case an event recorder was used to document the rhythm | Freedom from AF recurrence w/o AAD 85% PVI + additional LA ablation vs 67% PVI only @6 mnths; p=0.02 | LA flutter, 27% vs 19% PVI only; p=0.4; | Non-inducibility used to subgroup; No evaluation of conduction block on the lines; 8 mm catheter used; no evaluation of asymptomatic recurrences; study may be evaluated primarily as CFAE ablation despite ‘lines’ |
| Diesenhofer I2 | JCE 2009 | RCT | 98 | 100% | PVI vs PVI+CFAE  CFAE: ‘following the description of Nademanee et al’ | freedom of atrial tachyarrhythmia >30 seconds on 7 day Holter and freedom of symptomatic AF/atrial tachycardia (AT) @3 months | Symptoms at 1,3,6,9,12 mnth; 24h holter @1 mnth, 7 day @ 3 mnths | 75% PVI alone vs 76% PVI + free of recurrences @ 3month Holter; | 1 tamponade, 1 asystole requiring resuscitation, 1 symptomatic <50% PV stenosis | Subgroup analysis @19+/-6 mnths: better outcomes for post PVI inducible AF with CFAE ablation who became non-inducible |
| Elayi CS3 | Heart Rhythm 2008 | Multicenter RCT | 144 | 0 (LSPeAF) | CPVA vs PVAI vs CFAE+PVAI  CFAE: ‘regions with a mean CL of less than 120 ms’ | Freedom of AF/AT>1min | Event recorder 4 times a week for 6 months, 48h holter 1,3, 6, 9, 12, 15 months | 11% CPVA vs 40% PVAI vs 61% PVAI+CFAE @ 16 mnths; p<0.001 | 2 pericardial effusions requiring drainage; 2 asymptomatic PV stenoses | Biatrial CFAE ablated |
| Oral H4 | JACC 2009 | RCT | 119; 19 terminated with PVAI; 100 randomised to CVEE vs CFAE ablation | 0 | PVAI for all; those non- terminated with PVAI randomised to CVEE vs CFAE ablation  CFAE def: CL<120 ms or < than AF CL in the CS, or fractionated or continuous electrical activity | freedom from (symptomatic + asymptomatic) AF/AT >30s w/o AAD after a single ablation procedure. | Visit @ 3 months, then 3-6 monthly: auto-triggered event recorder from 6 months | @10+/-3 months after single procedure 38% (PVAI only) vs 36% (+CFAE) in SR w/o AADs, p=0.84 | 2 transient pericarditis, 1 small effusion, 1 extraperitoneal bleed, 1 AV fistula | No additional benefit of CFAE after PVAI; irrigated tip used,  Visual CFAE definition, CS and LA targeted not RA,  Lower power on posterior wall  Repeat ablations >90% for recurrent AF |
| Di Biase L5 | Circ AE 2009 | Multicenter RCT | 103 | 100 | PVAI vs CFAE biatrial vs PVAI+CFAE  CFAE: 2 deflections or more or with fractionated baseline complexes with continuous activity over 10s; a cycle length <120 ms over 10s | Freedom from AF/AT with or without AADs > 1 minute | Visit @ 3months then 3 monthly, event recorder for 5 months; to record 4/week; 8-hour Holter monitor was obtained at 3, 6, 9, 12, and 15 months | @ 1yr, 89%, 23% and 91% freedom | None | CFAE alone least effective; PVAI alone as effective as PVAI+CFAE |
| Verma A6 | EHJ 2010 | Multicenter RCT | 100 | 64 | CFE vs PVI vs PVI+CFE  CFAE: Automated Ensite algorithm, mean CL<120ms. | freedom from AF/AT >30s recurrence | ECG & 48 h Holter @ 3, 6, and 12 months | PVI+CFE (74%) freedom from AF compared with PVI (48%) and CFE (29%) (P< 0.004) @12 mnth | 2 tamponades, 4 minor bleeding, 1 pseudo-aneurysm | CFE ablation useful adjuvant; Completely contradictory results vs Star II; results significant in PeAF subgroup |
| Chen M7 | JCE 2011 | RCT, 2 center? | 118 | 100 | CFAE vs PVAI followed by crossover in case of spontaneous/inducible AF  CFAE: Automated Ensite algorithm, mean CL<120ms. | Freedom from AF/AT>30s | 24h Holter @ 3 days, 1, 3, 6, and 12 months | 22.6 ± 6.4 months fup  AF persisted/inducible in 24/59 (41%)PVAI, and 34/58 (59%) CFE (P = 0.05); CFE ablation alone (38%) had significantly lower overall success rate vs PVAI or PVAI vs CFAE | 2 tamponade,2 hemothorax | CFE ablation alone (38%) had significantly lower overall success rate AF/AT; CFE ablation in PAF patients was associated with higher occurrence rate of postprocedure AT compared with PVAI ablation |
| Verma A8 | NEJM 2015 | Multicentric international RCT | 589 | 0 | 1:4:4 ratio randomisation to PVI only, PVI+CFAE, PVI +LL (roof and mitral isthmus lines)  CFAE: Automated Ensite algorithm, mean CL<120ms. | freedom from documented AF recurrence longer than 30 seconds after single ablation procedure, with or w/o AADs | 3, 6-, 9-, 12-, and 18-month Holters + trans-telephonic ECG for symptoms (all 18 months) | 59% PVI only, 49% PVI+CFAE, 46% PVI+LLs at 18 mnths; p=0.15 | 3 tamponades, 3 TIAs, 1 atrio-esophageal fistula | Larger LA, older cohort compared to preceding series;  74% conduction block of LLs; PVI alone group underpowered  244 patients with lines, 244 with CFAE ablation  CFAEs eliminated (LA, CS, RA) in 80% |

**References: CFAE**

1. Oral H, Chugh A, Lemola K, Cheung P, Hall B, Good E, Han J, Tamirisa K, Bogun F, Pelosi F Jr, Morady F. Noninducibility of atrial fibrillation as an end point of left atrial circumferential ablation for paroxysmal atrial fibrillation: a randomized study. Circulation. 2004;110(18):2797-801. doi: 10.1161/01.CIR.0000146786.87037.26.
2. Deisenhofer I, Estner H, Reents T, Fichtner S, Bauer A, Wu J, Kolb C, Zrenner B, Schmitt C, Hessling G. Does electrogram guided substrate ablation add to the success of pulmonary vein isolation in patients with paroxysmal atrial fibrillation? A prospective, randomized study. J Cardiovasc Electrophysiol. 2009;20(5):514-21. doi: 10.1111/j.1540-8167.2008.01379.x.
3. Elayi CS, Verma A, Di Biase L, Ching CK, Patel D, Barrett C, Martin D, Rong B, Fahmy TS, Khaykin Y, Hongo R, Hao S, Pelargonio G, Dello Russo A, Casella M, Santarelli P, Potenza D, Fanelli R, Massaro R, Arruda M, Schweikert RA, Natale A. Ablation for longstanding permanent atrial fibrillation: results from a randomized study comparing three different strategies. Heart Rhythm 2008;5(12):1658-64. doi: 10.1016/j.hrthm.2008.09.016.
4. Oral H, Chugh A, Yoshida K, Sarrazin JF, Kuhne M, Crawford T, Chalfoun N, Wells D, Boonyapisit W, Veerareddy S, Billakanty S, Wong WS, Good E, Jongnarangsin K, Pelosi Jr F, Bogun F, Morady F. A randomized assessment of the incremental role of ablation of complex fractionated atrial electrograms after antral pulmonary vein isolation for long-lasting persistent atrial fibrillation. J Am Coll Cardiol 2009;53(9):782-9. doi: 10.1016/j.jacc.2008.10.054.
5. Di Biase L, Elayi CS, Fahmy TS, Martin DO, Ching CK, Barrett C, Bai R, Patel D, Khaykin Y, Hongo R, Hao S, Beheiry S, Pelargonio G, Dello Russo A, Casella M, Santarelli P, Potenza D, Fanelli R, Massaro R, Wang P, Al-Ahmad A, Arruda M, Themistoclakis S, Bonso A, Rossillo A, Raviele A, Schweikert RA, Burkhardt DJ, Natale A. Atrial fibrillation ablation strategies for paroxysmal patients: randomized comparison between different techniques. Circ Arrhythm Electrophysiol. 2009;2(2):113-9. doi: 10.1161/CIRCEP.108.798447.
6. Verma A, Mantovan R, Macle L, De Martino G, Chen J, Morillo CA, Novak P, Calzolari V, Guerra PG, Nair G, Torrecilla EG, Khaykin Y. Substrate and Trigger Ablation for Reduction of Atrial Fibrillation (STAR AF): a randomized, multicentre, international trial. Eur Heart J 2010;31(11):1344-56. doi: 10.1093/eurheartj/ehq041.
7. Chen M, Yang B, Chen H, Ju W, Zhang F, Tse HF, Cao K. Randomized comparison between pulmonary vein antral isolation versus complex fractionated electrogram ablation for paroxysmal atrial fibrillation. J Cardiovasc Electrophysiol. 2011;22(9):973-81. doi: 10.1111/j.1540-8167.2011.02051.x.
8. Verma A, Jiang C, Betts TR, Chen J, Deisenhofer I, Mantovan R, Macle L, Morillo CA, Haverkamp W, Weerasooriya R, Albenque JP, Nardi S, Menardi E, Novak P, Sanders P; STAR AF II Investigators. Approaches to catheter ablation for persistent atrial fibrillation. N Engl J Med 2015;372(19):1812-22. doi: 10.1056/NEJMoa1408288.

# Table S5. Main characteristics and outcomes of RCTs investigating the role of GP ablation for the treatment of AF

| ***1^st^ Author***  ***(Trial name)*** | ***Journal/year*** | ***Study design*** | ***Pt n°*** | ***Parox***  ***AF%*** | ***Approach*** | ***I° endpoint*** | ***Follow-up duration***  ***Methodology*** | ***Outcomes*** | ***Complications*** | ***Comments*** |
| --- | --- | --- | --- | --- | --- | --- | --- | --- | --- | --- |
| ***Randomised Clinical Studies RCT*** | | | | | | | | | | |
| ***Kim***  ***GANGLIA-AF*** | Hrthm  2021 | RCT | 102 | 100% | Endo TC- GPA  (ET-GPA)  vs.  PVI | Documented AT/AF > 30sec sec after 3 months blanking | 12 months  3-monthly 48h Holter | Freedom of recurrence was 50% with GPA vs 64% with PVI (P= 0.09).  58% freedom in Ectopy-triggering GPA | - 1 tamponade during GPA requiring  pericardiocentesis.  - no major complications with  PVI.  - 7 GPA pts hospitalized for pericarditis symptoms vs.  1 PVI pt (P =0.06). | RF time was shorter in GPA (22.9±9.8 min) than in PVI (38±14.4 min) (P <0.0001); also the use of AAD (55.5% vs 36%; P.05), and redoablation (31% after GPA and 24% after  PVI (P .53). |
| ***Sandler***  (same researchers of above study possible overlap) | JCE  2021 | RCT  underpowered, proof-of concept study | 67 | 100% | Endo TC of ET-GPA  vs.  PVI | Documented AT/AF > 30sec sec after 3 months blanking | 12 months  3-monthly 48h Holter | Freedom of AT/AF was 61% and 49% in PVI and ET-GPA, respectively (log-rank P = .27). | 1 groin hematoma conservatively managed in the PVI group. In the ET‐GPA group, there was one patient with transient phrenic nerve (< 24 h). | Duration of procedure was 3.7 ± 1.0 and 3.3 ± 0.7 h in ET-GPA group and PVI, respectively (P = .07). |
| ***Berger***  ***AFACT Study***  (same study below but with longer follow-up) | JACC EP  2021 | RCT | 240 | 40% | Thoracoscopic surgery:  PVI + LAA exclusion (+Dallas lesion for persistent AF)  vs.  ***additional*** GPA (+Marshal) | Freedom of AT/AF > 30 sec | 24 months  Periodic Holter ECG | Freedom of AT/AF did not differ significantly  between the GP group (55.6%) and control group (56.1%) (p =0.91), | No additional complications occurred in the 2^nd^ year | Additional GPA during thoracoscopic surgery for advanced AF does not affect freedom of AF  Recurrence.  As GPA is associated with more major procedural complications, it should not routinely be performed |
| ***Driessen***  ***AFACT Study*** | JACC  2016 | RCT | 240 | 40% | Thoracoscopic surgery:  PVI + LAA exclusion (+Dallas lesion for persistent AF)  vs.  ***additional*** GPA (+Marshal) | Freedom of AT/AF > 30 sec | 12 months  3-monthly Holter 24h | no AT/AF recurrences were  observed in 70.9% and 68.4% of patients in the GPA  and control groups (P= 0.696) | Major bleeding in 9 patients (all GPA group; p < 0.001); 8 patients were managed thoracoscopically,  and 1 underwent sternotomy. SND occurred in 12 patients in GPA group and 4 control subjects (p =0.038), and 6 PM were implanted (all in GPA group; P = 0.013). | GPA during thoracoscopic surgery for advanced AF has no detectable effect on AF recurrence  but causes more major adverse events, major bleeding, SND, and PM implantation |
| ***Sakamoto*** | Gen Thorac Cardiovasc Surg  2022 | RCT | 74 | AF+  structural HD  ~20% | Maze  vs.  Maze+GPA |  | 16.3 ± 7.9 months | freedom from AT/AF was 86.8% and 91.4% in the GPA and Maze group, respectively(P = 0.685). | 1 CHF in GPA group, 1 stroke in Maze alone group.  Need for PM implantation did not differ 5.7% (GPA) vs 5.8% | The addition of GPA to the maze procedure does not improve early outcome when treating AF associated with structural heart disease.  *LA dimension was the only predictor of recurrence* |
| ***Mamchur*** | Interventional Medicine & Applied Science 2014 | RCT | 120 | 0% | Endo-TC PVI  vs.  Extended PVI  vs.  GPA |  | 16 months  Periodic Holter ECG  ECG during symptoms | SR off AAD was maintained in: 38% in GPA group,  56% in PVI group, and 69% in extended PVI group. | Not reported | Limited study – no clear endpoint; complications; follow up methodology?! |
| ***Katritsis*** | JACC  2013 | RCT | 242 | 100% | Endo-TC PVI  vs.  GPA  vs.  PVI+GPA | Freedom of AF/AT > 30 sec | 24 months  Monthly visit +ECG  - At symptom transtelefonic monitor  - ILR in 50% of patients | Freedom from AT/AF was achieved in 56%, 48%, and 74% of patients in the PVI, GPA, and PVI+GPA  groups, respectively (p = 0.004 by log-rank test) | Only 1 tamponade +pericardiocentesis in the PVI group | PVI+GPA strategy compared with PVI alone yielded  a HR of 0.53 (95% confidence interval: 0.31 to 0.91; p ¼ 0.022) for recurrence of AT/AF.  Addition of GPA to PVI confers a significantly higher success rate compared with either PVI or GP alone in PAF patients |
|  |  |  |  |  |  |  |  |  |  |  |
| ***Pokushalov*** | Hrthm  2013 | RCT | 264 | 0% | Endo-TC PVI + GPA  vs.  PVI + lines | Freedom of AF/AT > 0.5% | 3 years  ILR | 34% of the patients with PVI+LL and 49% of the patients with PVI+GP maintained SR (*P* =.035) | AFL was more frequent in PVI+LL group than in PVI+GP group (18% vs 6%; *P* = .002).  Tampondae/pericardiocentesis in 1 PVI pt and 2 GPA pts | PVI+GPA confers superior clinical results with less ablation-related LA flutter and reduced AF recurrence compared to PVI+LL ablation at 3 years of follow-up |
| ***Katritsis*** | Hrthm  2011 | RCT | 67 | 100% | Endo-TC PVI  vs.  PVI+GPA | Freedom of AF/AT > 30 sec | 12 months  ECG +Holter 48h every 3 months | 60.6% patients in PVI group and 85.3% patients in the GPA+PVI group remained arrhythmia-free (log rank test, *P* = .019). | Only 1 tamponade +pericardiocentesis in the GPA group | Addition of GPA to PVI confers significantly better outcomes than PVI alone in PAF |
| ***Pokushalov***  *(no PVI control group!)* | Hrthm 2009 | RCT | 80 | 100% | Endo-TC anatomical GPA  vs.  selective GPA (guided by HF stimulation) | symptomatic AT/AF recurrence | 13.1 (± 1.9) months | 42.5% of patients with selective GPA and 77.5% of patients with anatomic GPA were free of symptomatic PAF (*P* = .02) | ? | Selective GPA directed by HF stimulation does not eliminate PAF in the majority of patients. An anatomic approach for regional ablation at the sites of GP confers better results |
| ***Non-randomised Clinical Studies*** | | | | | | | | | | |
| ***Morita*** | HrthmO2  2022 | retrospective  observational | 225 | 100% | Endo TC PVI  vs.  GPA (with or without SVC-Ao)+PVI | palpitations and/or documentation of AT/AF > 30 sec | Ambulatory visit +ECG every 3 months.  Holter 24h ECG between 3-6 months, then arbitrary at symptoms or physician discretion | PVI+GPA provided higher PAF suppression than a PVI alone during more >4 years of follow-up (56.7% vs 38.2%, odds ratio: 0.42, 95% CI: 0.23–0.76, *P* < .05) | 3 patients underwent PM implantations for SND (2 in PVI alone group and 1 in GPA group, NS) | the effect of an empiric SVC-Ao GPA could not be appreciated, suggesting little effect on suppressing non-PV foci. |
| ***Barta*** | CardioThorac Surg 2017 | retrospective | 100 | 42% | Maze  Vs.  Maze+GPA | Freedom of AF/AT > 30 sec | 12 months  Amb ECG  24h Holter | patients with SR has shown comparable values in both groups (Group GPA - 93.75%, Group LA - 86.67%, P = 0.485); also without AAD (Group GPA - 50%, Group LA - 47%, P = 0.306). | No complications related to the ablation procedure were  observed peroperatively or postoperatively. | GPA in addition to surgical LA did not influence the freedom of AF at 12-month follow-up. |
| ***Gelsomino*** | In J Cardiol  2015 | retrospective | 519 | 0% | Maze  Vs.  Maze+GPA | Freedom of AF/AT > 30 sec  off AAD | 36 months  Routine ECG  Annual Holter | SR percentage off-AAD did not improve with the addition of GPA (67.8% vs. 75.5%; P= 0.08). | No difference in hospital stay or need for definitive PM | absence of GPA was not a significant predictor of AT/AF recurrence (P = 0.12). |
| ***Xu*** | PACE 2017 | retrospective | 123 | 100%  (redo) | Endo-TC  Re-PVI  vs.  Re-PVI+GPA | Freedom of AF/AT > 30 sec  off AAD | 12 months  Visit + Holter monitoring every 3 months | 90.6% GPA vs 78% re-PVI alone group remained in SR off AADs (P = 0.045) | 1 tamponde /pericardiocentesis in GPA group; minor vasc complications in 1 GPA pt and in 2 re-PVI pt | GPA conferred incremental benefit when performed in addition to re-PVI in patients with PAF recurrence |
| ***Watanabe*** | Surgery Today  2018 | retrospective | 79 | 24% | Maze  vs.  Maze+GPA | Freedom of AF/AT > 30 sec | 12 months  Ambulatory ECG  24h Holter | freedom from AF at 1 year with or without GPA groups was similar: 77% and 75%, respectively. |  | Addition of GPA to Cox maze IV did not reduce AFrecurrence.  Non-significant trend to reduce adverse events in GPA group (composite HF hospitalization, stroke, all-cause mortality) |
| ***Mikhaylov*** | Europace 2011 | Case-control study | 70 | 100% | Endo-TC  GPA  Vs.  PVI | Freedom of AF/AT > 30 sec | 36.3+2.3 months  ECG + Holter every 3 months 1st year, then every 6 months | Freedom of AT/AF was 34.3% in GPA 65.7% in CPVI  (P = 0.009). | 1 PV stenosis in each group; 1 tamponade requiring surgery in GPA group | Early arrhythmia recurrences and anatomic GPA were  independent predictors of late recurrence |
| ***Onorati*** | J Thorac Cardiovasc Surg 2008 | retrospective | 75 | 0% | Maze  vs.  Maze+GPA | Freedom of AF/AT > 30 sec | 12 months  Ambulatory ECG  24h Holter | freedom from AT/AF off AAD was higher in GPA (92.9% +/- 6.9% vs 62.5% +/- 9.4%; P =0.023). |  | No differences in freedom from CHF (group A: 83.4% +/- 7.0% vs. group B: 93.5% +/- 4.4%; P = .978) and hospital readmission (group A: 84.2% +/- 5.9% vs group B: 92.6% +/- 5.1%; P = .376). |

**References of GP ablation**

1- Kim MY, Coyle C, Tomlinson DR, Sikkel MB, Sohaib A, Luther V, Leong KM, Malcolme-Lawes L, Low B, Sandler B, Lim E, Todd M, Fudge M, Wright IJ, Koa-Wing M, Ng FS, Qureshi NA, Whinnett ZI, Peters NS, Newcomb D, Wood C, Dhillon G, Hunter RJ, Lim PB, Linton NWF, Kanagaratnam P. Ectopy-triggering ganglionated plexuses ablation to prevent atrial fibrillation: GANGLIA-AF study. Heart Rhythm. 2022 Apr;19(4):516-524. doi: 10.1016/j.hrthm.2021.12.010. Epub 2021 Dec 13. PMID: 34915187; PMCID: PMC8976158.

2- Sandler B, Kim MY, Sikkel MB, Malcolme-Lawes L, Koa-Wing M, Whinnett ZI, Coyle C, Linton NWF, Lim PB, Kanagaratnam P; other members of the Imperial College London, Cardiovascular Study Group/Consortium. Targeting the ectopy-triggering ganglionated plexuses without pulmonary vein isolation prevents atrial fibrillation. J Cardiovasc Electrophysiol. 2021 Feb;32(2):235-244. doi: 10.1111/jce.14870. Epub 2021 Jan 19. PMID: 33421265; PMCID: PMC8611799.

3- Berger WR, Neefs J, van den Berg NWE, Krul SPJ, van Praag EM, Piersma FR, de Jong JSSG, van Boven WP, Driessen AHG, de Groot JR. Additional Ganglion Plexus Ablation During Thoracoscopic Surgical Ablation of Advanced Atrial Fibrillation: Intermediate Follow-Up of the AFACT Study. JACC Clin Electrophysiol. 2019 Mar;5(3):343-353. doi: 10.1016/j.jacep.2018.10.008. Epub 2018 Nov 28. PMID: 30898238.

4- Driessen AHG, Berger WR, Krul SPJ, van den Berg NWE, Neefs J, Piersma FR, Chan Pin Yin DRPP, de Jong JSSG, van Boven WP, de Groot JR. Ganglion Plexus Ablation in Advanced Atrial Fibrillation: The AFACT Study. J Am Coll Cardiol. 2016 Sep 13;68(11):1155-1165. doi: 10.1016/j.jacc.2016.06.036. PMID: 27609676.

5- Sakamoto SI, Ishii Y, Otsuka T, Mitsuno M, Shimokawa T, Isomura T, Yaku H, Komiya T, Matsumiya G, Nitta T. Multicenter randomized study evaluating the outcome of ganglionated plexi ablation in maze procedure. Gen Thorac Cardiovasc Surg. 2022 Oct;70(10):908-915. doi: 10.1007/s11748-022-01820-8. Epub 2022 Apr 27. PMID: 35476249.

6- Mamchur SE, Mamchur IN, Khomenko EA, Bokhan NS, Scherbinina DA. 'Electrical exclusion' of a critical myocardial mass by extended pulmonary vein antrum isolation for persistent atrial fibrillation treatment. Interv Med Appl Sci. 2014 Mar;6(1):31-9. doi: 10.1556/IMAS.6.2014.1.5. Epub 2014 Mar 14. PMID: 24672673; PMCID: PMC3955813.

7- Katritsis DG, Pokushalov E, Romanov A, Giazitzoglou E, Siontis GC, Po SS, Camm AJ, Ioannidis JP. Autonomic denervation added to pulmonary vein isolation for paroxysmal atrial fibrillation: a randomized clinical trial. J Am Coll Cardiol. 2013 Dec 17;62(24):2318-25. doi: 10.1016/j.jacc.2013.06.053. Epub 2013 Aug 21. PMID: 23973694.

8- Pokushalov E, Romanov A, Katritsis DG, Artyomenko S, Shirokova N, Karaskov A, Mittal S, Steinberg JS. Ganglionated plexus ablation vs linear ablation in patients undergoing pulmonary vein isolation for persistent/long-standing persistent atrial fibrillation: a randomized comparison. Heart Rhythm. 2013 Sep;10(9):1280-6. doi: 10.1016/j.hrthm.2013.04.016. Epub 2013 Apr 19. PMID: 23608592.

9- Katritsis DG, Giazitzoglou E, Zografos T, Pokushalov E, Po SS, Camm AJ. Rapid pulmonary vein isolation combined with autonomic ganglia modification: a randomized study. Heart Rhythm. 2011 May;8(5):672-8. doi: 10.1016/j.hrthm.2010.12.047. Epub 2010 Dec 31. PMID: 21199686.

10- Pokushalov E, Romanov A, Shugayev P, Artyomenko S, Shirokova N, Turov A, Katritsis DG. Selective ganglionated plexi ablation for paroxysmal atrial fibrillation. Heart Rhythm. 2009 Sep;6(9):1257-64. doi: 10.1016/j.hrthm.2009.05.018. Epub 2009 May 20. PMID: 19656736.

11- Morita N, Iida T, Nanao T, Ushijima A, Ueno A, Ikari Y, Kobayashi Y. Effect of ganglionated plexi ablation by high-density mapping on long-term suppression of paroxysmal atrial fibrillation - The first clinical survey on ablation of the dorsal right plexusus. Heart Rhythm O2. 2021 Jul 15;2(5):480-488. doi: 10.1016/j.hroo.2021.07.002. Erratum in: Heart Rhythm O2. 2021 Nov 18;2(6Part A):668. PMID: 34667963; PMCID: PMC8505203.

12- Bárta J, Brát R. Assessment of the effect of left atrial cryoablation enhanced by ganglionated plexi ablation in the treatment of atrial fibrillation in patients undergoing open heart surgery. J Cardiothorac Surg. 2017 Aug 17;12(1):69. doi: 10.1186/s13019-017-0625-1. PMID: 28818088; PMCID: PMC5561587.

13- Gelsomino S, Lozekoot P, La Meir M, Lorusso R, Lucà F, Rostagno C, Renzulli A, Parise O, Matteucci F, Gensini GF, Crjins HJ, Maessen JG. Is ganglionated plexi ablation during Maze IV procedure beneficial for postoperative long-term stable sinus rhythm? Int J Cardiol. 2015 Aug 1;192:40-8. doi: 10.1016/j.ijcard.2015.04.259. Epub 2015 May 1. PMID: 25985014.

14- Xu FQ, Yu RH, Guo JJ, Bai R, Liu N, An YI, Guo XY, Tang RB, Long DY, Sang CH, DU X, Dong JZ, Ma CS. Catheter Ablation of Recurrent Paroxysmal Atrial Fibrillation: Is Gap-Closure Combining Ganglionated Plexi Ablation More Effective? Pacing Clin Electrophysiol. 2017 Jun;40(6):672-682. doi: 10.1111/pace.13064. Epub 2017 May 16. PMID: 28251658.

15- Watanabe M, Kohno H, Kondo Y, Ueda H, Ishida K, Tamura Y, Abe S, Sato Y, Kobayashi Y, Matsumiya G. Is ganglionated plexus ablation effective for treating atrial fibrillation? Surg Today. 2018 Sep;48(9):875-882. doi: 10.1007/s00595-018-1672-5. Epub 2018 May 18. PMID: 29777366.

16- Mikhaylov E, Kanidieva A, Sviridova N, Abramov M, Gureev S, Szili-Torok T, Lebedev D. Outcome of anatomic ganglionated plexi ablation to treat paroxysmal atrial fibrillation: a 3-year follow-up study. Europace. 2011 Mar;13(3):362-70. doi: 10.1093/europace/euq416. Epub 2010 Nov 17. PMID: 21088001.

17- Onorati F, Curcio A, Santarpino G, Torella D, Mastroroberto P, Tucci L, Indolfi C, Renzulli A. Routine ganglionic plexi ablation during Maze procedure improves hospital and early follow-up results of mitral surgery. J Thorac Cardiovasc Surg. 2008 Aug;136(2):408-18. doi: 10.1016/j.jtcvs.2008.03.022. Epub 2008 May 19. PMID: 18692650.

# Table S6. Comparison of recently published recommendation schemes of guideline and consensus documents on catheter ablation of AF from the European Society of Cardiology (ESC), and the American College of Cardiology (ACC)/ American Heart Association (AHA)/ Heart Rhythm Society /HRS, and from the European Heart Rhythm Association/HRS/ Asian Pacific HRS, and the Latin American HRS with the present ECAS guideline document

|  | **ECAS**  **2024**  **AF ABL GLs** | **ESC**  **2024**  **AF GLs**^1^ | **ACC/AHA/ACCP/HRS**  **2023**  **AF GLs**^2^ | **EHRA/HRS/**  **APHRS/LAHRS**  **2024**  **AF ABL GLs**^3^ |
| --- | --- | --- | --- | --- |
| **Recommendations Classes and Levels of Evidence** | | | | |
| **Classification scheme** | **Class I**  **Class II**  **Class III** | **Class I**  Level of evidence A Level of evidence B  Level of evidence C  **Class IIa**  Level of evidence A  Level of evidence B  Level of evidence C  **Class IIb**  Level of evidence A  Level of evidence B  Level of evidence C  **Class III**  Level of evidence A  Level of evidence B  Level of evidence C | **Class I**  Level of evidence A  Level of evidence B-R Level of evidence B-NR  Level of evidence C-LD Level of evidence C-EO  **Class IIa**  Level of evidence A  Level of evidence B-R Level of evidence B-NR  Level of evidence C-LD Level of evidence C-EO  **Class IIb**  Level of evidence A  Level of evidence B-R Level of evidence B-NR  Level of evidence C-LD Level of evidence C-EO  **Class III no benefit**  Level of evidence A  Level of evidence B-R Level of evidence B-NR  Level of evidence C-LD Level of evidence C-E**O**  **Class III harm**  Level of evidence A  Level of evidence B-R Level of evidence B-NR  Level of evidence C-LD Level of evidence C-E**O** | **Advice to do**  Type of Evidence META  Type of Evidence RAND  Type of Evidence OBS  Type of Evidence OPN  **May be appropriate to do**  Type of Evidence META  Type of Evidence RAND  Type of Evidence OBS  Type of Evidence OPN  **Uncertainty**  Type of Evidence META  Type of Evidence RAND  Type of Evidence OBS  Type of Evidence OPN  **Avoid to do**  Type of Evidence META  Type of Evidence RAND  Type of Evidence OBS  Type of Evidence OPN |
| **CA Indications** | | | | |
| **CA for drug-refractory or drug-intolerant AF** | **Class I** | **Class I A** | **Class I A** | **Advice to do META** |
| **CA as first-line therapy or early strategy** | **Class I**  For paroxysmal AF using CB  **Class II**  for paroxysmal AF using RF | **Class I A**  for paroxysmal AF  **Class IIb C**  for persistent AF | **Class I A**  for symptomatic paroxysmal AF in selected patients (generally younger and with less co-morbidities)  **Class IIa B-R**  for symptomatic paroxysmal or persistent AF other than young or with few comorbidities | **Advice to do META**  in recurrent paroxysmal AF  **Uncertainty OPN**  in persistent AF |
| **CA for asymptomatic AF** | Cannot be addressed at present | Not Classified: | **Class IIb B-NR**  in selected patients to reduce AF progression and complications | **Uncertainty OPN** |
| **CA for AF in HF** | **Class I** | **Class IIa B**  **Class I B**  for tachycardia-induced cardiomyopathy | **Class IA**  for HFrEF  **Class IIa B-NR**  for HFpEF and symptomatic AF | **May be appropriate to do META**  for HFrEF  **Advice to do META**  for tachycardiomyopathy |
| **CA for AF in End-stage HF** | **Class II** | Not classified | Not classified | Not classified |
| **CA for AF and SSS**  (to avoid PM implantation) | Cannot be classified at present | **Class IIa C** | Not classified | **May be appropriate to do**  **OBS** |
| **CA for recurrent AF after CA** | Cannot be classified at present | **Class IIa B**  If symptoms improved after the initial AF CA | **Class I B-NR** | Not classified |
| **CA Targets** | | | | |
| **PVI** | **Class I** | Not classified | **Class I A** | **Advice to do META** |
| **Beyond PVI**  **ABL Targets:** |  | Not classified | **Class IIb B-R**  Benefit of ablating other non-PVI targets  is UNCERTAIN |  |
| **- non-PV triggers/foci** | Cannot be classified at present |  |  | **Uncertainty OBS:**  for persistent AF  **Advice to do OBS:**  for reproducible non-PV focus initiating AF during CA procedure |
| **- Lines** | **Class III**  no benefit |  |  | **Advice to do OBS***  when performed:  conduction block should be documented |
| **- CFAE** | **Class III**  no benefit |  | **Class IIb B-R** | Not classified |
| **- GP** | **Class III**  no benefit |  | Not classified | Not classified |
| **- LAPW** | **Class III**  no benefit |  | **Class IIb B-R** | **Uncertainty META**  for *redo* persistent AF ABL |
| **- LVA** | **Class II**  -For a paroxysmal AF in elderly patients (65-80 yr).  -For Persistent AF and documented LVA areas |  | **Class IIb B-R** | **Uncertainty META**  for persistent AF |
| **- LAA isolation or ligation** | **Class III**  no benefit |  | Not classified | Not classified  *.* |
| **- Marshal vein** | **Class II**  for persistent drug-refractory AF |  | Not classified | **Uncertainty RAND**  for persistent AF  **May be appropriate to do OBS**  for mitral flutter/block |
| **- MRI-guided (LE/fibrosis)** | **Class III harm** |  | Not classified | **Advice not to do META** |
| **Peri-procedural Anticoagulation** | | | | |
| **Uninterrupted anticoagulation** | **Class II**  Uninterrupted DOAC reduces major bleeding vs. uninterrupted VKA  **Class II**  Uninterrupted VKA is superior to interrupted VKA with Heparin bridging | **Class I A**  Uninterrupted OAT is recommended to prevent peri-procedural ischaemic stroke and thromboembolism | **Class I A**  for DOAC  **Class I B-NR**  for VKA (goal INR 2.0-3.0) | **Advice to do META**  For either DOACs or VKA |
| **Minimally interrupted DOAC** | **Class II**  Comparable to uninterrupted DOAC for bleeding and clinical stroke  **Class II**  May increase silent stroke post-ablation  **Class II**  Dabigatran reduces bleeding vs. uninterrupted VKA | Not classified | **Class I A**  Comparable to uninterrupted DOAC recommendation | **May be appropriate to Do META** |
| **Interrupted DOAC 24h + Heparin bridging** | **Class II**  Comparable to uninterrupted DOAC or uninterrupted VKA | Not classified | Not classified | Not classified |
| **Intraprocedural ACT Target > 300 s** | **Class II** | Not classified | Not classified | **Advice to do OBS** |
| **Short-term OAT post-ABL** | **Class II**  2-3 months | **Class I C**  ≥2 months | **Class I B-NR**  ≥3 months | **Advice to do OPN**  ≥2 months |
| **Long-term discontinuation post-ABL**  **vs. OAT** | **Class II** | **Class I C**  Continuation of OAT is based on individual risk CHA2DS2VA rather than perceived CA clinical success | **Class I B-NR**  Continuation of long-term OAT should be dictated according to the patients’ stroke risk (e.g., CHA2DS2-VASc  score ≥2 ‡) | **Uncertainty OPN**  Using shared decision-making, discontinuation of OAT may be reasonable 12 months post-ABL  in patients with CHA2DS2-VASc score 1 in males and 2 in females in the absence of clinical symptoms or documented AF recurrence when patients and their physician are committed to long-term rhythm monitoring |

Abbreviations: AF: atrial fibrillation; CA: catheter ablation; HF: heart failure; HFpEF: heart failure with preserved ejection fraction; HFrEF: heart failure with reduced ejection fraction; OAT: oral anticoagulation therapy; DOAC: direct oral anticoagulants; SSS: sick sinus syndrome; PM: pacemaker; FU: follow-up; ABL: ablation; PV: pulmonary veins; PVI: pulmonary vein isolation; CFAE: complex fragmented atrial electrograms; LE: late enhancement; MRI: magnetic resonance imaging; ACT: activated clotting time; VKA: vitamin K antagonists; LAA: left atrial appendage; LVA: low-voltage area; LAPW: left atrial posterior wall; NA: not addressed; GP: ganglionated plexi

# Reference

# 1. Ic VG, M R, Kv B, et al. 2024 ESC Guidelines for the management of atrial fibrillation developed in collaboration with the European Association for Cardio-Thoracic Surgery (EACTS). *Eur Heart J*. 2024;45(36). doi:10.1093/eurheartj/ehae176

# 2. Joglar JA, Chung MK, Armbruster AL, et al. 2023 ACC/AHA/ACCP/HRS Guideline for the Diagnosis and Management of Atrial Fibrillation: A Report of the American College of Cardiology/American Heart Association Joint Committee on Clinical Practice Guidelines. *Circulation*. 2024;149(1):e1-e156. doi:10.1161/CIR.0000000000001193

# 3. Tzeis S, Gerstenfeld EP, Kalman J, et al. 2024 European Heart Rhythm Association/Heart Rhythm Society/Asia Pacific Heart Rhythm Society/Latin American Heart Rhythm Society expert consensus statement on catheter and surgical ablation of atrial fibrillation. *Eur Eur Pacing Arrhythm Card Electrophysiol J Work Groups Card Pacing Arrhythm Card Cell Electrophysiol Eur Soc Cardiol*. 2024;26(4):euae043. doi:10.1093/europace/euae043

# Table S7. Main characteristics of ongoing RCTs investigating the role of linear ablation (including LPW isolation) for the treatment of AF

| **NCT Number** | **Study Title** |
| --- | --- |
| NCT03920917 | Cryoballoon Pulmonary Vein Isolation vs. Radiofrequency Pulmonary Vein Isolation With Additional Right Atrial Linear Ablation for Paroxysmal Atrial Fibrillation: Prospective Randomized Trial (CRAPAF Trial) |
| NCT02721121 | Comparison of Circumferential Pulmonary Vein Isolation Alone Versus Linear Ablation in Addition to Circumferential Pulmonary Vein Isolation for Catheter Ablation in Persistent Atrial Fibrillation: Prospective Randomized Controlled Trial |
| NCT03920891 | Cryoballoon Pulmonary Vein Isolation vs. Radiofrequency Pulmonary Vein Isolation With Additional Right Atrial Linear Ablation for Valvular Atrial Fibrillation: Prospective Randomized Trial (CRAVA Trial) |
| NCT03682887 | Cryoballoon Pulmonary Vein Isolation vs. Cryoballoon Pulmonary Vein Isolation With Additional Right Atrial Linear Ablation for Persistent Atrial Fibrillation (CRARAL Trial) |
| NCT05468528 | Clinical Value of Linear Ablation Without Pulmonary Vein Isolation in Persistent Atrial Fibrillation |
| NCT04206982 | MARSHALL PLAN Vs. Pulmonary Veins Isolation Monocentric Trial |
| NCT03998956 | High-density Mapping-guided bOx Isolation and subsTrate Ablation |
| NCT02892162 | Catheter Ablation Therapy for Persistent Atrial Fibrillation |
| NCT05780996 | Efficacy of Anterior Wall PVI With QDOT Catheter - Intraprocedural Comparison Between vHPSD and HPSD Ablation. |
| NCT03295422 | Comparison of Two Pulmonary Vein Ablation Techniques for Persistent AF |
| NCT04428944 | STrategies for Catheter Ablation of peRsistent Atrial Fibrlllation |

# Table S8. Main characteristics of ongoing RCTs investigating the role of CFAE ablation for the treatment of AF

| **NCT Number** | **Study Title** |
| --- | --- |
| NCT03989726 | Efficacy of High Density Voltage and Fractionation Map Guided Ablation in Patients With Atrial Fibrillation |
| NCT05426603 | Waveform Periodicity Analysis of Complex Fractionated Electrograms in Patients With Persistent Atrial Fibrillation |
| NCT04056390 | Ablation STrategies for Repeat PrOcedures in Patients With Atrial Fibrillation Recurrences |
| NCT05496088 | Persistent AF Catheter Ablation: Re-PVI vs. Re-PVI + Continuous Complex Activity Mapping and Ablation - AF-CAM |
| NCT02696265 | CFAE/Spatiotemporal Dispersion Guided Ablation Versus PVI Guided Ablation in Persistent AF |
| NCT02929836 | Catheter Ablation of Longstanding Persistent Atrial Fibrillation |

# Table S9. Main characteristics of ongoing RCTs investigating the role of LAA isolation for the treatment of AF

| **NCT Number** | **Study Title** |
| --- | --- |
| NCT04897204 | Left Atrial Appendage Electrical Isolation in Persistent Atrial Fibrillation |
| NCT04056390 | Ablation STrategies for Repeat PrOcedures in Patients With Atrial Fibrillation Recurrences |
| NCT05731882 | First in Man Study of Left Atrial Appendage Pulsed Field Ablation Occluder |
| NCT05723536 | PLAI-AF Trial: Hybrid Endo-epicardial Partial Left Atrial Isolation vs. Endocardial Ablation in Patients With Persistent Atrial Fibrillation |
| NCT05077670 | Hybrid Characterization of Driver Sites During Atrial Fibrillation and Sinus Rhythm |
| NCT03788941 | Left Atrial Appendage Closure in Combination With Catheter Ablation |

# Table S10. Efficacy outcomes reported in prospective studies enrolling at least 100 patients undergoing catheter ablation of paroxysmal AF (a), persistent AF (b) and paroxysmal/persistent AF (c)

# Table S10. Safety outcomes reported in prospective studies enrolling at least 100 patients undergoing catheter ablation of paroxysmal AF (a), persistent AF (b) and paroxysmal/persistent AF (c)

**Reference Efficacy and complication**

1 [Jiang](https://pubmed.ncbi.nlm.nih.gov/?term=Jiang+R&cauthor_id=35939332) [R](https://pubmed.ncbi.nlm.nih.gov/35939332/#full-view-affiliation-1), [Chen](https://pubmed.ncbi.nlm.nih.gov/?term=Chen+M&cauthor_id=35939332) M,[Fan](https://pubmed.ncbi.nlm.nih.gov/?term=Fan+J&cauthor_id=35939332) J,[Yi](https://pubmed.ncbi.nlm.nih.gov/?term=Yi+F&cauthor_id=35939332) F,  [Tang](https://pubmed.ncbi.nlm.nih.gov/?term=Tang+A&cauthor_id=35939332) A, [Liu](https://pubmed.ncbi.nlm.nih.gov/?term=Liu+X&cauthor_id=35939332),[Zhu](https://pubmed.ncbi.nlm.nih.gov/?term=Zhu+W&cauthor_id=35939332) W ,[Liu](https://pubmed.ncbi.nlm.nih.gov/?term=Liu+S&cauthor_id=35939332) S, [Huang](https://pubmed.ncbi.nlm.nih.gov/?term=Huang+X&cauthor_id=35939332) X, [Liu](https://pubmed.ncbi.nlm.nih.gov/?term=Liu+Q&cauthor_id=35939332) Q,[Ju](https://pubmed.ncbi.nlm.nih.gov/?term=Ju+W&cauthor_id=35939332) W,[Zhang](https://pubmed.ncbi.nlm.nih.gov/?term=Zhang+X&cauthor_id=35939332) X, [Li](https://pubmed.ncbi.nlm.nih.gov/?term=Li+J&cauthor_id=35939332) J, [He](https://pubmed.ncbi.nlm.nih.gov/?term=He+J&cauthor_id=35939332) J, [Shi](https://pubmed.ncbi.nlm.nih.gov/?term=Shi+L&cauthor_id=35939332) L, [Zhou](https://pubmed.ncbi.nlm.nih.gov/?term=Zhou+G&cauthor_id=35939332) G, [Wang](https://pubmed.ncbi.nlm.nih.gov/?term=Wang+Y&cauthor_id=35939332) Y, [Fu](https://pubmed.ncbi.nlm.nih.gov/?term=Fu+G&cauthor_id=35939332) G, [Jian](https://pubmed.ncbi.nlm.nih.gov/?term=Jiang+C&cauthor_id=35939332) C Efficacy of ablation index-guided pulmonary vein isolation in patients with paroxysmal atrial fibrillation Pacing Clin Electrophysiol 2022 Oct;45(10):1186-1193. doi: 10.1111/pace.14578.Epub 2022 Sep 1.

2 S, Steinberg JS. Ganglionated plexus ablation vs linear ablation in patients undergoing pulmonary vein isolation for persistent/long-standing persistent atrial fibrillation: a randomized comparison. Heart Rhythm. 2013 Sep;10(9):1280-6. doi: 10.1016/j.hrthm.2013.04.016. Epub 2013 Apr 19. PMID: 23608592.

3 Kuck KH, Lebedev DS, Mikhaylov EN, Romanov A, Geller L, Kalejs O, Neumann T, Davtyan K, On YK, Popov S, Bongiorni MG, Schluter M, Willems S, Ouyang F. Catheter ablation or medical therapy to delay progression of atrial fibrillation: the randomized controlled atrial fibrillation progression trial (ATTEST). Europace 2021; 23:362-369°. doi:10.1093/euaa298

4 Andrade JG, Champagne J, Dubuc M, Deyell MW, Verma A, Macle L, Leong-Sit P, Novak P, Badra-Verdu M , Sapp J, Mangat I, Khoo C, Steinberg C, Bennett MT, Tang, ASL Khairy P; CIRCA-DOSE Study Investigators Cryoballoon or Radiofrequency Ablation for Atrial Fibrillation Assessed by Continuous Monitoring: A Randomized Clinical Trial Circulation 2019 Nov 26;140(22):1779-1788 .doi: 10.1161/ CIRCULATIONAHA. 119.042622. Epub 2019 Oct 21

Kuck KH, Brugada J, Fürnkranz A , Metzner A , Ouyang F, Chun KRJ, Elvan A , Arentz T , Bestehorn K, Pocock SJ, Albenque JP, Tondo C; FIRE AND ICE Investigators. Cryoballoon or Radiofrequency Ablation for Paroxysmal Atrial Fibrillation. N Engl J Med. 2016;374(23):2235-2245. doi:10.1056/NEJMoa1602014

6 Luik A, Radzewitz A, Kieser M, Walter M, Bramlage P, Hörmann P, Schmidt K, Horn N, Brinkmeier-Theofanopoulou M, Kunzmann K, Riexinger T, Schymik G, Merkel M, Schmitt C. [Cryoballoon Versus Open Irrigated Radiofrequency Ablation in Patients With Paroxysmal Atrial Fibrillation: The Prospective, Randomized, Controlled, Noninferiority FreezeAF Study.](https://pubmed.ncbi.nlm.nih.gov/26283655/) Circulation. 2015 Oct 6;132(14):1311-9. doi: 10.1161/CIRCULATIONAHA.115.016871. Epub 2015 Aug 17.

7 Reddy VY, Dukkipati SR, Neuzil P, Natale A, Albenque JP, Kautzner J, Shah D, Michaud G, Wharton M, Harari D, Mahapatra S, Lambert H, Mansour M.[Randomized, Controlled Trial of the Safety and Effectiveness of a Contact Force-Sensing Irrigated Catheter for Ablation of Paroxysmal Atrial Fibrillation: Results of the TactiCath Contact Force Ablation Catheter Study for Atrial Fibrillation (TOCCASTAR) Study.](https://pubmed.ncbi.nlm.nih.gov/26260733/) Circulation. 2015 Sep 8;132(10):907-15. doi: 10.1161/CIRCULATIONAHA.114.014092. Epub 2015 Aug 10. PMID: 26260733 Clinical Trial.

8 McLellan A, Ling LH, Azzopardi S,Geraldine A. Lee GA,Lee G, Kumar S, Wong MCG,  Walters TE, Lee JM, Looi KL, Halloran K, Stiles MK, Lever NA, Fynn SP, Heck, Sanders P, Morton JP, Kalman JM, Kistler PM. A minimal or maximal ablation strategy to achieve pulmonary vein isolation for paroxysmal atrial fibrillation: a prospective multi-centre randomized controlled trial (the Minimax study) Eur Heart J. 2015 Jul 21;36(28):1812-21. doi: 10.1093/eurheartj/ehv139. Epub 2015 Apr 28.

9 Driessen AHG, Berger WR, Krul SPJ, van den Berg NWE, Neefs J, Piersma FR, Chan Pin Yin DRPP, de Jong JSSG, van Boven WP, de Groot JR. Ganglion Plexus Ablation in Advanced Atrial Fibrillation: The AFACT Study. J Am Coll Cardiol. 2016 Sep 13;68(11):1155-1165. doi: 10.1016/j.jacc.2016.06.036. PMID: 27609676.

10 Dukkipati SR , Cuoco F , Kutinsky I, Aryana A , Bahnson TD , Lakkireddy D , Woollett I, Issa ZF, Natale A, Reddy VY; HeartLight Study Investigators. Pulmonary Vein Isolation Using the Visually Guided Laser Balloon: A Prospective, Multicenter, and Randomized Comparison to Standard Radiofrequency Ablation. J Am Coll Cardiol. 2015;66(12):1350-1360. doi:10.1016/j.jacc.2015.07.036

11 Reddy VY, Gerstenfeld EP, Natale A, Whang W, Cuoco FA, Patel C, Mountantonakis SE, Gibson DN, Harding JD, Ellis CR, Ellenbogen KA, DeLurgio DB, Osorio J, Achyutha AB, Schneider CW, Mugglin AS, Albrecht EM, Stein KM, Lehmann JW, Mansour M; ADVENT Investigators. Pulsed Field or Conventional Thermal Ablation for Paroxysmal Atrial Fibrillation. N Engl J Med. 2023 Nov 2;389(18):1660-1671. doi: 10.1056/NEJMoa2307291. Epub 2023 Aug 27. PMID: 37634148

12 Andrade JG, Deyell MW, Macle L, Wells GA, Bennett M, Essebag V, Champagne J, Roux JF, Yung D, Skanes A, Khaykin Y, Morillo C, Jolly U, Novak P, Lockwood E, Amit G, Angaran P, Sapp J, Wardell S, Lauck S, Cadrin-Tourigny J, Kochhäuser S, Verma A; EARLY-AF Investigators. Progression of Atrial Fibrillation after Cryoablation or Drug Therapy. N Engl J Med. 2023 Jan 12;388(2):105-116. doi: 10.1056/NEJMoa2212540. PMID: 36342178.

13  [Wazni](https://pubmed.ncbi.nlm.nih.gov/?term=Wazni+OM&cauthor_id=33197158) OM,  [Dandamudi](https://pubmed.ncbi.nlm.nih.gov/?term=Dandamudi+G&cauthor_id=33197158)G,  [Sood](https://pubmed.ncbi.nlm.nih.gov/?term=Sood+N&cauthor_id=33197158) N,  [Hoyt](https://pubmed.ncbi.nlm.nih.gov/?term=Hoyt+R&cauthor_id=33197158) R, [Tyler](https://pubmed.ncbi.nlm.nih.gov/?term=Tyler+J&cauthor_id=33197158) J,  [Durrani](https://pubmed.ncbi.nlm.nih.gov/?term=Durrani+S&cauthor_id=33197158) S,  [Niebauer](https://pubmed.ncbi.nlm.nih.gov/?term=Niebauer+M&cauthor_id=33197158) M,  [Makati](https://pubmed.ncbi.nlm.nih.gov/?term=Makati+K&cauthor_id=33197158) K ,  [Halperin](https://pubmed.ncbi.nlm.nih.gov/?term=Halperin+B&cauthor_id=33197158) B,  [Gauri](https://pubmed.ncbi.nlm.nih.gov/?term=Gauri+A&cauthor_id=33197158) A,  [Morales](https://pubmed.ncbi.nlm.nih.gov/?term=Morales+G&cauthor_id=33197158)  G,  [Shao](https://pubmed.ncbi.nlm.nih.gov/?term=Shao+M&cauthor_id=33197158) M,  [Cerkvenik](https://pubmed.ncbi.nlm.nih.gov/?term=Cerkvenik+J&cauthor_id=33197158) J,  [Kaplon](https://pubmed.ncbi.nlm.nih.gov/?term=Kaplon+RE&cauthor_id=33197158) RE,  [Nissen](https://pubmed.ncbi.nlm.nih.gov/?term=Nissen+SE&cauthor_id=33197158) SE; [STOP AF First Trial Investigators](https://pubmed.ncbi.nlm.nih.gov/?term=STOP+AF+First+Trial+Investigators%5BCorporate+Author%5D) Cryoballoon Ablation as Initial Therapy for Atrial Fibrillation. N Engl J Med. 2021 Jan 28;384(4):316-324. doi: 10.1056/NEJMoa2029554. Epub 2020 Nov 16.

14 Andrade JG, Wells GA, Deyell MW, Bennett M, Essebag V, Champagne J, Roux JF, Yung D, Skanes A, Khaykin Y, Morillo C, Jolly U, Novak P, Lockwood E, Amit G, Angaran P, Sapp J, Wardell S, Lauck S, Macle L, Verma A; EARLY-AF Investigators. Cryoablation or Drug Therapy for Initial Treatment of Atrial Fibrillation. N Engl J Med. 2021 Jan 28;384(4):305-315. doi: 10.1056/NEJMoa2029980. Epub 2020 Nov 16. PMID: 33197159.

15 Packer DL, Kowal RC, Wheelan KR, Irwin JM, Champagne J, Guerra PG, Dubuc M, Reddy V, Nelson L, Holcomb RG, Lehmann JW, Ruskin JN; STOP AF Cryoablation Investigators. Cryoballoon ablation of pulmonary veins for paroxysmal atrial fibrillation: first results of the North American Arctic Front (STOP AF) pivotal trial.J Am Coll Cardiol. 2013 Apr 23;61(16):1713-23. doi: 10.1016/j.jacc.2012.11.064. Epub 2013 Mar 21.

16 Verma A, Haines DE, Boersma LV , Sood N , Natale A, Marchlinski FE, Calkins H , Sanders P, Packer DL , Kuck KH, Hindricks G , Onal B , Cerkvenik J, Tada H, DeLurgio DB ; PULSED AF Investigators. Pulsed Field Ablation for the Treatment of Atrial Fibrillation: PULSED AF Pivotal Trial. Circulation. 2023;147(19):1422-1432. doi:10.1161/CIRCULATIONAHA.123.063988

17 Dukkipati SR, Cuoco F, Kutinsky I, Aryana A, Bahnson TD, Lakkireddy D, Woollett I, Issa ZF, Natale A, Reddy VY; HeartLight Study Investigators. [Pulmonary Vein Isolation Using the Visually Guided Laser Balloon: A Prospective, Multicenter, and Randomized Comparison to Standard Radiofrequency Ablation.](https://pubmed.ncbi.nlm.nih.gov/26383722/) J Am Coll Cardiol. 2015 Sep 22;66(12):1350-60. doi: 10.1016/j.jacc.2015.07.036.

18 Sohara H, Ohe T, Okumura K, Naito S, Hirao K, Shoda M, Kobayashi Y, Yamauchi Y, Yamaguchi Y, Kuwahara T, Hirayama H, YeongHwa C, Kusano K, Kaitani K, Banba K, Fujii S, Kumagai K, Yoshida H, Matsushita M, Satake S, Aonuma K. [HotBalloon Ablation of the Pulmonary Veins for Paroxysmal AF: A Multicenter Randomized Trial in Japan.](https://pubmed.ncbi.nlm.nih.gov/28007137/) J Am Coll Cardiol. 2016 Dec 27;68(25):2747-2757. doi: 10.1016/j.jacc.2016.10.037.

19 Anter E, Mansour M, Nair DG, Sharma D, Taigen TL , Neuzil P , Kiehl EL, Kautzner J , Osorio J, Mountantonakis S, Natale A, Hummel JD, Amin AK, U Riddiqui UR, Doron H, Reddy VY; SPHERE PER-AF Investigators. Dual-energy lattice-tip ablation system for persistent atrial fibrillation: a randomized trial. Nature Med 2024; doi.org/10.1038/s41591-024-03022-

20 Lakkireddy DR, Wilber, DJ Mittal S, Tschopp D, Ellis CR, Rasekh A, Hounshell T, Evonich R , Chandhok S , Berger RD , Horton R, Hoskins MH, Calkins H , Yakubov SJ, Simons P, Benjamin R Saville BR , Lee RJ; aMAZE Investigators Pulmonary Vein Isolation With or Without Left Atrial Appendage Ligation in Atrial Fibrillation: The aMAZE Randomized Clinical Trial. JAMA 2024 Apr 2;331(13):1099-1108. PMCID: PMC10988350 (available on 2024-10-02) DOI: 10.1001/jama.2024.3026

21 Chen H, Li C, Han B, Xiao F, Yi F, Wei Y, Jiang C, Zou C, Shi L, Ma W, Wang W, Wang Y, Du H, Chen L, Chen M. STABLE-SR-III Investigators. Circumferential pulmonary vein isolation with vs without additional low-voltage-area ablation: a randomized clinical tiral. JAMA Cardiol 2023;8:765-772 - doi:10.1001/jamacardio.2023.1749

22 Kistler PM , Chieng D, Sugumar H, Ling LH, Segan L, Azzopardi S, Al-Kaisey A, Parameswaran R, Anderson RD , Hawson J, Prabhu S, Voskoboinik A, Wong G, Morton JB, Pathik B, McLellan AJ, Lee G, l Wong M, Finch S, Pathak RK, Raja DC, Sterns L, Ginks M, Reid CM, Sanders P, Kalman JM Effect of Catheter Ablation Using Pulmonary Vein Isolation With vs Without Posterior Left Atrial Wall Isolation on Atrial Arrhythmia Recurrence in Patients With Persistent Atrial Fibrillation: The CAPLA Randomized Clinical Trial. JAMA2023 Jan 10;329(2):127-135. doi: 10.1001/jama.2022.23722.

23 Huo Y, Gaspar T, Schoebauer R, Wojcik M, Fiedler L, Roithinger FX, Martinek M, et al. Low-voltage myocardium-guided ablation trial of persistent atrial fibrillation. NEJM Evid 2022(11). DOI: 10.1056/EVIDoa2200141

24 Marrouche NF, Wazni O , McGann C , Greene T, Dean MJ, Dagher L, Kholmovski, Masour M, Marchlinski F, Wilber D, Hindricks G, Mahnkopf C, Wells D, Jais P, Sanders P, Brachmann J, Bax JJ, Morrison-de Boer L, Deneke T, Calkins H, Sohns C, Akoum N ; DECAAF II Investigators Effect of MRI-Guided Fibrosis Ablation vs Conventional Catheter Ablation on Atrial Arrhythmia Recurrence in Patients With Persistent Atrial Fibrillation: The DECAAF II Randomized Clinical Trial. JAMA 2022 Jun 21;327(23):2296-2305. DOI: [10.1001/jama.2022.8831](https://doi.org/10.1001/jama.2022.8831)

25 Valderrábano M, Peterson LE, Swarup V, Schurmann PA, Makkar A, Doshi RN, DeLurgio D, Athill CA, Ellenbogen KA, Natale A, Koneru J, Dave AS, Giorgberidze I, Afshar H, Guthrie ML, Bunge R, Morillo CA, Kleiman NS. Effect of Catheter Ablation With Vein of Marshall Ethanol Infusion vs Catheter Ablation Alone on Persistent Atrial Fibrillation: The VENUS Randomized Clinical Trial. JAMA. 2020 Oct 27;324(16):1620-1628. doi: 10.1001/jama.2020.16195. PMID: 33107945; PMCID: PMC7592031.

26 Di Biase L, Mohanty P, Mohanty S, Santangeli P, Trivedi C, Lakkireddy D, Reddy M, Jais P, Themistoclakis S, Dello Russo A, Casella M, Pelargonio G, Narducci ML, Schweikert R, Neuzil P, Sanchez J, Horton R, Beheiry S, Hongo R, Hao S, Rossillo A, Forleo G, Tondo C, Burkhardt JD, Haissaguerre M, Natale A. Ablation Versus Amiodarone for Treatment of Persistent Atrial Fibrillation in Patients With Congestive Heart Failure and an Implanted Device: Results From the AATAC Multicenter Randomized Trial.Circulation. 2016 Apr 26;133(17):1637-44. doi: 10.1161/CIRCULATIONAHA.115.019406. Epub 2016 Mar 30.

27 Verma A, Jiang CY, Betts TR, Chen J, Deisenhofer I, Mantovan R, Macle L, Morillo CA, Haverkamp W, Weerasooriya R, Albenque JP, Nardi S, Menardi E, Novak P, Sanders P; STAR AF II Investigators Approaches to catheter ablation for persistent atrial fibrillation. N Engl J Med. 2015 May 7;372(19):1812-22. doi: 10.1056/NEJMoa1408288

28 Poole JE, Bahnson TD, Monahan KH, Johnson G, Rostami H, Silverstein AP, Al-Khalidi HR, Rosenberg Y, Mark DB, Lee KL, Packer DL; CABANA Investigators and ECG Rhythm Core Lab. [Recurrence of Atrial Fibrillation After Catheter Ablation or Antiarrhythmic Drug Therapy in the CABANA Trial.](https://pubmed.ncbi.nlm.nih.gov/32586583/) J Am Coll Cardiol. 2020 Jun 30;75(25):3105-3118. doi: 10.1016/j.jacc.2020.04.065.

29 Kobori A, Shizuta S, Inoue K, Kaitani K, Morimoto T, Nakazawa Y, Ozawa T, Kurotobi T, Morishima I, Miura F, Watanabe T, Masuda M, Naito M, Fujimoto H, Nishida T, Furukawa Y, Shirayama T, Tanaka M, Okajima K, Yao T, Egami Y, Satomi K, Noda T, Miyamoto K, Haruna T, Kawaji T, Yoshizawa T, Toyota T, Yahata M, Nakai K, Sugiyama H, Higashi Y, Ito M, Horie M, Kusano KF, Shimizu W, Kamakura S, Kimura T; UNDER-ATP Trial Investigators. [Adenosine triphosphate-guided pulmonary vein isolation for atrial fibrillation: the UNmasking Dormant Electrical Reconduction by Adenosine TriPhosphate (UNDER-ATP) trial.](https://pubmed.ncbi.nlm.nih.gov/26321237/) Eur Heart J. 2015 Dec 7;36(46):3276-87. doi: 10.1093/eurheartj/ehv457. Epub 2015 Aug 30.

30 Kaitani K, Inoue K, Kobori A, Nakazawa Y, Ozawa T, Kurotobi T, Morishima I, Miura F, Watanabe T, Masuda M, Naito M, Fujimoto H, Nishida T, Furukawa Y, Shirayama T, Tanaka M, Okajima K, Yao T, Egami Y, Satomi K, Noda T, Miyamoto K, Haruna T, Kawaji T, Yoshizawa T, Toyota T, Yahata M, Nakai K, Sugiyama H, Higashi Y, Ito M, Horie M, Kusano KF, Shimizu W, Kamakura S, Morimoto T, Kimura T, Shizuta S; EAST-AF Trial Investigators. [Efficacy of Antiarrhythmic Drugs Short-Term Use After Catheter Ablation for Atrial Fibrillation (EAST-AF) trial.](https://pubmed.ncbi.nlm.nih.gov/26417061/) Eur Heart J. 2016 Feb 14;37(7):610-8. doi: 10.1093/eurheartj/ehv501. Epub 2015 Sep 28.

31 Packer DL, Piccini JP, Monahan KH, Al-Khalidi HR, Silverstein AP, Noseworthy PA, Poole JE, Bahnson TD, Lee KL, Mark DB; CABANA Investigators. [Ablation Versus Drug Therapy for Atrial Fibrillation in Heart Failure: Results From the CABANA Trial.](https://pubmed.ncbi.nlm.nih.gov/33554614/) Circulation. 2021 Apr 6;143(14):1377-1390. doi: 10.1161/CIRCULATIONAHA.120.050991. Epub 2021 Feb 8.PMID: 3355461
